# Antimicrobial peptides derived from Nemuri induce ribosomal condensation to inhibit translation

**DOI:** 10.1038/s41467-026-78960-5

**Published:** 2026-10-29

**Authors:** Suguru Nishinami, Moynul Hasan, Tatsuya Niwa, Tomohiro Inui, Takeshi Yokoyama, Hiroto Otsuki, Yuta Ogasawara, Hiroka Sugai, Yuta Otobe, Masato Machida, Mohammed Abdelaziz, Natsumi Ageta-Ishihara, Ryohei Sasaki, Yoshimasa Ishizaki, Kenji Iwasaki, Yuko Fujioka, Tomokazu Ohishi, Shinji Kajimoto, Takakazu Nakabayashi, Yoshikazu Tanaka, Hikari Yoshitane, Hiroyuki Kumeta, Amita Sehgal, Masayuki Igarashi, Nobuo N. Noda, Hirofumi Toda

**Affiliations:** 1 Institute for Genetic Medicine, Hokkaido University, Sapporo, Japan; 2 International Institute for Integrative Sleep Medicine (WPI-IIIS), Tsukuba Institute for Advanced Research (TIAR), University of Tsukuba, Tsukuba, Ibaraki Japan; 3 Institute of Microbial Chemistry (BIKAKEN), Tokyo, Japan; 4 Cell Biology Center, Institute of Integrated Research, Institute of Science Tokyo, Yokohama, Japan; 5 Graduate School of Life Sciences, Tohoku University, Sendai, Japan; 6 The Advanced Center for Innovations in Next-Generation Medicine (INGEM), Tohoku University, Sendai, Japan; 7 Degree Programs in Pure and Applied Sciences, Graduate School of Science and Technology, University of Tsukuba, Tsukuba, Japan; 8 Research Center for Autonomous Systems Materialogy (ASMat), Institute of Integrated Research, Institute of Science Tokyo, Yokohama, Japan; 9 Department of Biological Sciences, School of Science, The University of Tokyo, Tokyo, Japan; 10 Circadian Clock Project, Tokyo Metropolitan Institute of Medical Science, Tokyo, Japan; 11 Graduate School of Pharmaceutical Sciences, Tohoku University, Sendai, Japan; 12 Department of Biomolecular Science, Faculty of Science, Toho University, Funabashi, Japan; 13 Life Science Center for Survival Dynamics, Tsukuba Advanced Research Alliance (TARA), University of Tsukuba, Tsukuba, Japan; 14 Center for Quantum and Information Life Sciences (QiLS), University of Tsukuba, Tsukuba, Japan; 15 Faculty of Advanced Life Science, Hokkaido University, Sapporo, Japan; 16 Howard Hughes Medical Institute, Chronobiology and Sleep Institute, Perelman School of Medicine, University of Pennsylvania, Philadelphia, PA USA

**Keywords:** Antimicrobial resistance, Intrinsically disordered proteins, Ribosome

## Abstract

Antibiotic resistance is a global health threat requiring antimicrobials with alternative modes of action. Growing evidence indicates that bacteria organize key intracellular processes through biomolecular condensates and other forms of spatial organization, yet whether such spatial organization can be exploited as an antimicrobial target remains unclear. Here, we identify intrinsically disordered antimicrobial peptides, generated from the *Drosophila* sleep-inducing protein Nemuri, that inhibit bacterial growth by targeting ribosomal organization. Mechanistically, these peptides enter bacterial cells without overt membrane disruption and accumulate in ribosome-rich regions, where they bind broadly to RNA and ribosomal surfaces through multivalent interactions. This engagement remodels ribosome-rich regions into condensates with reduced ribosomal mobility, thereby suppressing global protein synthesis and distinguishing these peptides from canonical site-specific ribosome-targeting antibiotics. Furthermore, Nemuri-derived peptides exhibit broad-spectrum antimicrobial activity, including activity against drug-resistant pathogens, with low cytotoxicity toward *Drosophila* and mammalian cells. Together, our findings identify the organization and material dynamics of bacterial ribosomes as a biophysical vulnerability that can be exploited by antimicrobial peptides, revealing how a naturally evolved metazoan host-defense system turns intracellular organization itself into an antibacterial target.

## Abstract (web summary)

Nishinami et al. show that peptides from the sleep-inducing protein Nemuri, condense bacterial ribosomes and inhibit protein synthesis, revealing a biophysical antimicrobial strategy targeting intracellular organization.

## Introduction

The global rise of antibiotic-resistant bacteria represents an urgent public health problem, requiring the development of antimicrobials that function via alternative mechanisms. Antimicrobial peptides (AMPs) are produced by diverse organisms and represent a promising avenue to combat the growing threat of antibiotic-resistant bacterial infections^1–4^. These peptides play crucial roles in the innate immune system by protecting hosts from infection. Although many AMPs are thought to act by disrupting bacterial membranes, leading to cell lysis, a growing class of AMPs can enter bacterial cells without disrupting membranes and target intracellular processes^5^. Emerging evidence suggests that such intracellularly acting AMPs can influence key cellular processes such as transcription, translation, protein folding, and cell wall synthesis^1,6–12^.

Recent studies have suggested that bacteria^13–16^, much like eukaryotes^17–28^, can organize intracellular processes within biomolecular condensates and other forms of spatial organization, and that in some cases phase separation underlies the formation of such structures^17,29,30^. In *Escherichia coli*, the best-established example of intracellular organization relevant to translation is the spatial demixing of the nucleoid and ribosomes, whereby ribosomes and polysomes are enriched in DNA-poor regions outside the nucleoid^31,32^. This entropy-driven spatial segregation is shaped by the nucleoid-to-cytoplasm ratio, the DNA meshwork, and the resulting biophysical properties of the bacterial cytoplasm, which together regulate ribosome mobility and localization^33–36^. Beyond nucleoid–ribosome demixing, bacterial mesoscale organization also includes protein-based polar subdomains and glycogen-rich phase-separated assemblies, illustrating that bacterial cytoplasm can be organized through multiple physical principles^37,38^. In parallel, cationic proteins and peptides have been shown to bind ribosomes in the bacterial cytoplasm, slow their diffusion, drive ribosome coacervation or condensation, and inhibit translation^39–42^. Thus, ribosome-rich regions of the bacterial cytoplasm represent biophysically organized and potentially druggable intracellular spaces.

Previously, we identified Nemuri (Nur), a *Drosophila* sleep-inducing protein with dual functions in promoting sleep and killing bacteria in response to stresses such as bacterial infection^43^. However, the molecular basis of Nur-mediated antibacterial activity has remained unclear. Intriguingly, Nur contains an arginine- and glycine-rich (R/G-rich) intrinsically disordered region at its C-terminus, a feature commonly associated with RNA-binding and condensate-related proteins^44^. Here, we show that, unlike canonical AMPs that disrupt bacterial membranes or act on defined intracellular targets through site-specific interactions, Nur-derived intrinsically disordered peptides enter bacterial cells without overt membrane disruption, bind broadly to RNA and ribosomal surfaces, and inhibit translation by modulating ribosome biophysics.

## Results

### R/G-rich short C-terminal peptides of Nur are essential for AMP activity

**Fig. 1:**
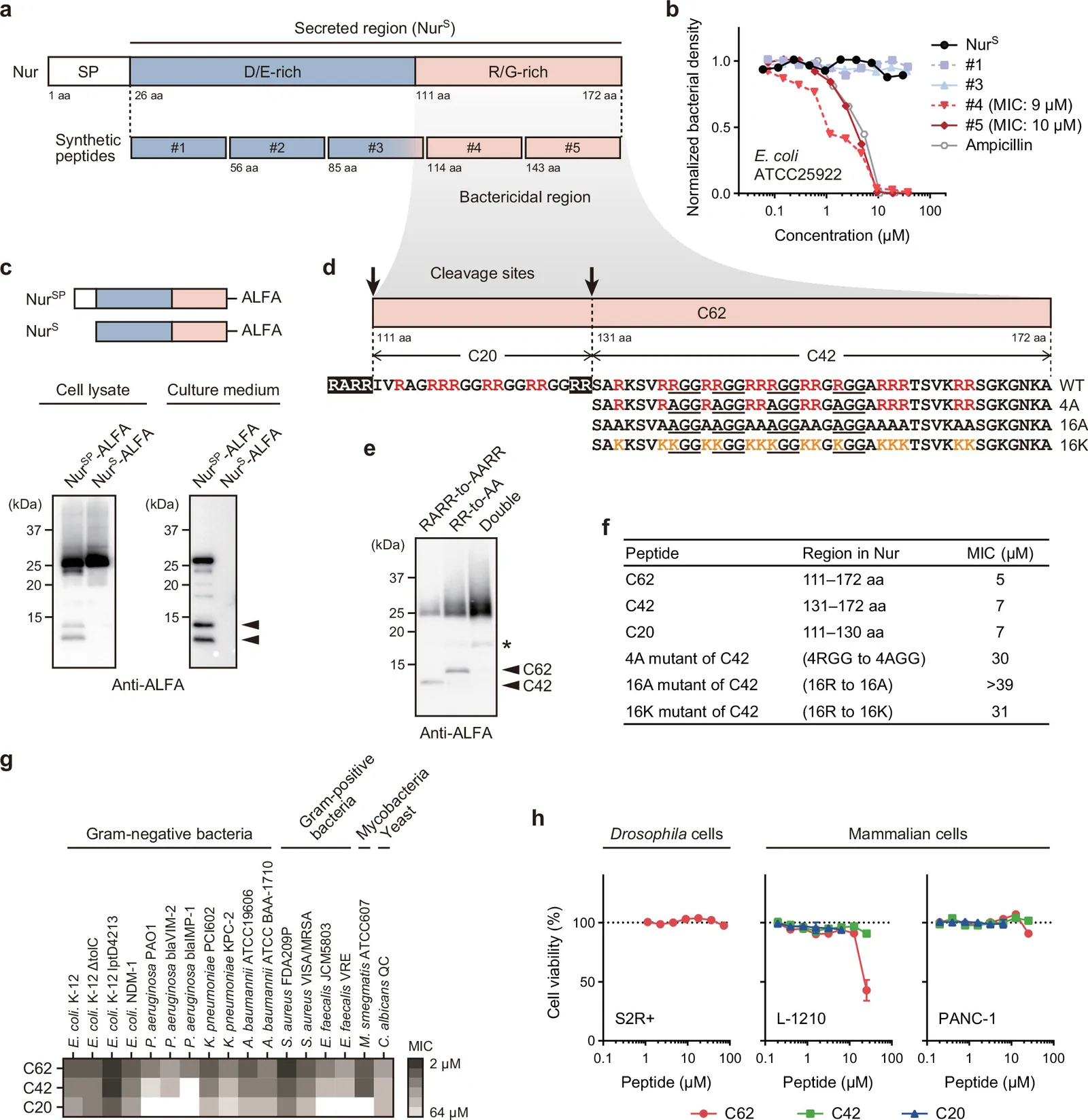
Nur is post-translationally processed into R/G-rich antimicrobial peptides. **a** Schematics of Nur domains showing the signal peptide (SP, 1–25 aa), D/E-rich region, and R/G-rich region. The mature secreted form lacking the SP is termed Nur^S^ (26–172 aa). Nur^S^-based synthetic peptides (#1, 27–55 aa; #2, 56–84 aa; #3, 85–113 aa; #4, 114–142 aa; #5, 143–171 aa) were designed for activity testing. **b** Antimicrobial activity of Nur^S^ and peptides #1, #3, #4, and #5 against *E. coli* ATCC25922. Bacterial density (OD_600_) was normalized to the no-compound control, set to 1. Peptide #2 was not synthesized. Minimum inhibitory concentrations (MICs) are indicated for active peptides #4 and #5. Ampicillin served as a positive control. **c** Western blots of cell lysates and culture medium from S2 cells expressing C-terminally ALFA-tagged Nur with its SP (Nur^SP^) or Nur^S^. Arrowheads indicate cleaved products. **d** Schematics of endogenous Nur processing, with arrows indicating cleavage sites following a furin motif (RARR, 107–110 aa) and a basic dipeptide motif (RR, 129–130 aa). **e** Western blot of S2 cell lysates expressing C-terminally ALFA-tagged, non-cleavable Nur mutants. The RARR and RR sites shaded in (**d**) were replaced with AARR and AA, respectively; the double-site mutant carries both substitutions. Arrowheads and the asterisk indicate cleaved products and a nonspecific band, respectively. **f** MICs of Nur peptides (C62, C42, and C20) and C42 arginine-substitution mutants (4 A, 16 A, and 16 K) against *E. coli* ATCC25922. **g** Antimicrobial spectrum of C62, C42, and C20 against diverse microbes, including drug-resistant strains. Darker shades indicate lower MICs; white indicates MICs > 64 µM. **h** Eukaryotic cytotoxicity of C62, C42, and C20 was evaluated against *Drosophila* S2R+ cells (left), murine L-1210 cells (middle), and human PANC-1 cells (right). Cell viability was measured by MTS assay for S2R+ cells and by MTT assay for L-1210 and PANC-1 cells. Data are shown as mean ± SEM (*n* = 3 technical replicates per condition). Experiments in (**c**, **e**) were each performed independently at least twice with similar results. Source data are provided as a Source Data file.

Nur is a secretory protein with antimicrobial activity^43^. Secreted Nur (Nur^S^) consists of a negatively charged D/E-rich region (26–110 aa) and a positively charged R/G-rich region (111–172 aa) (Fig. 1a). To identify the regions of Nur^S^ particularly important for antimicrobial activity, we synthesized a series of short peptides based on Nur^S^ (Fig. 1a) and performed minimum inhibitory concentration (MIC) assays together with full-length Nur^S^ (Fig. 1b and Suppl. Fig. 1a). Strikingly, the R/G-rich peptides showed significant antimicrobial activity at 9–10 µM, comparable to ampicillin and other AMPs^45^, while the D/E-rich peptides were inactive. Full-length Nur^S^ showed no activity up to 31 µM. These results indicate that the C-terminal R/G-rich region of Nur is critical for its antimicrobial activity, whereas the N-terminal D/E-rich region exerts an inhibitory effect, suggesting that removal of this inhibitory N-terminal region is a prerequisite for Nur to exhibit robust antimicrobial activity.

We thus examined whether the inhibitory N-terminal region is cleaved in *Drosophila* S2 cells stably expressing Nur with its signal peptide (Nur^SP^). As a control, we used cells expressing the mature secreted form, Nur^S^, which lacks the signal peptide. Both constructs were tagged with an ALFA epitope tag at their C-terminus^46^ and were analyzed by Western blotting (Fig. 1c). A high molecular weight product (approximately 26 kDa), presumably corresponding to Nur^S^, was detected in lysates from cells expressing either Nur^SP^ or Nur^S^. However, two additional lower bands were observed only in Nur^SP^ lysates, and these smaller bands also appeared in the culture medium. These findings suggest that Nur is post-translationally processed into shorter peptides and secreted, a process dependent on the presence of its signal peptide and likely occurring via the canonical ER-Golgi secretion pathway^47^.

To identify the cleavage sites, we purified each of the two shorter peptides for protein sequencing. One product corresponded to a C-terminal 42 aa fragment (C42; Suppl. Fig. 1b). The second site was not assigned directly; we therefore applied in silico cleavage predictions^48,49^. Three potential cleavage sites emerged (Fig. 1d): the first at the signal sequence, confirmed by protein sequencing (Suppl. Fig. 1c); the second within the R/G-rich region, generating C42 (Suppl. Fig. 1b); and the third, also within the R/G-rich region, predicted to yield a C-terminal 62 aa peptide (C62). As the recognition motif (RXXR) for a furin-like protease^50^ is present immediately at the N-terminal side of C62, we replaced its first arginine with alanine (Nur^AARR^) and confirmed that the mutation markedly reduced formation of the higher band without affecting the lower band (Fig. 1e). Similarly, mutating the site for C42 (RR to AA, Nur^AA^) eliminated the lower band, and the double-site mutant (Nur^AARR-AA^) prevented the production of both bands (Fig. 1e). These cleavage patterns, yielding C62 and C42, were further confirmed in the bodies and neurons of flies (Suppl. Fig. 1d–f). We also generated a C20 peptide (111–130 aa) because proteolytic processing within the C42/C62 region could potentially release this internal fragment, which would lack the ALFA epitope and thus would not be captured by our ALFA tag assay. Together, these findings demonstrate that Nur has two processing sites in the C-terminal region, generating R/G-rich peptides C62, C42, and presumably C20.

We evaluated the antimicrobial activities of C62, C42, and C20 by MIC assays (Fig. 1f). All three peptides inhibited bacterial growth at 5–7 µM. Since these peptides contain arginine repeats, including RG/RGG motifs, we introduced alanine mutations into C42, targeting either four RGG motifs (C42^4A^) or all 16 arginine residues (C42^16A^). C42^4A^ displayed reduced activity (MIC: 30 µM), whereas C42^16A^ was inactive. The charge-conservative arginine-to-lysine mutant C42^16K^ also showed reduced activity (MIC: 31 µM), suggesting that the guanidinium group of arginine contributes to antimicrobial activity beyond positive charge alone^51^. Thus, arginine repeats are indispensable for antimicrobial activity, demonstrating that Nur cleavage produces functional R/G-rich AMPs.

### Nur peptides show broad-spectrum antimicrobial activity against drug-resistant pathogens

To evaluate the antimicrobial spectrum of Nur peptides, we conducted MIC assays against a broad range of microbes, including antibiotic-resistant strains. The tested microbes included Gram-negative bacteria (*Escherichia coli*, *Salmonella enteritidis*, *Pseudomonas aeruginosa*, *Klebsiella pneumoniae*, *Acinetobacter baumannii*, and *Serratia marcescens*), Gram-positive bacteria (*Staphylococcus aureus*, *Streptococcus pyogenes*, and *Enterococcus faecalis*), mycobacteria (*Mycobacterium smegmatis*, *M. abscessus*, *M. avium*, and *M. intracellulare*), and the pathogenic yeast *Candida albicans* (Fig. 1g and Suppl. Table 1). C62, C42, and C20 peptides effectively inhibited most of the tested microbes, with MIC values ranging from 2 to 32 µM. Among these peptides, C62 generally exhibited the highest potency on a molar basis. Notably, Nur peptides exhibited activity against clinically relevant antibiotic-resistant strains, including New Delhi metallo-beta-lactamase-1 (NDM-1)-producing *E. coli*, multidrug-resistant *P. aeruginosa* strains (MDRP), Klebsiella pneumoniae carbapenemase-2 (KPC-2)-producing *K. pneumoniae*, vancomycin-intermediate/methicillin-resistant *S. aureus* (VISA/MRSA), and vancomycin-resistant *E. faecalis* (VRE)^52–54^. These results demonstrate that Nur peptides possess broad-spectrum antimicrobial activity, including efficacy against clinically important antibiotic-resistant pathogens.

To determine whether this growth inhibition reflected bactericidal activity, we performed minimum bactericidal concentration (MBC) assays for C62, C42, and C20 (Suppl. Table 2). The MBC values were nearly identical to their MIC values, indicating that these peptides exert bactericidal, rather than bacteriostatic, activity at concentrations close to the MIC.

We next evaluated the cytotoxicity of Nur peptides toward eukaryotic cells using MTS/MTT assays (Fig. 1h). C42 and C20 showed no detectable cytotoxicity toward *Drosophila* S2R+ cells or mammalian L-1210 and PANC-1 cells under the tested conditions. C62, the most potent antimicrobial peptide, also showed low cytotoxicity overall, although a modest reduction in viability was observed in L-1210 cells at the highest concentrations. We therefore focused primarily on C42 in subsequent experiments, while using C62 and C20 as comparisons where appropriate. Together, these results indicate that Nur peptides combine broad-spectrum antimicrobial activity, bactericidal activity at concentrations close to the MIC, and low cytotoxicity toward eukaryotic cells under the tested conditions.

### Nur peptides enter bacterial cells without membrane disruption

**Fig. 2:**
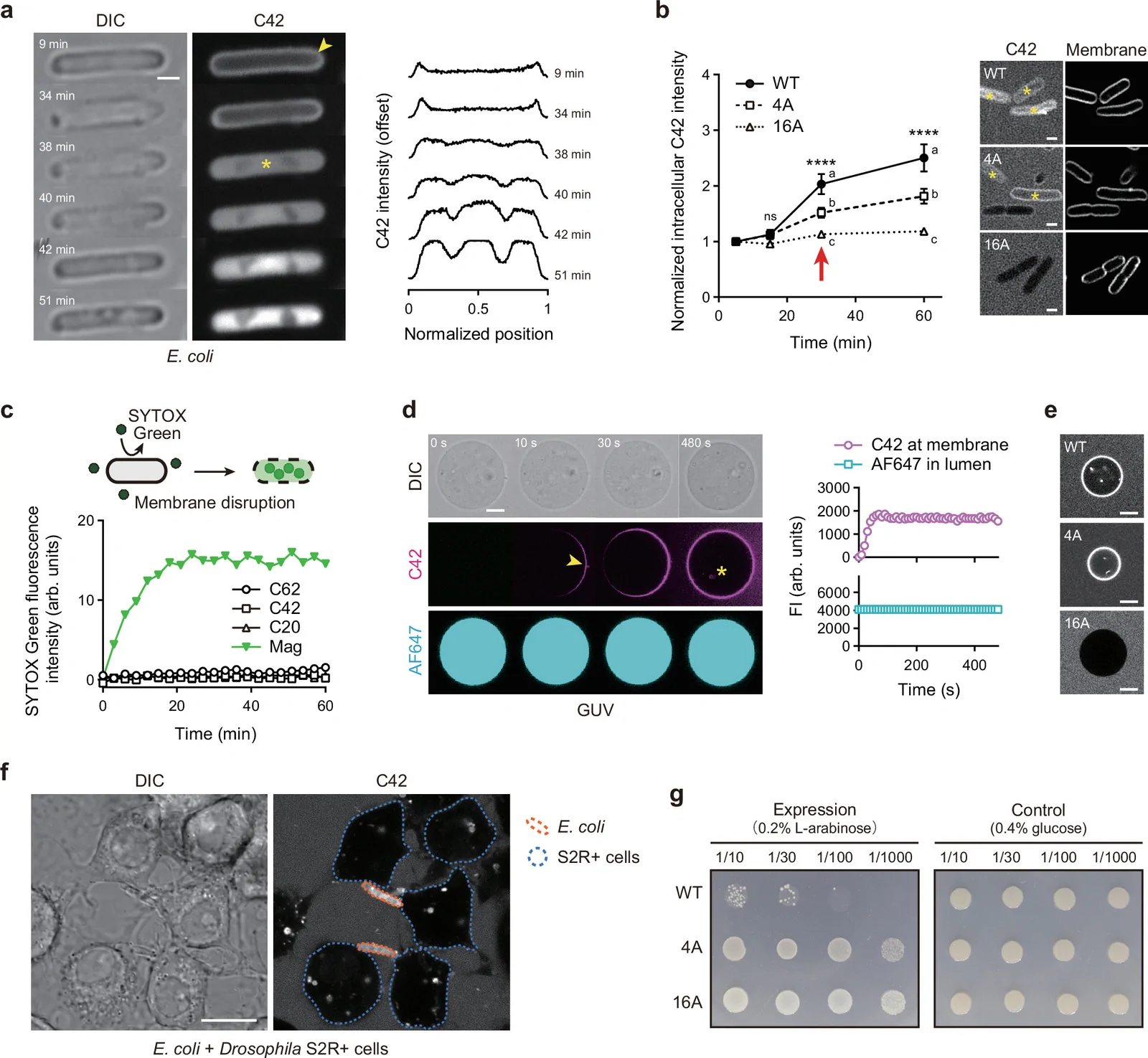
Nur peptides enter bacterial cells without membrane disruption. **a** Time-lapse images of *E. coli* ATCC25922 incubated with C42-FITC in MHB II medium. Times follow peptide addition. Arrowhead and asterisk indicate membrane-associated and intracellular C42-FITC, respectively. The graph shows C42-FITC fluorescence intensity profiles along normalized cell length, vertically offset for clarity. Scale bar, 1 µm. **b** Intracellular accumulation of C42-FITC variants (WT, 4 A, or 16 A) was quantified in log-phase *E. coli* JCM5491 with SynaptoRed-stained membranes at 5, 15, 30, or 60 min after peptide addition. Values were normalized to each variant’s 5-min mean, set to 1. Data are shown as mean ± SEM (*n* = 69, 72, and 70 cells for WT, 4 A, and 16 A, respectively, pooled from two independent trials). At 15, 30, and 60 min, differences were assessed using one-way ANOVA followed by Tukey’s multiple-comparisons test. Overall ANOVA results are shown above the corresponding time points (****P < 0.0001, ns: *P* ≥ 0.05). Different lowercase letters denote significant pairwise differences within each time point (Tukey-adjusted *P* < 0.05). Representative 30-min super-resolution images (red arrow) are shown. Asterisks indicate intracellular peptide localization. Scale bars, 1 µm. **c** SYTOX Green membrane-permeability assay in *E. coli* ATCC25922 treated with C62 (18 µM), C42 (26 µM), C20 (56 µM), or magainin 2 F12W (Mag; 20 µM). **d** Time-lapse images of a DOPG/DOPC (40:60 molar ratio) giant unilamellar vesicle (GUV) encapsulating Alexa Fluor 647 (AF647; cyan) after C42-FITC (magenta) addition. Arrowhead and asterisk indicate membrane-associated and luminal C42-FITC, respectively. The graphs show fluorescence intensity (FI) of membrane-associated C42-FITC and luminal AF647. Scale bar, 10 µm. **e** GUVs treated with C42-FITC (WT, 4 A, or 16 A). Scale bars, 10 µm. **f** *Drosophila* S2R+ cells (blue outlines) co-cultured for 1 h with log-phase L1-msfGFP-expressing *E. coli* CJW7020 (orange outlines) following C42-TAMRA addition. Scale bar, 10 µm. **g** Spot assays of *E. coli* expressing pBAD-driven C42 (WT, 4 A, or 16 A) under 0.2% L-arabinose induction (left) or 0.4% glucose repression (right). Experiments in (**d–f**) were each performed independently at least twice with similar results. Source data are provided as a Source Data file.

Given the potent and broad-spectrum activity of Nur peptides, we next investigated their antimicrobial mechanism. We first visualized peptide localization to bacterial cells using fluorescence microscopy. Among the active Nur peptides, C42 was selected for fluorescent labeling with FITC (C42-FITC) due to its sufficient antimicrobial activity and minimal cytotoxicity (Fig. 1f–h). C42-FITC initially localized to the *E. coli* membrane within 10 min and gradually accumulated inside cells after 30 min (Fig. 2a and Suppl. Fig. 2a). Quantification using Alexa Fluor 488-labeled C42 (C42-AF488) further indicated that treatment with 36 µM peptide, a concentration near the MIC/MBC range, resulted in at least 20-fold intracellular enrichment (850 µM; Suppl. Fig. 2b, c). This intracellular accumulation correlated with its antimicrobial potency: under various culture conditions, antimicrobial activity was consistently observed only when C42 accumulated inside cells (Suppl. Fig. 2d, e). Similarly, the C42^4A^ and C42^16K^ mutants showed reduced internalization and activity, while C42^16A^ exhibited no antimicrobial activity (Fig. 2b and Suppl. Fig. 3a). Furthermore, membrane staining with SynaptoRed^55^ was not noticeably altered by C42 treatment (Fig. 2b), suggesting a cell-penetrating mechanism without membrane disruption. This non-membranolytic mechanism was further supported using SYTOX Green^56^, which fluoresces upon entering membrane-compromised cells (Fig. 2c). Magainin 2 F12W (Mag)^57^, a known membrane-lytic AMP, strongly increased SYTOX Green fluorescence, whereas C62, C42, and C20 did not induce detectable SYTOX Green uptake even when tested at concentrations at least three times their respective MICs. Likewise, Nur peptides did not induce hemolysis of mammalian red blood cells (Suppl. Fig. 3b). These results suggest that Nur peptides act as cell-penetrating molecules that preserve membrane integrity while targeting intracellular processes to mediate antimicrobial activity.

To examine the cell-penetrating mechanism, we employed a giant unilamellar vesicle (GUV) assay^58^ using membrane lipids lacking membrane proteins. A 60 mol% DOPC/40 mol% DOPG lipid mixture was used as a simplified anionic bacterial-like membrane model. C42-FITC rapidly accumulated on the GUV surface within 10 s and was detected inside vesicles within 200 s, whereas encapsulated Alexa Fluor 647 remained confined within GUVs, indicating that C42 crosses membranes without causing noticeable disruption (Fig. 2d). In contrast, the C42^16A^ mutant did not bind to the membrane (Fig. 2e). These results suggest that Nur peptides enter bacterial cells via a direct membrane penetration mechanism. We also tested whether a transporter protein contributes to Nur peptide uptake using an *E. coli sbmA*-deficient mutant, which is resistant to certain intracellular-targeting AMPs^59^. C42 retained its activity against the mutant (Suppl. Fig. 3c), further supporting a transporter-independent penetration mechanism.

Next, to examine whether Nur peptides preferentially enter bacteria over eukaryotic cells, we performed co-culture experiments. C42 readily entered bacterial cells at the MIC/MBC for *E. coli*, whereas uptake into S2R+ cells was minimal and appeared as punctate structures consistent with endocytosis (Fig. 2f). Substantial internalization into S2R+ cells occurred only at much higher concentrations, where it appeared to involve rapid direct penetration (Suppl. Fig. 3d). Taken together, these results suggest that Nur peptides preferentially enter bacterial cells at antimicrobial concentrations through a non-membranolytic mechanism, which may contribute to their selective toxicity toward bacteria.

### Nur peptides target ribosome-rich regions through multivalent interactions

Because AMPs acting intracellularly should exert antimicrobial activity when expressed inside bacteria, we next generated *E. coli* strains expressing Nur peptides under the control of the arabinose-inducible pBAD promoter^59,60^ and performed spot assays. In this system, bacterial growth is observed in glucose medium, but not in arabinose medium if the peptide is antibacterial. C42 expression significantly inhibited bacterial growth on arabinose, whereas C42^4A^ and C42^16A^ did not, and C42^16K^ showed weaker inhibition (Fig. 2g and Suppl. Fig. 4a–c). To dissect the contribution of individual arginine residues, we systematically introduced a series of R-to-A substitutions into the four RGG motifs (AAAR, AARA, AARR, RRAA, ARRR, RARR, RRAR, and RRRA; Suppl. Fig. 4d). Among the four arginine residues within the RGG motifs analyzed, substitution at the third position, located within the central arginine cluster, most severely impaired growth inhibition. These findings indicate that both the number and positional arrangement of arginine residues are critical for the intracellular antimicrobial activity of Nur peptides.

**Fig. 3:**
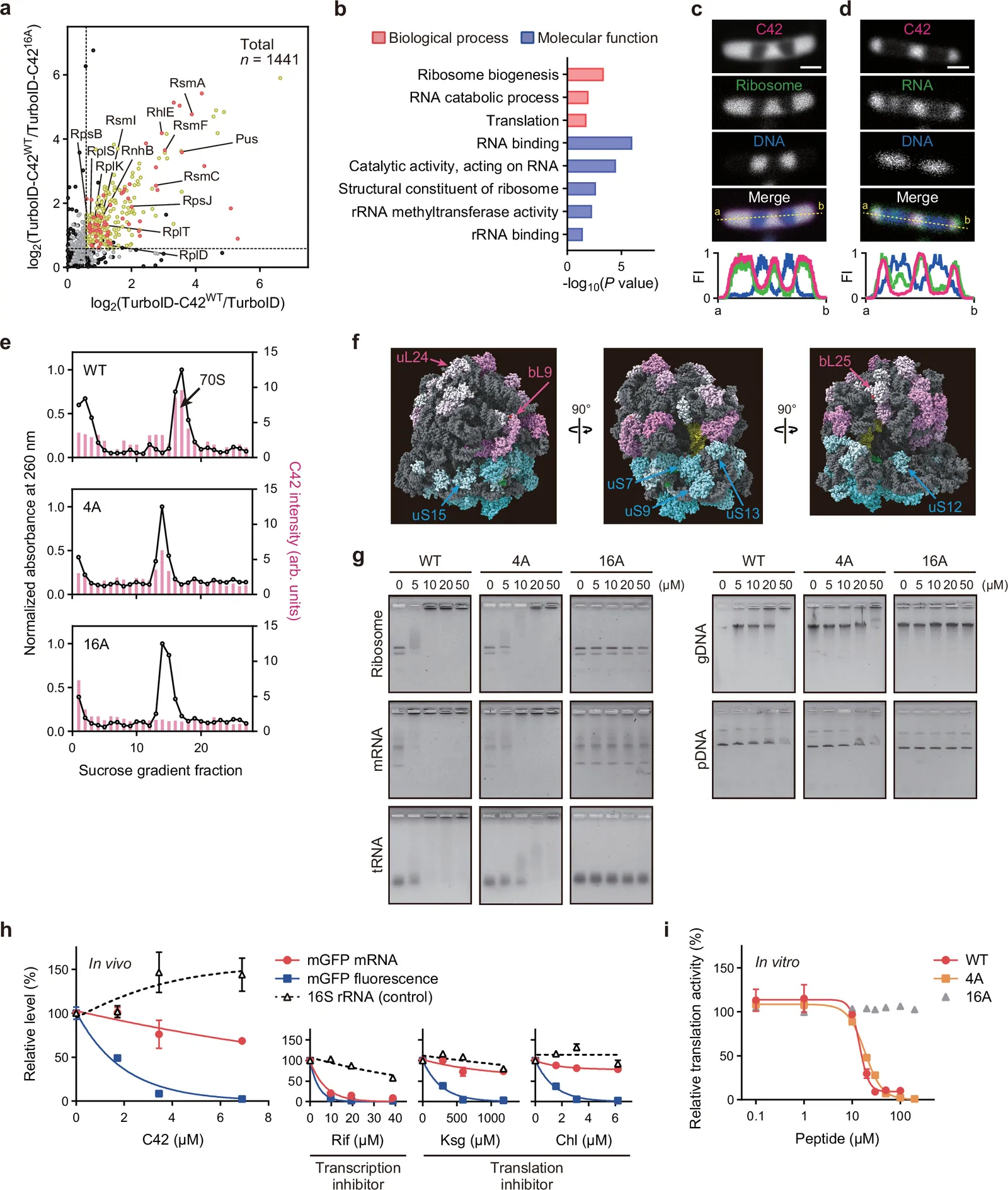
Nur peptides target RNA- and ribosome-rich regions and inhibit translation. **a** Proximity labeling in *E. coli* expressing TurboID-C42 variants (WT or 16 A) or TurboID alone. WT enrichment is shown versus TurboID alone (*x*-axis) and 16 A (*y*-axis). Nominal *P* values were calculated by two-sided unpaired equal-variance Student’s t-tests (*n* = 3 independent experiments per condition). Dots were classified using the 5-h data: black, *P* ≥ 0.5 in at least one comparison; gray, *P* < 0.5 in both but <1.5-fold enrichment in at least one; yellow, *P* < 0.5 and >1.5-fold enrichment in both; red, yellow criteria plus mean scaled WT abundance >150 at 5 and 24 h. **b** Gene Ontology enrichment of 58 candidates from **a** using DAVID. **c**, **d** C42 localization in *E. coli*. L1-msfGFP-expressing cells (ribosomes, green) incubated with C42-TAMRA (magenta) and stained with DAPI (DNA, blue) (**c**); fixed cells expressing mGFP-C42 (magenta) stained with Nucleolus Bright Red (RNA, green) and Hoechst (DNA, blue) (**d**). Graphs show normalized fluorescence along yellow dotted lines from point a to b. Scale bars, 1 µm. **e** Sucrose-gradient profiles of *E. coli* JCM5491 lysates with C42-FITC (WT, 4 A, or 16 A). Normalized 260-nm absorbance (black) and C42-FITC fluorescence (magenta) are shown. Arrow indicates the 70S ribosomal fraction. **f** C42-proximal sites from cross-linking mass spectrometry mapped onto the *E. coli* 70S ribosome (PDB ID: 6OFX). Arrows indicate C42-cross-linked ribosomal proteins. Cross-linked lysines (red), small-subunit proteins (cyan), large-subunit proteins (magenta), rRNA (gray), tRNA (yellow), and mRNA (green) are shown. **g** Electrophoretic mobility shift assays of C42 (WT, 4 A, or 16 A) with 70S ribosomes, mRNA, and tRNA (left) and gDNA and pDNA (right). **h** Transcription and translation in mGFP-expressing *E. coli* after pretreatment with C42, rifampicin (Rif), kasugamycin (Ksg), or chloramphenicol (Chl). **i** In vitro translation of sfGFP with C42 (WT, 4 A, or 16 A). IC_50_: 14.3 µM (WT) and 18.4 µM (4 A); 16 A showed no detectable inhibition. Data are mean ± SEM (*n* = 3 (**h**) or 4 (**i**) independent experiments). Experiments in (**c**, **d**, **g**) were independently repeated at least twice with similar results. Source data are provided as a Source Data file.

We next investigated the intracellular targets of Nur peptides. To identify proteins associated with Nur peptides, we performed proximity labeling using TurboID, a biotin ligase^61^. An *E. coli* strain expressing TurboID-tagged C42 (TurboID-C42) under the control of the pBAD promoter was generated. The mutant TurboID-C42^16A^ and TurboID alone served as negative controls. Biotinylated proteins (Suppl. Fig. 5a) were isolated using streptavidin beads and identified by mass spectrometry. Of the 1441 proteins identified, 58 were enriched in the TurboID-C42 samples compared with the negative controls, including the RNA helicases RhlE and SrmB, RNA polymerase, ribosomal RNA methyltransferases (RsmA, RsmF, and RsmI), ribonucleases, and ribosomal proteins (RpsJ and RplT) (Fig. 3a and Suppl. Data 1). Gene Ontology analysis revealed enrichment of RNA-binding and ribosome-related processes (Fig. 3b). To validate these associations, we examined whether C42 localizes with ribosomal signals in *E. coli* strain CJW7020, which expresses msfGFP-tagged ribosomal protein L1 (L1-msfGFP). L1-msfGFP showed a patchy, non-uniform intracellular distribution, reflecting the spatial organization of ribosome-rich regions, as reported previously^31,32^. RsmI, one of the TurboID-enriched proteins, also showed a non-uniform distribution (Suppl. Fig. 5b). Notably, TAMRA-labeled C42 (C42-TAMRA) strongly co-localized with L1-msfGFP signals when incubated with *E. coli* (Fig. 3c), supporting an association of Nur peptides with ribosome-rich regions. In contrast, Nur peptides retained antimicrobial activity in an RNase E C-terminal truncation strain, in which RNase E-dependent bacterial RNA body formation is expected to be impaired, suggesting that the RNase E C-terminal region is not essential for Nur peptide susceptibility (Suppl. Fig. 5c). Because rRNA is the dominant RNA species in *E. coli*, accounting for up to approximately 90% of total RNA^62,63^, RNA signals are expected to largely overlap with ribosome-rich regions. Indeed, mGFP-C42 expressed in *E. coli* cells co-localized with RNA-specific dyes (Fig. 3d). This association of Nur peptides with RNA was also observed in eukaryotic cells (Suppl. Fig. 5d–g). These results suggest that Nur peptides associate with RNA- and ribosome-rich regions.

To validate these interactions biochemically, we first performed sucrose density gradient centrifugation. C42-FITC was mixed with *E. coli* lysates and subjected to ultracentrifugation in a 10–40% (w/v) sucrose gradient (Fig. 3e and Suppl. Fig. 5h). C42-FITC was detected in the 30S, 50S, and 70S ribosomal fractions, whereas the C42^4A^ mutant showed reduced signals and C42^16A^ showed no detectable signal. These results demonstrate that Nur peptides interact with ribosomes via an arginine-dependent mechanism. To further examine how Nur peptides engage ribosomes, we identified ribosomal protein-proximal contact points compatible with broad ribosome-surface engagement using cross-linking mass spectrometry (XL-MS) (Fig. 3f and Suppl. Data 2). Unlike many ribosome-targeting peptides or antibiotics that bind defined functional sites, such as the polypeptide exit tunnel, C42 showed broadly distributed contacts across the ribosomal surface, with some preference for functionally important regions such as the tRNA transit path (particularly uS9 and uS12). These results suggest that Nur peptides do not bind to a single highly specific ribosomal site, but rather engage in multivalent interactions over broad regions of the ribosome with loose selectivity. We next conducted electrophoretic mobility shift assays (EMSA) and found that C42 bound strongly to various classes of RNA, including rRNA, mRNA, and tRNA, but only weakly to DNA substrates, including genomic and plasmid DNA, in an arginine-dependent manner (Fig. 3g). These results indicate that Nur peptides directly bind to a diverse range of nucleic acids with a preference for RNA over DNA.

### Nur peptides suppress global bacterial protein synthesis

Inhibition of translation is a major mechanism of action for many antibiotics^64,65^. We speculated that Nur peptides inhibit protein synthesis via their ribosome-binding properties. To test this in vivo, we expressed mGFP under the control of the pBAD promoter in *E. coli* and assessed mRNA levels by reverse-transcription quantitative PCR (RT-qPCR) and protein production by GFP fluorescence following peptide treatment. Rifampicin, kasugamycin, and chloramphenicol served as controls for transcription and translation inhibition (Suppl. Fig. 6a). C42 significantly reduced GFP fluorescence without affecting mRNA levels (Fig. 3h and Suppl. Fig. 6b). Western blotting confirmed a reduction in GFP protein levels (Suppl. Fig. 6c). To determine whether C42 suppresses global protein synthesis in vivo, we next assessed nascent protein synthesis in *E. coli* using two distinct labeling approaches, L-azidohomoalanine (AHA) incorporation and puromycin incorporation. C42 markedly reduced the incorporation of both AHA and puromycin, supporting substantial suppression of global protein synthesis by C42 in *E. coli* (Suppl. Fig. 6d, e).

We further verified translation inhibition in vitro using a cell-free translation system derived from *E. coli*^7^, in which sfGFP mRNA is translated into protein (Fig. 3i and Suppl. Fig. 6f). Dose-response analysis showed that C42 inhibited GFP synthesis in a concentration-dependent manner, with an estimated IC_50_ of 14.3 µM and near-complete inhibition at 20 µM. The C42^4A^ mutant retained partial inhibitory activity, with a modestly higher IC_50_ of 18.4 µM, whereas C42^16A^ showed little inhibitory activity, with an estimated IC_50_ greater than 200 µM. These results show that Nur peptides inhibit bacterial translation in an arginine-dependent manner, suppressing global protein synthesis and contributing to their antibacterial activity.

### Nur peptides are intrinsically disordered and form dynamic assemblies

**Fig. 4:**
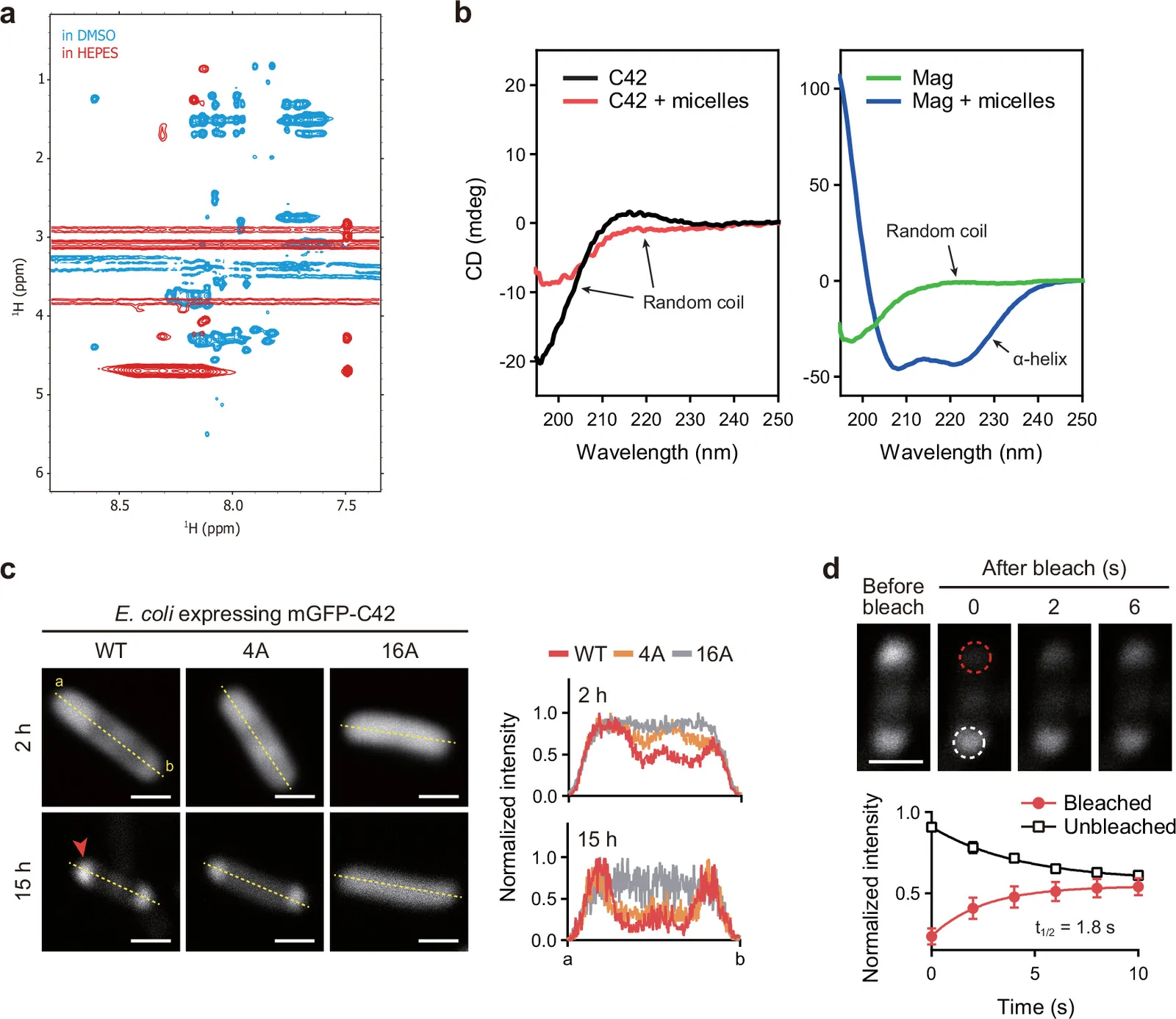
Nur peptides exhibit intrinsic disorder and dynamic assembly. **a** Fingerprint region of 2D [^1^H–^1^H] TOCSY NMR spectra of C42. Spectra in HEPES buffer (red) are overlaid on those in dimethyl sulfoxide (DMSO; cyan). **b** Circular dichroism (CD) spectra of C42 (left) and magainin 2 F12W (Mag; right) in water alone (black and green, respectively) or with sodium dodecyl sulfate (SDS) micelles (red and blue, respectively). **c** Confocal images of *E. coli* expressing mGFP-C42 variants (WT, 4 A, or 16 A) after 2 or 15 h of L-arabinose induction. Arrowhead indicates an mGFP-C42 granule. The graphs show normalized mGFP-C42 fluorescence intensity profiles along the yellow dotted lines from point a to b. WT, 4 A, and 16 A are shown in red, orange, and gray, respectively. Scale bars, 1 µm. The experiment in **c** was performed independently twice with similar results. **d** Fluorescence recovery after photobleaching (FRAP) analysis of *E. coli* expressing mGFP-C42 after 15 h of L-arabinose induction. Red and white dotted circles indicate photobleached and unbleached regions, respectively. The graph shows normalized mGFP-C42 fluorescence in photobleached regions (red circles) and unbleached regions (open squares). Data are shown as mean ± SEM (*n* = 3 cells). The recovery half-time (t_1/2_) was 1.8 s. Scale bar, 1 µm. Source data are provided as a Source Data file.

Membrane-active AMPs typically share common physicochemical and structural features, such as positive charge, hydrophobic residues, and amphipathic structures^6,57,66,67^. In contrast, Nur peptides are rich in arginine and glycine but contain few hydrophobic residues (Fig. 1d). Such RG/RGG motifs are widely associated with intrinsically disordered structures^44^. We therefore assessed disorder propensity using in silico prediction programs^68,69^. These predictions indicated that Nur and its short peptides are intrinsically disordered (Suppl. Fig. 7a). To confirm these predictions, we performed nuclear magnetic resonance (NMR) spectroscopy using C42 as a representative peptide (Fig. 4a and Suppl. Fig. 7b). C42 exhibited distinct NMR signals in DMSO solution, whereas the NMR spectrum in aqueous solution at pH 7 was consistent with the absence of a stable folded structure. To further characterize the biophysical behavior of C42, we analyzed secondary structure by circular dichroism (CD) spectroscopy. SDS micelles were used as a membrane-mimetic environment (Fig. 4b). Upon addition of SDS micelles, Mag, a membrane-active AMP^57^, adopted an amphipathic helical structure, with its α-helical content increasing from 2.4% to 53.7%. In contrast, C42 remained in a random-coil conformation. Collectively, these results suggest that Nur peptides are intrinsically disordered and lack membrane-induced structuring.

Given that intrinsically disordered proteins can contribute to biomolecular condensates and membraneless organelles through multivalent weak interactions^70^, we next examined how these unusual physicochemical properties shape their intracellular behavior. Fluorescence recovery after photobleaching (FRAP) analysis of intracellular C42-FITC in *E. coli* showed rapid fluorescence recovery within C42-enriched regions, with the bleached signal recovering to levels comparable to unbleached regions within 20 s (Suppl. Fig. 7c). This rapid recovery indicates molecular exchange within C42-enriched regions rather than static accumulation. Similarly, prolonged intracellular expression of mGFP-C42 in *E. coli* produced discrete granular structures (Fig. 4c). The C42^4A^ mutant showed reduced assembly formation, whereas C42^16A^ displayed a diffuse cytoplasmic distribution (Fig. 4c). FRAP analysis further showed fluorescence recovery within mGFP-C42 assemblies, consistent with dynamic exchange (Fig. 4d). These results suggest that intrinsically disordered Nur peptides form arginine-dependent dynamic assemblies.

### Nur peptides induce ribosomal condensation

**Fig. 5:**
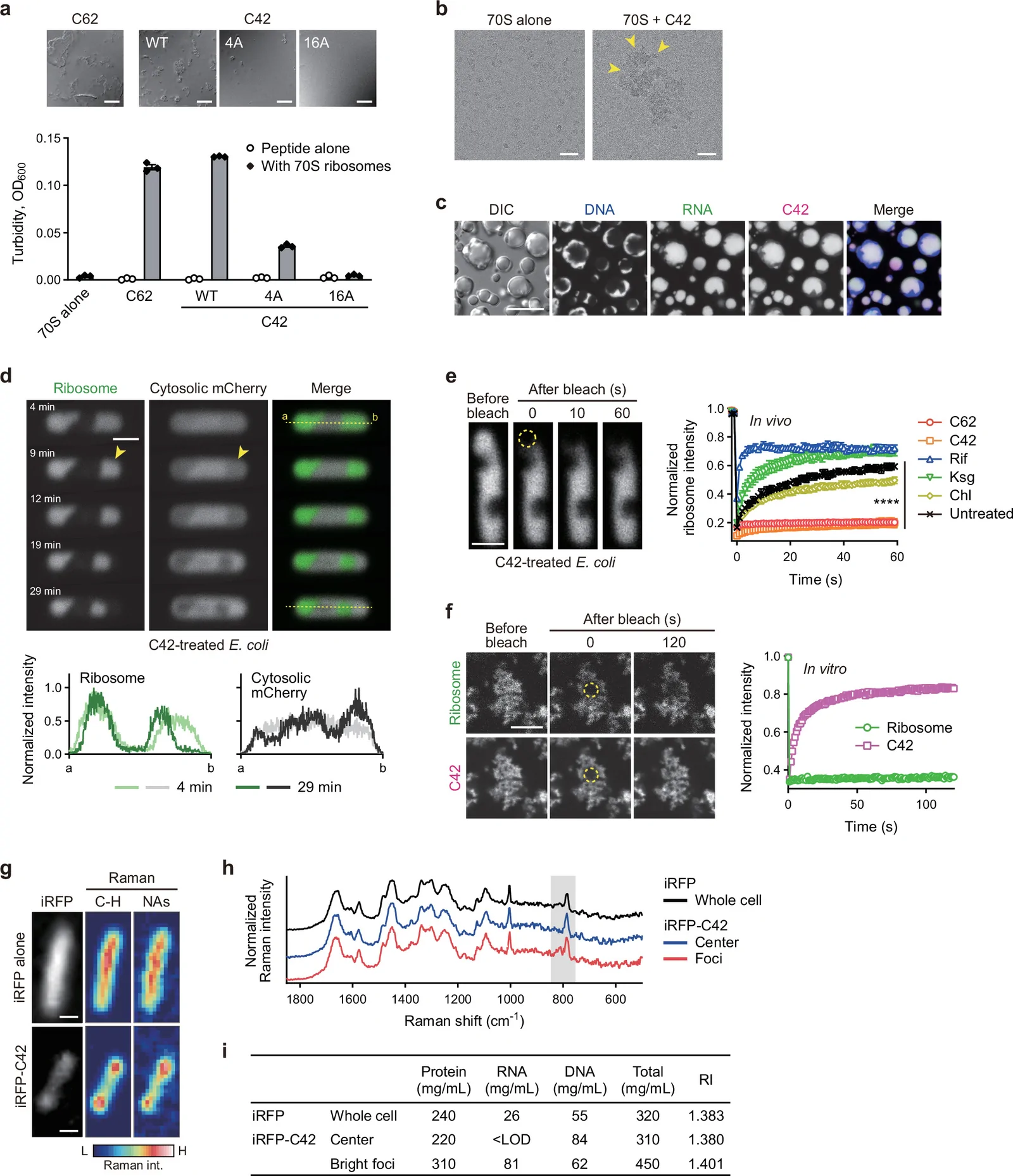
Nur peptides condense ribosomes and reduce their mobility. **a–c** In vitro condensation. **a** Condensation of purified 70S ribosomes (1 µM) by C62 or C42 variants (WT, 4 A, or 16 A; 50 µM) in Polymix buffer. Representative images show assembly states. Turbidity is shown for peptides alone (open circles) and 70S-containing samples (black diamonds), including 70S alone. Bars show mean ± SEM (*n* = 3 technical replicates). Scale bars, 10 µm. **b** Cryo-EM of 70S ribosomes alone or with C42. Arrowheads indicate ribosome assemblies. Scale bars, 50 nm. **c** Multiphase condensates of dsDNA (blue) and ssRNA (green) induced by C42 (100 µM; magenta) in Polymix buffer. Scale bar, 10 µm. **d** Condensation-like remodeling of ribosome-rich regions in *E. coli* CJW7020 expressing L1-msfGFP (ribosomes, green) and cytosolic mCherry (gray) after 20 µM C42 addition. Arrowheads indicate ribosome-enriched, mCherry-depleted regions. Plots show normalized fluorescence along dotted (**a**, **b**) lines. Scale bar, 1 µm. **e** FRAP of L1-msfGFP in *E. coli* treated with C62, C42, rifampicin (Rif), kasugamycin (Ksg), chloramphenicol (Chl), or untreated (*n* = 11, 17, 8, 13, 31, and 10 cells, respectively). Dotted circle indicates photobleaching in the C42-treated cell. Graph shows normalized fluorescence (mean ± SEM). One-way ANOVA with Dunnett’s test compared C42 to untreated cells at endpoint (****: *P* < 0.0001). Scale bar, 1 µm. **f** In vitro FRAP of condensates containing mCherry-tagged 70S ribosomes (green) and C42-AF488 (magenta). Dotted circles indicate photobleaching. Scale bar, 5 µm. **g** Raman and fluorescence images of iRFP- or iRFP-C42-expressing *E. coli*. Maps show C–H stretching (2800–3000 cm^−1^) and pyrimidine signals from nucleic acids (NAs; 787 cm^−1^). Scale bars, 1 µm. **h** Raman spectra averaged over iRFP-expressing cells and foci or centers of iRFP-C42-expressing cells (*n* = 16, 14, and 17 cells, respectively), offset vertically. The shaded 814 cm^−1^ band indicates RNA enrichment in foci versus centers. **i** Raman-derived protein, RNA, and DNA concentrations and total biomolecular concentrations from refractive index (RI). LOD, limit of detection. Experiments in (**b–d**, **f**, **g**) were independently repeated at least twice with similar results. Source data are provided as a Source Data file.

Given these intrinsically disordered and assembly-forming properties and their broad multivalent engagement with RNA and ribosomal surfaces, we next asked whether Nur peptides alter ribosome organization itself. We first examined their effects in a complex bacterial lysate environment. In an *E. coli* lysate system, C42 induced visible condensate-like assemblies in an arginine-dependent manner (Suppl. Fig. 8a). Because this complex system does not establish whether ribosomes themselves are sufficient for this assembly process, we next examined purified 70S ribosomes under defined in vitro conditions (Fig. 5a). In ribosome-compatible Polymix buffer^71^, ribosomes remained largely dispersed in the absence of Nur peptides, whereas incubation with C42 induced visible ribosomal condensates. This effect was reduced for C42^4A^ and abolished for C42^16A^, consistent with the requirement of arginine-rich motifs for antimicrobial activity and translation inhibition. C62 similarly induced ribosomal condensates under the same conditions. Complementary cryo-EM analysis showed dispersed 70S ribosomes in the absence of C42 and clustered ribosomal assemblies after C42 treatment (Fig. 5b). Together, these results indicate that Nur peptides can directly induce ribosomal condensation and clustering in vitro.

We next examined whether Nur peptides preferentially promote the formation of and partition into RNA- and ribosome-containing condensates rather than DNA-containing condensates in vitro. To address this, we analyzed matched-sequence RNA and DNA oligonucleotides^72^ (Suppl. Fig. 8b), as well as ribosomes (Suppl. Fig. 8c), in Polymix buffer. C42 induced nucleic-acid assembly formation with an apparent preference for RNA over DNA (Suppl. Fig. 8d). In mixed DNA/RNA solutions, C42 induced the formation of multiphase condensates in which RNA and DNA were spatially separated, and C42 preferentially partitioned into the RNA-rich phase in a peptide concentration-dependent manner (Fig. 5c). Consistent with these observations, in a simplified poly-L-lysine (PLL)-based multiphase droplet system^72^, C42 also preferentially partitioned into RNA- and ribosome-containing phases rather than DNA-containing phases (Suppl. Fig. 8e, f). Notably, preferential enrichment of C42 in the RNA-rich phase was observed even when matched-sequence single-stranded RNA and DNA were compared, suggesting that RNA-specific physicochemical features, beyond nucleotide sequence and strand structure, contribute to Nur peptide partitioning. Consistent with its weak but detectable DNA interaction observed in EMSA, C42 also inhibited transcription from a DNA template under defined in vitro conditions (Suppl. Fig. 8g), suggesting that Nur peptides can also engage DNA in simplified in vitro systems. Together, these observations suggest that Nur peptides preferentially associate with ribosome-rich regions in vitro, consistent with their localization to ribosome-rich regions in bacteria, likely through RNA-mediated interactions.

We next examined how Nur peptides affect ribosome-rich regions that are spatially segregated from nucleoids in living bacteria (Suppl. Fig. 9a–c). Using an *E. coli* strain expressing the ribosomal marker L1-msfGFP, we found that treatment with C42 progressively sharpened the L1-msfGFP signal, which became concentrated in smaller, brighter ribosome-rich regions (Suppl. Fig. 9d). To determine whether this reflected selective remodeling of ribosome-rich regions rather than a global collapse of the cytoplasm, we co-expressed mCherry as a soluble cytoplasmic marker. In untreated cells, mCherry was broadly distributed throughout the cytoplasm, whereas during Nur peptide-induced remodeling of ribosome-rich regions, mCherry was excluded from the L1-msfGFP-enriched regions (Fig. 5d). Thus, Nur peptides preferentially induce condensation-like remodeling of ribosome-rich regions. Consistent with this, simultaneous imaging of L1-msfGFP, HU-mCherry, and DAPI showed that C42 treatment compacted L1-msfGFP-positive ribosome-rich regions, whereas DAPI-labeled nucleoids remained spatially confined (Suppl. Fig. 9e). HU-mCherry showed partial redistribution outside the DAPI-positive regions, suggesting that ribosome-rich region remodeling may be accompanied by secondary changes in ribosome–nucleoid organization.

### Nur peptides reduce ribosomal mobility

We then examined how Nur peptide-induced condensation-like remodeling of ribosome-rich regions affects ribosomal dynamics by FRAP analysis. In untreated *E. coli*, L1-msfGFP fluorescence recovered within 60 s after photobleaching, indicating that ribosomal components remain dynamic within native ribosome-rich regions (Suppl. Fig. 10a). HU-mCherry-positive nucleoid-associated regions also showed fluorescence recovery, consistent with the dynamic ribosome–nucleoid organization reported previously^14–16,33–36^ (Suppl. Fig. 10b). Treatment with C42 or C62, however, almost completely abolished L1-msfGFP fluorescence recovery, indicating a substantial reduction in ribosomal mobility (Fig. 5e). This mobility reduction was not observed following treatments with canonical transcription or translation inhibitors^65,73,74^: rifampicin and kasugamycin increased L1-msfGFP mobility, whereas chloramphenicol caused only a modest reduction (Fig. 5e and Suppl. Fig. 10c–e). These results indicate that Nur peptides restrict ribosomal dynamics in a manner distinct from conventional transcription or translation inhibition.

We next tested whether Nur peptides directly reduce ribosomal mobility in vitro under the same conditions used in Fig. 5a. To visualize ribosome and peptide dynamics simultaneously, we used purified mCherry-tagged ribosomes together with C42-AF488. FRAP analysis showed that the fluorescence recovery of the ribosomal mCherry signal within C42-induced ribosomal condensates was strongly reduced, indicating reduced ribosomal mobility (Fig. 5f). Interestingly, C42-AF488 remained dynamic within the condensates, as indicated by fluorescence recovery after photobleaching. A similar contrast was observed in living bacteria, where ribosomal mobility was restricted, whereas both C42 and cytoplasmic mGFP retained fluorescence recovery (Suppl. Fig. 10f, g). These in vitro and in vivo observations indicate that Nur peptides selectively reduce ribosomal mobility within ribosome-rich condensates without broadly immobilizing soluble cytoplasmic components.

To further characterize the molecular composition of Nur peptide-induced ribosome-rich condensates in cells, we performed Raman microscopy, a label-free imaging technique^75^, in *E. coli* cells expressing iRFP-tagged C42. By combining Raman microscopy with refractive index imaging, we estimated total biomolecular concentrations as well as DNA, RNA, and protein concentrations (Fig. 5g–i and Suppl. Fig. 10h–j). C42-enriched condensates showed elevated protein concentrations relative to untreated cytoplasm and C42-excluded regions. RNA signals were concentrated within these condensates, whereas DNA showed approximately 1.5-fold enrichment in the C42-excluded regions. These observations support the view that Nur peptide-induced condensates are RNA- and protein-rich, ribosome-associated compartments that remain compositionally distinct from DNA-enriched nucleoid regions.

## Discussion

Traditional antibiotics typically act through high-affinity binding toward defined molecular targets, including discrete binding pockets on the ribosome, often described by a lock-and-key paradigm. In contrast, our results show that intrinsically disordered peptides derived from the *Drosophila* sleep peptide Nur engage in multivalent interactions with diverse RNAs and ribosomal surfaces, a process coupled to condensation-like remodeling of ribosome-rich regions and reduced ribosomal mobility. In actively growing *E. coli*, ribosomes are already spatially segregated from the nucleoid through entropy- and nucleoid-to-cytoplasm ratio-dependent organization of the bacterial cytoplasm^33,34^. Against this biophysical background, Nur treatment further clusters the ribosome-rich population and reduces its mobility, without causing comparable immobilization of freely diffusing cytoplasmic factors. This marked loss of ribosome mobility is accompanied by substantial inhibition of global protein synthesis and antibacterial activity. Importantly, other translational inhibitors including chloramphenicol and kasugamycin suppress translation without inducing the same degree of ribosome immobilization, indicating that this loss of ribosomal mobility is not simply a consequence of translation inhibition. We therefore interpret FRAP data conservatively as a readout of the mobility and exchange of labeled components, rather than as definitive evidence for LLPS or for the material state of an entire assembly. Consistent with current standards in the condensate field, we distinguish Nur-induced condensation-like remodeling and reduced ribosomal mobility from global cytoplasmic gelation, liquid-like behavior of the entire compartment, or pre-existing ribosomal LLPS^76–79^. Accordingly, the principal physical change we detect is a selective reduction in ribosome dynamics. Consistent with this interpretation, Raman and refractive-index analyses indicate localized molecular enrichment in Nur-positive foci rather than a uniform cytoplasmic densification. Thus, Nur appears to inhibit translation by directly modulating the physical behavior of ribosomes, a mode of action that may help explain its broad antimicrobial spectrum and may make Nur peptides less vulnerable to classical target-site resistance mechanisms than conventional ribosome-targeting antibiotics^80^.

Previous studies have established that cationic proteins and peptides can perturb ribosomes and nucleic-acid-rich cellular material through electrostatic and multivalent interactions. Related cationic peptides and proteins have been shown to perturb RNA-rich compartments, inhibit ribosome-associated processes, promote ribosome coacervation or condensation, and cause broader cytoplasmic rigidification, depending on sequence, charge identity, and cellular context^9,39–42,81–83^. In bacteria, positively charged proteins can be trapped by negatively charged ribosomes^39^, cationic AMPs can induce ribosome flocculation/coacervation correlated with killing activity^40^, and LL-37-related cationic peptides can condense ribosomes and DNA through complex coacervation-like mechanisms^41^. Our findings are therefore not only conceptually aligned with these earlier studies, but also extend them in an important biological direction: they show that an endogenous metazoan host-defense system, namely *Drosophila* Nur, appears to exploit this same biophysical vulnerability through proteolytic liberation of intrinsically disordered R/G-rich peptides. In this sense, Nur is not merely another synthetic or pathological polycation, but a naturally evolved antimicrobial principle embedded in animal physiology.

The spatial demixing of nucleoid and ribosomes in bacteria is reminiscent of the hierarchical organization of the eukaryotic nucleolus, a membraneless organelle/biomolecular condensate involved in ribosome biogenesis^84^. In *E. coli*, translation and ribosome biogenesis are thought to occur in relatively close proximity, whereas in eukaryotic cells, these processes are clearly segregated into the cytoplasm and the nucleus, respectively. The ribosome-assembly helicases RhlE and SrmB, which have been reported to form condensate-like assemblies^85^, were enriched in the TurboID-C42 dataset, raising the possibility that Nur peptides may also influence ribosome assembly or related RNA-rich condensates. Nur peptides can penetrate both bacterial and eukaryotic cells, localizing to the nucleolus and cytoplasmic RNA-rich regions. Intriguingly, similar localization patterns have been reported for PR and GR dipeptides in the context of neurodegenerative *C9orf72* repeat expansions, which exert cytotoxicity by disrupting nucleolar condensates depending on their length and sequence composition^81,86,87^. However, the mode of ribosome inhibition appears to differ. These *C9orf72*-derived peptides and certain intracellular-targeting AMPs can directly occupy the ribosomal polypeptide exit tunnel and peptidyl-transferase center, thereby competing with tRNA substrates and inhibiting translation through a structurally defined mechanism^8,42,59^. By contrast, Nur peptides interact with various RNAs to a similar extent and bind to widespread sites across the ribosome, suggesting a broader, mesoscale mode of inhibition that couples multivalent RNA/ribosome engagement to condensation-like remodeling and reduced ribosome mobility. Understanding why the Nur system generates a suite of peptides (C62, C42, and the putative C20) will be particularly informative, as this multi-peptide strategy likely optimizes the window between biological potency and pharmacological safety. Therefore, elucidating how Nur achieves target specificity, remodels ribosome-rich assemblies, and maintains low toxicity to host eukaryotic cells, including its as-yet-unknown membrane-translocation mechanism, will be important to clarify in future investigations. As increasing evidence suggests the existence of various bacterial condensates and mesoscale assemblies^13–16^, it would be interesting to see whether these structures, or artificially engineered counterparts, can also be pharmacologically manipulated.

Our findings also raise the possibility that the previously reported sleep-promoting function of Nur^43^ may involve regulation of RNA–protein assemblies, although this remains to be tested. In neurons, stress-induced Nur might locally modulate ribosomal and/or mRNA dynamics through reversible remodeling of RNA–protein assemblies, thereby altering local protein synthesis. Such regulation could contribute to changes in the neuronal proteostatic environment associated with sleep and stress responses, providing a potential link between the antimicrobial and sleep-related functions of Nur.

## Methods

### Fly maintenance and transgenic line generation

Flies (*Drosophila melanogaster*) were reared on standard cornmeal-based fly food composed of 40 g agar, 200 g yeast, 500 g glucose, 450 g cornmeal, 25 mL propionic acid, 2.5 g methyl benzoate, 25 mL ethanol, and 5.7 L water. They were maintained at 25 °C with a 12:12 light–dark cycle. The UAS-Nur-ALFA transgenic fly line was generated at the attP2 landing site using the plasmid listed in Suppl. Table 3. Embryo microinjections were performed by Rainbow Transgenic Inc.

### Eukaryotic cell culture and transfection

*Drosophila* S2 and S2R+ cells were maintained at 25 °C in 2 mL of Schneider’s *Drosophila* medium (Gibco, 21720001) supplemented with 10% heat-inactivated fetal bovine serum (FBS; Life Science Production, S-001A-BR) and penicillin-streptomycin (FUJIFILM Wako, 168-23191) in 60-mm dishes (BM Bio, 93060). For transfection, 5–10 µg of plasmid DNA was mixed with a 4-fold volume of HilyMax solution (Dojindo, H357) in 300 µL of serum-free medium and added to cells. To generate stable cell lines, transfected cells were selected with 0.5 mg/mL hygromycin B (FUJIFILM Wako, 084-07681) for 2 weeks.

Human U2OS cells were cultured at 37 °C in 5% CO_2_ in Dulbecco’s Modified Eagle Medium (DMEM; FUJIFILM Wako, 043-30085) supplemented with 10% heat-inactivated FBS (Sigma-Aldrich, 173012) and penicillin-streptomycin (Nacalai Tesque, 09367-34).

Human pancreatic cancer PANC-1 cells were cultured at 37 °C in 5% CO_2_ in Dulbecco’s Modified Eagle Medium (DMEM; Shimadzu, 05919) supplemented with 10% heat-inactivated FBS (Nichirei, 175012) and penicillin-streptomycin (FUJIFILM Wako, 161-23201).

Murine leukemia L-1210 cells were cultured at 37 °C in 5% CO_2_ in RPMI 1640 medium (Shimadzu, 05918) supplemented with 10% heat-inactivated FBS (Nichirei, 175012) and penicillin-streptomycin (FUJIFILM Wako, 161-23201).

### Bacterial strains and growth conditions

*E. coli* DH5α was used for plasmid construction and propagation. *E. coli* BL21(DE3) was used as the host for recombinant protein expression from the pGBHPS vector, TurboID proximity labeling, spot assays, and microscopic imaging using pBAD vectors. For microscopic imaging, *E. coli* strains ATCC25922, CJW7020 expressing L1-msfGFP, JCM5491, an HU-mCherry-expressing strain, and CJW7020 derivatives harboring pBAD-mCherry or pBAD-mCherry-C42 were also used. *E. coli* MRE600 or JCM5491 was used for sucrose density gradient centrifugation. *E. coli* JCM5491 was used for genomic DNA extraction, and *E. coli* QC101 was used for ribosome purification. *E. coli* strains were cultured under the conditions described in each experimental section, with appropriate antibiotics for plasmid maintenance. Protein expression from pGBHPS was induced with IPTG, while expression from pBAD vectors, including TurboID proximity labeling, spot assays, and imaging experiments, was induced with L-arabinose. Biotin was also added to the medium for TurboID experiments. The bacterial, mycobacterial, and yeast strains used for MIC assays are listed in Suppl. Table 1. Specific culture conditions, including medium, temperature, and incubation time, are described in the MIC assays section.

### Generation of an *E. coli* strain expressing L1-msfGFP and HU-mCherry

An *E. coli* strain, CJW7020, expressing L1-msfGFP was transformed with a chloramphenicol-resistant plasmid carrying the p15A origin of replication and three phage lambda genes required for λ Red recombination (*gam*, *bet*, and *exo*), under the control of an arabinose-inducible promoter and its regulator gene *araC*. The resulting transformant was subsequently subjected to electroporation (Bio-Rad Gene Pulser II, 0.2-cm gap cuvette, 25 µF, 200 Ω, 2.5 kV) with genomic DNA from an *E. coli* strain expressing HU-mCherry and a kanamycin resistance gene. Transformants were selected on kanamycin, yielding a single colony. This colony was subcultured, streaked on LB agar plates, and colonies that had lost the plasmid were identified by chloramphenicol sensitivity. The resulting strain was confirmed by PCR and phenotype.

### Plasmid construction

Plasmids used in this study are listed in Suppl. Table 3. DNA fragments were amplified by PCR using either PrimeSTAR Max DNA Polymerase (TaKaRa Bio, R045) for high-purity DNA templates or SapphireAmp Fast PCR Master Mix (TaKaRa Bio, RR350) for crude templates. PCR products were confirmed by agarose gel electrophoresis stained with GreenView nucleic acid stain (Relyon, N100) and purified using FastGene Gel/PCR Extraction Kit (FastGene, FG91302). The DNA fragments were cloned into vectors using the In-Fusion HD Cloning Kit (TaKaRa Bio, 639648). The resulting plasmids were verified by Sanger sequencing (Azenta). For plasmid preparation, QIAprep Spin Miniprep Kit (QIAGEN, 27104) was used for small-scale cultures and NucleoBond Xtra Midi Kit (MACHEREY-NAGEL, 740410) was used for large-scale cultures. Newly generated plasmids are available from the corresponding authors upon request.

### Immunoprecipitation

To identify short peptides produced from Nur, immunoprecipitation was performed using ALFA-tagged constructs (Suppl. Table 3). S2 or S2R+ cells expressing ALFA-tagged Nur and their culture media were collected by centrifugation at 200 × g for 3 min at 4 °C. For fly experiments, progenies from UAS-Nur-ALFA flies crossed with nsyb-Gal4 were collected, and heads or whole bodies were collected. Cell pellets or fly samples were homogenized in RIPA buffer (Nacalai Tesque, 08714-04), and lysates were cleared by centrifugation at 14,000 ×g for 5 min at 4 °C. ALFA-specific beads (NanoTag Biotechnologies; magnetic: N1515, non-magnetic: N1510) were washed three times with resin equilibration buffer (10% IGEPAL CA-630 (NP-40; Sigma-Aldrich, I8896), 1× TBS), mixed with lysates, and incubated at 4 °C for 2 h in the presence of protease inhibitor cocktail (Promega, G6521). Beads were washed five times with wash buffer (0.5% Triton X-100, 400 mM NaCl, 1× TBS) for S2 cell samples, or eight times with strong wash buffer (1.0% Triton X-100, 1 M NaCl, 1× TBS) for fly samples. After washing, the beads were resuspended in 1× SDS sample buffer prepared by diluting 4× Laemmli sample buffer (Bio-Rad, 1610747) supplemented with 2-mercaptoethanol. Samples were heated at 95 °C for 5 min for Western blot analysis.

### Western blotting

Protein samples were separated by 10–15% SDS-PAGE and transferred to polyvinylidene difluoride (PVDF) membranes (Bio-Rad, 1704156) using the Trans-Blot Turbo Transfer System (Bio-Rad, 1704150). Membranes were blocked for 30 min at room temperature using either Blocking One solution (Nacalai Tesque, 03953-95) for avidin-peroxidase detection or 1% skim milk (Morinaga) in TBS-T (1× TBS with 0.1% Tween 20) for anti-ALFA detection. Membranes were then incubated for 1 h at room temperature with avidin-peroxidase (Sigma-Aldrich, A3151, 1:2000) or an HRP-conjugated anti-ALFA antibody (NanoTag Biotechnologies, N1505-HRP, 1:4000) in their respective blocking solutions. After washing with TBS-T, signals were detected using an enhanced chemiluminescence (ECL) reagent (PerkinElmer, NEL120001EA) and visualized with a Fusion Solo S imaging system (Vilber Bio Imaging).

### Protein sequencing

ALFA-tagged Nur peptides purified by immunoprecipitation (see above) were separated by SDS-PAGE and transferred onto PVDF membranes (Bio-Rad, 1704156). The membranes were then stained with a Coomassie Brilliant Blue (CBB) R-250 solution, composed of 0.1 g CBB R-250 (FUJIFILM Wako, 031-17922), 22.5 mL ethanol, 5 mL acetic acid, and 22.5 mL water. After staining, the membranes were washed with 50% ethanol and rinsed with water. Individual protein bands were excised with clean scissors and subjected to N-terminal amino acid sequencing. Sequencing was performed by the Institute for Protein Research, Osaka University.

### Peptide synthesis

C20, C42, and C62 peptides were chemically synthesized and purified by high-performance liquid chromatography (HPLC) with water/acetonitrile gradients containing 0.1% trifluoroacetic acid (TFA). These peptides were obtained at approximately 90% purity. Other peptides were synthesized by GL Biochem (Shanghai) at > 80% purity. Concentrations of unlabeled peptides were determined using the Micro BCA Protein Assay Kit (Thermo Fisher Scientific, 23235). Fluorescently labeled C42 peptides were designated as C42-FITC, C42-AF488, and C42-TAMRA throughout the manuscript. Unless otherwise indicated, C42-FITC and its mutant derivatives contain an additional C-terminal lysine residue labeled with FITC. C42-AF488 contains an additional N-terminal cysteine residue labeled with Alexa Fluor 488, and C42-TAMRA is labeled with 5-TAMRA at the N-terminus. The C4216K-FITC mutant was an exception and contained an N-terminal FITC-Ahx label. The exact sequences and labeling positions are listed in Suppl. Table 4. For FITC-labeled peptides, concentrations were calculated based on absorbance at 490 nm in pH 9.0 buffer, using a molar extinction coefficient of 80,000 M^−1^ cm^−1^. For TAMRA-labeled peptides, absorbance at 552 nm and an extinction coefficient of 90,000 M^−1^ cm^−1^ were used. For AF488-labeled peptides, absorbance at 498 nm and an extinction coefficient of 71,000 M^−1^ cm^−1^ were used.

### Protein expression and purification

The pGBHPS-Nur^S^-YC plasmid was transformed into *E. coli* BL21(DE3) (New England Biolabs, C2527I) to express Nur lacking its signal peptide (Nur^S^). A single tyrosine and cysteine residue were inserted at the C-terminus for quantification and modification, respectively. A single colony was inoculated into LB medium supplemented with 50 µg/mL ampicillin and cultured overnight at 37 °C. The overnight culture was diluted 1:100 in fresh 2 × YT medium with 50 µg/mL ampicillin and grown at 37 °C for 3 h until the optical density at 600 nm (OD_600_) reached 0.5–1.0. Protein expression was induced by adding 0.1 mM isopropyl β-D-1-thiogalactopyranoside (IPTG), followed by incubation at 16 °C overnight. Cells were harvested by centrifugation at 5000 × g for 10 min at 4 °C, and then stored at −80 °C until further use.

For purification, cell pellets from 1 L of culture were resuspended in 30 mL wash buffer (50 mM Tris-HCl (pH 8.0), 500 mM NaCl, 10 mM imidazole) and boiled for 25 min. After centrifugation at 10,000 × g for 20 min at 4 °C, the supernatant was mixed with 5 mL Ni-NTA agarose resin (QIAGEN, 30210) and applied to a gravity-flow column. The column was washed with 50 mL of wash buffer, and bound proteins were eluted with 10 mL of elution buffer (50 mM Tris-HCl (pH 8.0), 500 mM NaCl, 200 mM imidazole). The eluates were concentrated to approximately 1.5–2.0 mL using Amicon Ultra-4 centrifugal filter units with a 10 kDa cutoff (Merck, UFC801024). The His-tag was removed by overnight incubation with 10–20 µL of PreScission Protease (Cytiva, 27084301) at 4 °C. Further purification was performed using a Mono S 5/50 GL cation exchange column (GE Healthcare, 17-5168-01) on an AKTA system at 4 °C. The column was equilibrated with buffer A (20 mM Tris-HCl (pH 8.0), 100 mM NaCl) and proteins were eluted using a linear gradient of buffer B (20 mM Tris-HCl (pH 8.0), 2 M NaCl) over 20 column volumes. The eluted fractions were collected in 1 mL aliquots. Protein purity was checked by SDS-PAGE with CBB staining (EzStain AQua, ATTO, AE-1340). Protein concentrations were determined using the Micro BCA Protein Assay Kit (Thermo Fisher Scientific, 23235).

### MIC assays

All bacterial and fungal strains used in the minimum inhibitory concentration (MIC) assays are listed in Suppl. Table 1. The tested organisms included *Escherichia coli*, *Salmonella enteritidis*, *Pseudomonas aeruginosa*, *Klebsiella pneumoniae*, *Acinetobacter baumannii*, *Serratia marcescens*, *Staphylococcus aureus*, *Streptococcus pyogenes*, *Enterococcus faecalis*, *Mycobacterium smegmatis*, *M. abscessus*, *M. avium*, *M. intracellulare*, and *Candida albicans*. *E. coli* strains ATCC25922, BW25113, JW0368 (*sbmA* transporter mutant), and *E. coli* BL21(DE3) Star were used.

MICs were determined using the broth micro-dilution method according to the Japanese Society of Chemotherapy. The MIC measurement conditions for each microorganism were as follows. *E. coli*, *S. enteritidis*, *S. marcescens*, *K. pneumoniae*, *S. aureus*, *A. baumannii*, and *E. faecalis* were cultured in Mueller–Hinton Broth (MHB; Becton Dickinson) at 37 °C for 18 h. *P. aeruginosa* was cultured in Mueller–Hinton II Broth (MHB II; Becton Dickinson) at 37 °C for 18 h. *M. smegmatis* was cultured in MHB at 37 °C for 42 h. *C. albicans* was cultured in MHB at 27 °C for 42 h. *S. pyogenes* was cultured in MHB for 18 h at 37 °C under aerobic conditions containing 5% CO_2_. *M. abscessus*, *M. avium*, and *M. intracellulare* were cultured in Middlebrook 7H9 medium (Becton Dickinson) supplemented with 10% ADC Enrichment, and the MIC was determined after incubation for 4, 14, and 14 d, respectively.

### MBC assays

To determine whether Nur peptides are bactericidal or bacteriostatic, the minimum bactericidal concentration (MBC) was determined following the MIC assay. For this purpose, the Gram-negative bacterium *E. coli* (K-12) and the Gram-positive bacterium *S. aureus* (FDA209P) were used. Following the 18-h incubation of the MIC assay, 10 µL samples were taken from wells showing no visible bacterial growth (1×MIC to 4×MIC) and spread onto Mueller–Hinton agar plates. The plates were incubated at 37 °C for 18–24 h, after which colony formation was evaluated. The MBC was defined as the lowest concentration yielding no detectable colonies on agar plates, corresponding to ≥ 99.9% killing of the initial inoculum (5 × 10⁵ CFU/mL). Chloramphenicol and amikacin were used as bacteriostatic and bactericidal controls, respectively. Compounds were classified as bactericidal when the MBC/MIC ratio was ≤ 4 and bacteriostatic when the ratio exceeded 4. All experiments were performed in triplicate.

### CD spectroscopy

Circular dichroism (CD) spectroscopy was performed using a spectropolarimeter (JASCO, J-820) equipped with a temperature controller (JASCO, PTC-423L). Measurements were conducted in a 1-mm path length quartz cuvette at 25 °C. Peptide solutions (0.2 mg/mL of C42 or Magainin 2 F12W) were prepared in water, with or without 20 mM SDS (membrane-mimicking conditions). Spectra were recorded in the 180–280 nm range with five accumulations. The baseline spectrum of water was subtracted from each sample to correct for background. Secondary structure contents were estimated using the BeStSel algorithm^88^.

### NMR spectroscopy

The synthesized C42 was dissolved in HEPES buffer or DMSO-d6 to a final concentration of 0.1 mM. All NMR measurements were performed at 25 °C on a Bruker 800 MHz AVANCE NEO equipped with a CPTCI probe. The excitation sculpting method was used for water signal suppression of HEPES buffer samples. The MLEV method was used for mixing in the TOCSY experiment, and the mixing time was 70 ms. TopSpin 4.4.1 (Bruker Co. Ltd.) was used to process the NMR data, and Sparky 3.113 was used to plot the spectra.

### Bacterial liquid growth assay

For liquid growth assays under different culture conditions, *E. coli* CJW7020 expressing L1-msfGFP or *E. coli* carrying the pBAD-mGFP plasmid was grown to log phase, adjusted to an OD_600_ of 0.1 with fresh medium, and mixed with C42 or antibiotics at the indicated concentrations in MHB II, LB, or M9 medium. The mixtures were dispensed into clear-bottom 96-well plates at 200 µL per well and incubated at 37 °C with shaking. OD_600_ was monitored over time using an Infinite 200 PRO plate reader (Tecan).

### Microscopic imaging of bacteria

*E. coli* strains expressing fluorescent reporters for cellular organization, fluorescent marker proteins, or Nur peptide fusions were used. These included ATCC25922, JCM5491, CJW7020 (expressing L1-msfGFP)^35^, an HU-mCherry-expressing strain^89^, an L1-msfGFP/HU-mCherry strain, and BL21(DE3) or CJW7020 derivatives harboring pBAD-based expression constructs. Single colonies grown on LB agar plates were inoculated into LB medium (Nacalai Tesque, 20066-95) for pre-culture and then subcultured into the medium indicated for each experiment, including LB medium (Nacalai Tesque, 20066-95), Mueller–Hinton II Broth (MHB II; Millipore, 90922), or M9 minimal medium (MP Biomedicals, 3037-012) supplemented with 2% glycerol and 2 mg/mL tryptone. Cells were grown to log phase before imaging or were further incubated to later growth phases. Bacterial samples were immobilized on poly-L-lysine (PLL; Sigma-Aldrich, P8920)-coated glass surfaces for microscopy. Depending on the experiment, samples were mounted either between cover glasses with the bottom glass surface pre-coated with PLL, or in custom-made silicone microchambers prepared by punching holes in a 3-mm-thick silicone pad (Sansyo, 94-0745) and sandwiching the pad between cover glasses, with the bottom glass pre-coated with PLL. Fluorescence images were acquired using confocal laser microscopes (Olympus, FV3000; Nikon, A1 with ECLIPSE Ti2; Zeiss, LSM800; or Zeiss, LSM900 with Airyscan 2), as indicated for each experiment.

For analyses of exogenously added Nur peptides and drug-treated cells, cells grown to the indicated growth phase were adjusted to an OD_600_ of 0.1 unless otherwise indicated and incubated with or without Nur peptides or antibiotics at 37 °C. Specifically, cells were treated with 12 µM C42-FITC (Fig. 2a and Suppl. Figs. 2a and 7c), 36 µM C42-AF488 (Suppl. Fig. 2c), 10 µM C42-TAMRA (Fig. 3c), a range of 1.7–13.8 µM C42-TAMRA (Suppl. Fig. 2e), 14 µM C42^16K^-FITC (Suppl. Fig. 3a), 20 µM unlabeled C42 (Figs. 5d), 7.5–20 µM unlabeled C42 (Suppl. Fig. 9d), or 13 µM unlabeled C42 (Suppl. Figs. 9e and 10f). For Nur peptide and antibiotic comparison experiments (Fig. 5e and Suppl. Fig. 10d, e), cells were treated with 13 µM C62, 13 µM C42, 156 µM rifampicin, 4,721 µM kasugamycin (MedChemExpress, HY-B1864B), or 50 µM chloramphenicol (Nacalai Tesque, 08027-72). Incubation times were 30 min for Fig. 3c and Suppl. Fig. 7c, 30–60 min for Suppl. Figs. 2c, 2e, 9d, 9e, and 10f, and 1 h for Fig. 5e and Suppl. Fig. 10d, e. Other time points are indicated in the corresponding figure panels. After mounting, observations were performed using the confocal laser microscopes described above, with or without nucleic acid staining.

For membrane staining and high-resolution imaging of peptide internalization, *E. coli* strain JCM5491 was pre-cultured in 5 mL of LB medium (Nacalai Tesque, 20066-95) at 37 °C for 2 h. Then, 300 µL of the culture was mixed with 700 µL of PBS containing 1 µL of SynaptoRed C2 (Biotium, 70021) and incubated in the dark for 30 min to label membranes. A 49 µL aliquot of the stained cells was then mixed with 1 µL of 1 mM C42-FITC (final: 20 µM). Water was used as a negative control. Images were captured immediately using a confocal laser microscope (Zeiss, LSM900 with Airyscan 2) with a ×63 objective lens (NA 1.4). For quantification, regions of interest (ROIs) containing up to 15 cells per field were selected from images obtained using a confocal laser microscope (Zeiss, LSM800) with a ×63 objective lens (NA 1.4), and intracellular FITC intensity was measured using Fiji/ImageJ software. Data from two independent trials were pooled for analysis.

To observe the localization or dynamics of pBAD-expressed Nur peptide fusions or other proteins, *E. coli* BL21(DE3) or CJW7020 derivatives harboring pBAD-based expression constructs were inoculated into LB medium (Nacalai Tesque, 20066-95) for pre-culture and then diluted 1:100 into the indicated medium, including LB medium or M9 minimal medium (MP Biomedicals, 3037-012) supplemented with 2% glycerol and 2 mg/mL tryptone. For most imaging experiments, cells were grown at 37 °C to an OD_600_ of 0.1, and expression from pBAD-based constructs was induced by adding L-arabinose (final: 0.2%). For pBAD experiments with Nur peptide treatment, L-arabinose was added at the time of 1:100 subculture to induce expression before peptide treatment and log-phase imaging. Cells were observed using confocal laser microscopes (Zeiss, LSM800 or Olympus, FV3000), with or without nucleic acid staining.

For nucleic acid staining, Hoechst 33342 (Thermo Fisher Scientific, 62249, 1:2000) or DAPI (Dojindo, AF172, 1:200) was used for DNA, and Nucleolus Bright Red (Dojindo, N512, 1:2000) was used for RNA. Cells, with or without 4% paraformaldehyde (PFA) pre-fixation, were incubated with each nucleic acid stain, and fluorescence signals were visualized using confocal laser microscopes (Zeiss, LSM800 or Olympus, FV3000).

### Microscopic imaging of eukaryotic cells

*Drosophila* S2R+ cells were cultured at 25 °C in glass-bottom dishes (MatTek, P35G-1.5-14-C), while human U2OS cells were cultured at 37 °C in 5% CO_2_. Cells were incubated with or without C42 peptides for the indicated times under the following conditions. For Suppl. Fig. 3d, cells were incubated with C42-TAMRA (final: 10 or 50 µM) for 1 h in PBS, serum-free Schneider’s *Drosophila* medium (Gibco, 21720001), or Schneider’s *Drosophila* medium (Gibco, 21720001) containing 10% heat-inactivated FBS. For Suppl. Fig. 5d–g, cells were incubated with C42-FITC (final: 25 µM) for 30 min in serum-free Schneider’s *Drosophila* medium (Gibco, 21720001) for S2R+ cells or in PBS for U2OS cells. For nucleic acid staining, cells were incubated with peptides for 30 min and then fixed with 4% PFA in PBS for 5 min at room temperature. After fixation, cells were permeabilized with 1% Triton X-100 in PBS for 20 min and washed three times with PBS. DNA and RNA were stained with Hoechst 33342 (Thermo Fisher Scientific, 62249, 1:2000) and Nucleolus Bright Red (Dojindo, N512, 1:1000), respectively. Fluorescence images were acquired using a confocal laser microscope (Olympus, FV3000) with a ×100 objective lens.

### Co-culture assay of eukaryotic and bacterial cells

*Drosophila* S2R+ cells were cultured at 25 °C in Schneider’s *Drosophila* Medium (Gibco, 21720001) supplemented with 10% heat-inactivated FBS (Life Science Production, S-001A-BR) without antibiotics in glass-bottom dishes (MatTek, P35G-1.5-14-C). *E. coli* strain CJW7020 expressing L1-msfGFP was cultured in Mueller–Hinton II Broth (MHB II; Millipore, 90922) at 37 °C to the log growth phase (OD_600_: 0.1–0.3). The *E. coli* culture was added to the S2R+ cell suspension at a 1:1 volume ratio, resulting in a final FBS concentration of 5%. C42-TAMRA was immediately added to a final concentration of 50 µM. The co-cultures were incubated for 1 h at 25 °C and imaged using a confocal laser microscope (Olympus, FV3000).

### Quantification of peptide concentration using confocal microscopy

Fluorescence images of the AF488-labeled C42 peptide (C42-AF488) were acquired using a confocal laser scanning microscope (Olympus, FV3000). To generate a calibration curve, C42-AF488 was dissolved in Mueller–Hinton II Broth (MHB II; Millipore, 90922) at concentrations ranging from 3.6 to 1440 µM and mounted between glass slides. Saturated values at the highest concentrations were excluded from calibration fitting, and the calibration curve was fitted using the unsaturated linear range. Laser power, detector high voltage, pinhole size, and other acquisition settings were kept constant for all measurements to ensure quantitative comparison. Quantitative image analysis was performed using ImageJ software. The mean fluorescence intensity of each image was measured. To correct for peripheral shading observed in low-magnification images, the central regions of images were digitally cropped to match the field of view of higher-magnification images. Background fluorescence intensity, measured from a medium-only control, was subtracted from all intensity values. The resulting calibration curve was plotted as mean fluorescence intensity versus peptide concentration. For cellular measurements, *E. coli* cells were treated with 36 µM C42-AF488 for 30–60 min and imaged using the same acquisition settings. Intracellular fluorescence intensity was measured from cell regions, background-subtracted, and converted to an estimated peptide concentration using the calibration curve.

### FRAP measurement

Fluorescence recovery after photobleaching (FRAP) was measured using confocal laser microscopes (Zeiss, LSM800 or Olympus, FV3000). Images were acquired under low-bleaching conditions by minimizing the excitation laser power during time-lapse imaging. Regions of interest (ROIs) were photobleached using 100% laser power with the minimum irradiation time required to achieve sufficient bleaching, and fluorescence recovery was recorded over time. For each experiment, samples to be compared were imaged and photobleached under the same acquisition settings, including objective lens, zoom, pinhole, excitation laser power, detector gain, scan speed, and frame interval. Fluorescence intensities were normalized to the pre-bleach values, and recovery curves were fitted to a single exponential function.

### Membrane permeability assay using SYTOX Green

Inner membrane permeability was evaluated using a SYTOX Green uptake assay. The dye exhibits minimal fluorescence outside *E. coli* cells; however, upon membrane permeabilization, it penetrates the cells, binds to DNA, and results in a marked increase in fluorescence intensity. *E. coli* ATCC25922 was cultured to mid-log phase (OD_600_ = 0.5–1.0), harvested by centrifugation at 2300 ×g for 10 min, and washed three times with PBS buffer. The bacterial suspension was then adjusted to OD_600_ = 0.1 and incubated with 150 nM SYTOX Green (Thermo Fisher Scientific, S7020) for 15 min at 37 °C in the dark. Subsequently, 50 µL of this stained bacterial suspension was mixed with 50 µL of two-fold serially diluted peptide solutions in a 96-well black plate. Fluorescence was monitored for 60 min using a multimode microplate reader (Varioskan LUX, Thermo Fisher Scientific) with excitation at 507 nm and emission at 525 nm.

### GUV preparation and experiment

Giant unilamellar vesicles (GUVs) composed of DOPG and DOPC (40:60 molar ratio) were prepared by the natural swelling method, as previously described in ref. ^90^. A total of 2.0 µmol of dry lipid film was hydrated with 20 µL of water in a glass vial and incubated at 45 °C for 7 min. The pre-hydrated lipid was then added to 1 mL of buffer containing 20 mM HEPES (pH 7.4), 150 mM NaCl, 1 mM egtazic acid, and 0.10 M sucrose, followed by incubation for 2–3 h at 37 °C. The supernatants were collected by centrifugation at 13,000 ×g for 20 min at 20 °C and passed through a Nucleopore membrane with 10 µm diameter pores (GE Healthcare, Whatman) using buffer containing 0.10 M glucose at a flow rate of 1 mL/min for 1 h at room temperature. The GUVs retained on the filter were collected as purified vesicles. Purified GUVs were transferred to microchambers pre-coated with 0.10% (w/v) BSA and observed under a confocal laser microscope (Olympus, FV3000) equipped with a ×60 objective lens. For single-GUV experiments, fluorescently labeled Nur peptides were applied using a glass micropipette with a 20 µm diameter tip, positioned approximately 60 µm from the target GUV. The pressure difference was adjusted to −30 Pa and monitored using a differential pressure transducer (Validyne, DP15), a pressure amplifier (Validyne, PA501), and a digital multimeter. For the GUV suspension method, purified GUVs were mixed with labeled peptide (final: 16 µM) and observed under a confocal laser microscope (Olympus, FV3000).

### Spot assay

*E. coli* strain BL21 (DE3; New England Biolabs, C2527I) transformed with Nur peptides in pBAD vector was cultured in M9 minimal medium (MP Biomedicals, 3037-012) supplemented with 2% glycerol, 2 mg/mL tryptone, and 50 µg/mL ampicillin at 37 °C until the OD_600_ reached 0.3–0.4. Then, 3 µL aliquots of 10-, 30-, 100-, and 1,000-fold diluted cultures were spotted onto M9 agar plates containing the same supplements and either 0.4% glucose (repression condition) or 0.2% L-arabinose (induction condition). The plates were incubated at 37 °C for 12–16 h and then photographed.

### Protein proximity labeling by TurboID

*E. coli* strain BL21 (DE3; New England Biolabs, C2527I) transformed with pBAD constructs encoding TurboID-C42 variants (WT or 16 A) or TurboID alone was cultured overnight at 37 °C in LB medium supplemented with 0.4% glucose and 50 µg/mL ampicillin. A 50 µL aliquot of the overnight culture was inoculated into 5 mL of M9 minimal medium containing 2% glycerol, 2 mg/mL tryptone, and 50 µg/mL ampicillin, and grown at 37 °C. When the OD_600_ reached approximately 0.3, expression was induced by adding L-arabinose to a final concentration of 0.2%, and biotinylation was initiated by adding biotin to a final concentration of 100 µM. After incubation for 0, 5, or 24 h at 37 °C, cells were harvested by centrifugation at 5,000 ×g for 3 min at 4 °C. The cell pellets were washed twice with PBS to remove excess biotin and residual culture medium, and then used for subsequent enrichment of biotinylated proteins.

*E. coli* cell pellets were dissolved in 500 µL of PTS buffer (100 mM NH_4_HCO_3_, 12 mM sodium deoxycholate, 12 mM sodium N-lauroylsarcosinate) and centrifuged at 21,600 ×g for 10 min at 4 °C. The resulting supernatant was incubated with Pierce High Capacity Streptavidin Agarose (Thermo Fisher Scientific) at 4 °C for 2 h with gentle rotation. After incubation, the beads were washed once with urea wash buffer (100 mM HEPES-NaOH (pH 7.8), 200 mM NaCl, 1% Triton X-100, 2 M urea), once with ConA wash buffer (100 mM HEPES-NaOH (pH 7.8), 500 mM NaCl, 1% Triton X-100), and twice with PTS buffer. The washed beads were resuspended in 100 µL of PTS buffer and used for downstream applications.

Enzymatic digestion of biotinylated proteins was performed on-bead according to the phase transfer surfactant (PTS) protocol as previously described in refs. ^91,92^ with some modifications. The beads were first incubated with 10 mM dithiothreitol (DTT) at 60 °C for 30 min to reduce disulfide bonds, followed by alkylation with 22 mM iodoacetamide at 37 °C for 30 min in the dark. The samples were then diluted with 100 mM NH_4_HCO_3_ solution to a final volume of 500 µL and digested for 18 h at 37 °C with 0.4 µg of trypsin (Sigma-Aldrich, 3708969001) in the dark. After digestion, an equal volume of ethyl acetate was added, and the mixture was acidified with 0.5% trifluoroacetic acid (TFA) and mixed well to transfer the detergents into the organic phase. After the samples were centrifuged at 15,700 × g for 4 min at room temperature, the aqueous phase containing peptides was collected. The peptides were concentrated using a centrifugal evaporator (EYELA) and desalted using a Mono-Spin C18 column (GL Sciences, 5010-21701). The eluate was dried and stored until LC-MS/MS analysis.

LC-MS/MS-based proteome analysis was performed as previously described in refs. ^91,92^ with minor modifications. The dried and desalted peptides were dissolved in distilled water containing 2% acetonitrile and 0.1% TFA. The samples were analyzed on a Q Exactive Plus MS spectrometer (Thermo Fisher Scientific) equipped with a Dionex Ultimate 3000 nano ultra-HPLC system (Thermo Fisher Scientific). The peptides were first loaded onto a trap column (0.3 × 5 mm L-column ODS, Chemicals Evaluation and Research Institute) and a capillary column (0.1 × 150 mm L-column ODS, Chemicals Evaluation and Research Institute) at a flow rate of 20 µL/min. The loaded peptides were separated by a gradient using mobile phases A (1% formic acid in distilled water) and B (1% formic acid in acetonitrile) at a flow rate of 300 nL/min (0% to 30% B in 35 min, 30% to 50% B in 5 min, 50% to 95% B in 0.1 min, 95% B for 9.8 min, 95% to 0% B in 0.1 min, and 0% B for 5 min). The eluted peptides were ionized by electrospray (2.0 kV) and introduced into the MS equipment (positive ion mode, data-dependent MS/MS). Each of the most intense precursor ions (up to the top 10) was isolated and fragmented by higher collision energy dissociation with the normalized collision energy (27%). For full MS scans, the scan range was set to 350–1500 m/z at a resolution of 70,000, and the automatic gain control (AGC) target was set to 3 × 10^6^ with a maximum injection time of 60 ms. For MS/MS scans, the precursor isolation window was set to 1.6 m/z at a resolution of 17,500, and the AGC target was set to 5 × 10^5^ with a maximum injection time of 100 ms. The Orbitrap mass analyzer was operated with the “lock mass” option to perform shotgun detection with high accuracy. The raw spectra were extracted using Proteome Discoverer 3.1 (Thermo Fisher Scientific) and searched against the *E. coli* database (Taxon ID: 469008). The parameter of the cleavage was set to trypsin, and the missed cleavages were allowed up to 2. The mass tolerances were set to 10 ppm for the precursor ion and 0.02 Da for the fragment ion. As for protein modifications, carbamidomethylation ( + 57.021 Da) at cysteine was set as a static (fixed) modification, oxidation ( + 15.995 Da) at methionine as a dynamic (non-fixed) modification, and acetylation ( + 42.011 Da) at amino terminus as a dynamic modification. The amount of each peptide was semi-quantified using the peak area with Precursor Ions Quantifier in Proteome Discoverer 3.1. According to the manufacturer’s procedure, relative levels of proteins were quantified based on unique peptides without shared peptides.

Proteins quantified in three independent experiments per condition were screened using the 5-h data. TurboID-C42^WT^ was compared with TurboID alone and TurboID-C42^16A^. Nominal *P* values were calculated using two-sided unpaired Student’s *t*-tests assuming equal variances. Proteins were retained as C42-enriched candidates when both comparisons showed *P* < 0.5 and > 1.5-fold enrichment and the mean scaled abundance of TurboID-C42^WT^ exceeded 150 at both 5 and 24 h, yielding 58 proteins. Gene Ontology enrichment analysis of these proteins was performed using the Database for Annotation, Visualization and Integrated Discovery (DAVID), with the *E. coli* BL21(DE3) background and all Biological Process and Molecular Function terms. *P*-values were calculated using DAVID’s modified one-tailed Fisher exact test (EASE score). Representative terms with *P* < 0.05 were selected for visualization to reduce redundancy.

### Raman imaging

A 100 µL suspension of *E. coli* expressing iRFP or iRFP-C42 was seeded onto a glass-bottom dish coated with PLL. The dish was placed at room temperature for 10 min and then washed with PBS. A coverslip was put on the glass substrate of the dish to suppress cell motion. Raman images of *E. coli* were acquired using a confocal Raman system (Tokyo Instruments Inc., Nanofinder flex2) integrated with an inverted microscope (Nikon, ECLIPSE Ti2), as previously described in refs. ^93,94^. 532-nm cw laser beam from a DPSS laser system (Lighthouse Photonics, Sprout Solo) was focused on the sample using a water-objective lens (Plan Apo IR 60x/NA = 1.27, Nikon) and the Raman signals were collected with the same objective. Before and after each Raman measurement, fluorescence images were obtained using a spinning disk confocal unit (Crest Optics, X-light V2) combined with the microscope. The excitation and observation wavelengths for the fluorescence measurements were 640 and 700 nm, respectively. The IGOR Pro program package (WaveMetrics) was used to analyze Raman and fluorescence images. The concentration ratio between DNA and RNA was estimated using the band ratio of the pyrimidine band at 787 cm^−1^ and the symmetric phosphodiester band at 814 cm^−1^ in the average Raman spectra of the regions of interest. The concentration ratio of proteins and nucleic acids (DNA/RNA) was estimated based on the intensity ratio between the amide I band at 1650 cm^−1^ and the pyrimidine band.

Three-dimensional refractive index imaging of *E. coli* seeded onto a glass-bottom dish was performed using a holographic microscope (Tomocube, HT-2H). Prior to the measurements, the refractive index values were calibrated using polystyrene beads with a diameter of 2.5 µm (micromod Partikeltechnologie GmbH, 01-00-253). Based on the refractive index values obtained from the regions of interest, the total concentration of biomolecules was calculated by assuming a linear relationship between the refractive index and biomolecular concentration, as previously described^95^. Furthermore, the individual concentrations of proteins, DNA, and RNA were estimated using their relative ratios derived from the Raman spectral data.

### Sucrose density gradient centrifugation

*E. coli* strain MRE600 or JCM5491 was grown in 1 L of LB medium at 37 °C until the OD_600_ reached 0.5–0.8. Cells were harvested by centrifugation at 4000 × g for 10–20 min at 4 °C and resuspended in 10 mL of RBS buffer (20 mM HEPES (pH 7.6), 30 mM NH_4_Cl, 10 mM Mg(OAc)_2_) to maintain the stability of the 70S ribosome complex. To lyse the cell wall, 1 mL of the cell suspension was mixed with 10 µL of 50 mg/mL lysozyme solution (FUJIFILM Wako, 123-06721) and incubated overnight at −80 °C. Following lysis, samples were centrifuged at 15,000 ×g for 15 min at 4 °C, and the supernatants were collected. For sucrose density gradient analysis, lysates were mixed with 5 µL of FITC-labeled C42 peptide variant solution to a final peptide concentration of 0.164 µM and carefully layered onto a 10–40% (w/v) sucrose gradient prepared using a Gradient Station Model 153 (BioComp Instruments) in an open-top, thin-wall, ultra-clear tube (Beckman Coulter, 344058). Samples were ultracentrifuged at 141,000 × g for 5 h at 4 °C in an ultracentrifuge (Beckman Coulter, L-100K). Gradients were fractionated, and each fraction was analyzed by measuring absorbance at 260 nm and FITC fluorescence (excitation: 495 nm; emission: 520 nm) using a microplate reader (Tecan, Infinite 200 Pro).

### In vitro mRNA synthesis

mRNA was synthesized using the Thermo T7 Transcription Kit (Toyobo, TSK-101) following the manufacturer’s protocol. Briefly, 1 µg of pET22b-sfGFP plasmid was mixed with 19 µL of the transcription premix containing rNTPs, T7 RNA polymerase, and RNase inhibitor, and incubated at 40 °C for 2 h. After transcription, template DNA was removed by DNase I treatment. The resulting RNA was purified using the Zymo-Spin IC Column Kit (Funakoshi, C1004-50). RNA purity was verified by agarose gel electrophoresis stained with GreenView nucleic acid stain (Relyon, N100), and RNA concentration was determined by absorbance at 260 nm.

### gDNA extraction from bacteria

Genomic DNA was extracted from *E. coli* strain JCM5491 using TRIzol reagent (Ambion, 15596026) and purified with the DNeasy Blood & Tissue Kit (QIAGEN, 69504). Bacterial cultures were grown overnight at 37 °C and harvested by centrifugation at 6,000 ×g for 10 min at 4 °C. Cell pellets were lysed in 1 mL of TRIzol at room temperature and mixed with 200 µL of chloroform. After centrifugation at 12,000 × g for 15 min at 4 °C, the interphase and lower organic phase were collected and mixed with 300 µL ethanol. The DNA-containing pellet was washed with buffer (0.1 M sodium citrate (pH 8.5), 10% ethanol) and 75% ethanol. Following centrifugation at 2000 × g for 5 min at 4 °C, the pellet was air-dried and dissolved in 8 mM NaOH. After centrifugation at 12,000 × g for 10 min at 4 °C, the supernatant was further purified using the DNeasy Kit and concentrated by ethanol/acetic acid precipitation. DNA concentration was measured by absorbance at 260 nm, and purity was confirmed by agarose gel electrophoresis.

### Electrophoretic mobility shift assay

70S ribosomes (New England Biolabs, P0763S) and transfer RNA (tRNA; Sigma-Aldrich, R1753) from *E. coli* were used as supplied. Plasmid DNA, genomic DNA, and mRNA were prepared as described above. The 70S ribosomes and nucleic acids were adjusted to a final concentration of 50 ng/µL and incubated separately with C42 variants (WT, 4 A, or 16 A) at final concentrations of 0, 5, 10, 20, or 50 µM in 20 mM Tris-HCl (pH 8.0) for 30 min at room temperature. After adding 6× loading buffer (20 mM Tris-HCl (pH 8.0), 15% (w/v) Ficoll 400), samples were resolved by electrophoresis on 1% agarose gels prepared in 1× TAE buffer at 100 V for 30–35 min. A 1 kb DNA Ladder PLUS (FastGene, NE-MWD1P) and a 0.125–6.0 kb RNA Ladder (Nippon Gene, 311-06261) were used as size markers for DNA and RNA samples, respectively. Gels were stained with GreenView nucleic acid stain (Relyon, N100) for 30 min and imaged using a FAS-DiGiPRO system (Nippon Genetics, GP-07LED).

### In vivo transcription and translation assay

*E. coli* strain BL21 (DE3; New England Biolabs, C2527I) transformed with mGFP in a pBAD vector was cultured overnight in LB medium supplemented with 0.4% glucose and 50 µg/mL ampicillin at 37 °C. A 50 µL aliquot of the overnight culture was transferred into 5 mL of M9 minimal medium containing 2% glycerol, 2 mg/mL tryptone, and 50 µg/mL ampicillin, and incubated at 37 °C until reaching the log growth phase. The OD_600_ was adjusted to 0.1 with fresh medium. 940 µL of the bacterial suspension was then mixed with 20 µL of antimicrobial agents and incubated at 37 °C with shaking. Stock solutions of rifampicin and chloramphenicol were prepared in DMSO, and kasugamycin and C42 were dissolved in water. After 60 min of incubation, 40 µL of 5% arabinose solution was added to induce mGFP expression, resulting in a final concentration of 0.2%. The mixtures were incubated for an additional 60 min and harvested by centrifugation at 5000 × g for 3 min at 4 °C. The cell pellets were used for downstream RNA extraction and quantitative PCR (qPCR).

For RNA extraction, cell pellets were lysed using 1 mL of TRIzol reagent (Ambion, 15596026) and processed by chloroform and isopropanol precipitation. The RNA pellet was resuspended in RNase-free water. Genomic DNA was removed using the PrimeScript RT reagent Kit with gDNA Eraser (TaKaRa Bio, RR047A), and complementary DNA (cDNA) synthesis was performed using random primers. qPCR was carried out using the SsoAdvanced Universal SYBR Green Supermix (Bio-Rad, 1725270) on a CFX96 Real-Time PCR system (Bio-Rad). Reactions were set up in a Hard-Shell 96-Well PCR Plate (Bio-Rad, HSP9655) sealed with optical adhesive film (Bio-Rad, 1814030). The primer sets used for qPCR were: mGFP (Forward: 5’-CGGTGAAGGTGAAGGTGATGCTAC-3’, Reverse: 5’-GTGACTAAGGTTGGCCATGGAACTG-3’) and 16S rRNA (Forward: 5’-CAACCTGGGAACTGCATCTGATACTG-3’, Reverse: 5’-CCTCCAGATCTCTACGCATTTCACC-3’).

Thermal cycling was performed under the following conditions: 95 °C for 30 s, followed by 40 cycles of 95 °C for 10 s and 60 °C for 20 s. Melt curve analysis was performed after amplification. A standard curve was generated using serial dilutions of cDNA over a 10^4^-fold range.

For western blot analysis, parallel 1-mL cultures were prepared and treated as described above. After centrifugation at 5,000 ×g for 3 min at 4 °C, the cell pellets were resuspended in 50 µL of 1× SDS sample buffer, prepared by diluting 4× Laemmli sample buffer (Bio-Rad, 1610747) supplemented with 2-mercaptoethanol, and heated at 95 °C for 5 min. Aliquots of the resulting lysates were separated by 10% SDS-PAGE and transferred to polyvinylidene difluoride (PVDF) membranes. Membranes were blocked with 1% skim milk (Morinaga) in TBS-T (1× TBS with 0.1% Tween 20) and incubated with an anti-GFP primary antibody (Roche, 11814460001, 1:1000), followed by an HRP-conjugated anti-mouse IgG secondary antibody (Jackson ImmunoResearch, 715-035-151, 1:5000). Signals were detected using an enhanced chemiluminescence (ECL) reagent (PerkinElmer, NEL120001EA) and imaged using a Fusion Solo S imaging system (Vilber Bio Imaging).

To assess bacterial growth, 188 µL of log-phase culture (adjusted to the OD_600_ of 0.1) was mixed with 4 µL of antimicrobial agents and 8 µL of 5% arabinose in a 96-well plate. The cultures were incubated at 37 °C with shaking, and OD_600_ was monitored.

### Global nascent protein synthesis assay using AHA and puromycin labeling

To assess global nascent protein synthesis in vivo, *E. coli* MG1655 cells were metabolically labeled with L-azidohomoalanine (AHA; Tokyo Chemical Industry, A3550). Overnight cultures were diluted into M9 minimal medium (3.0 g/L KH_2_PO_4_, 6.8 g/L Na_2_HPO_4_, 0.50 g/L NaCl, 1.0 g/L NH_4_Cl) supplemented with 2% glycerol and 2 mg/mL tryptone to an initial OD_660_ of 0.01. Cells were grown at 37 °C with shaking at 180 rpm. When cultures reached early log phase (OD_660_ approximately 0.1), they were divided into separate tubes and treated with C42 at the indicated concentrations for 15 min at 37 °C. AHA was then added to a final concentration of 2.0 mM, and cultures were incubated for an additional 15 min at 37 °C. Cells were collected by centrifugation and stored at −30 °C.

Cell pellets were resuspended in 100 µL lysis buffer (50 mM Tris-HCl (pH 8.0), 1% SDS) and boiled at 95 °C for 5 min. To disrupt genomic DNA, Benzonase nuclease (0.2 µL per sample; Merck Millipore, 70664) and MgCl_2_ (final: 1 mM) were added, and samples were incubated at 37 °C for 15 min. Lysates were clarified by centrifugation at 20,000 × g for 5 min at room temperature. Protein concentrations in the lysates were determined by BCA assay (Pierce, 23225). Equal amounts of total protein (75 µg in 50 µL) were subjected to click labeling.

AHA-containing proteins were fluorescently labeled using the Click-iT Protein Reaction Buffer Kit (Invitrogen, C10276) with TAMRA alkyne (Invitrogen, T10183) according to the manufacturer’s instructions, except that TAMRA alkyne was used at one-tenth of the recommended concentration (4.0 µM). Following the click reaction and washing steps, protein pellets were resuspended in lysis buffer, quantified again by BCA assay, adjusted to 1 mg/mL, and aliquots were separated by SDS-PAGE using 12% polyacrylamide gels. TAMRA fluorescence was detected using the Amersham Typhoon scanner (Cytiva). Total protein loading was subsequently visualized using the Quick-CBB staining kit (FUJIFILM Wako, 299-50101) and imaged using the LAS4000 image analysis (FUJIFILM). TAMRA and CBB signal intensities in each lane were quantified using the “Gels” function in Fiji/ImageJ software after background subtraction using the “Subtract Background” function. TAMRA signals were normalized to the corresponding total-protein CBB signals, and the CBB-normalized no-AHA control value was subtracted from each sample value as background. Values were expressed relative to the no-C42 control. The experiment was performed in two independent biological replicates.

For the evaluation using puromycin, we used a method adapted from SUnSET^96^. The culture was performed under the same conditions as for AHA labeling, except that 0.2 mM puromycin dihydrochloride (FUJIFILM Wako, 166-23153) was added instead of AHA. After disrupting the cells under the same conditions described above, puromycin-labeled proteins were separated by SDS-PAGE on 12.5% polyacrylamide gels and transferred onto PVDF membrane using the Trans-Blot Turbo transfer system (Bio-Rad). Membranes were blocked with 2.5% non-fat dry milk (FUJIFILM Wako, 190-12865) in TBS-T (1× TBS with 0.2% Tween 20) for 1 h at room temperature. Membranes were then incubated for 1 h at room temperature with an anti-puromycin primary antibody (Sigma-Aldrich, MABE343, 1:20,000). After three washes with TBS-T, membranes were incubated for 45 min at room temperature with an HRP-conjugated anti-mouse IgG secondary antibody (Sigma-Aldrich, A4416, 1:10,000). After three additional washes with TBS-T, signals were detected using Immobilon Chemiluminescent substrate kit (Merck Millipore, WBKLS050) and imaged using the LAS4000 image analysis (FUJIFILM). For normalization, the same samples were run on a separate gel, stained with the Quick-CBB staining kit (FUJIFILM Wako, 299-50101), and visualized with the LAS4000 image analyzer in DIA mode (FUJIFILM). Signal intensities in each lane were quantified using Multi Gauge version 3.1 (FUJIFILM). Puromycin signals were normalized to the corresponding total-protein CBB signals, and the CBB-normalized no-puromycin control value was subtracted from each sample value as background. Values were expressed relative to the no-C42 control. The experiment was performed in two independent biological replicates.

### In vitro translation assay

In vitro translation inhibition assays were performed using the PUREfrex 1.0 system (GeneFrontier Corporation) to evaluate the inhibitory effects of C42 and its mutants (4 A and 16 A). The mRNA encoding superfolder green fluorescent protein (sfGFP) was synthesized using the in vitro Transcription T7 Kit (TaKaRa Bio). For each reaction, 60 ng of mRNA was added to a 20 µL PUREfrex reaction mixture. sfGFP production was monitored by measuring fluorescence intensity using the Varioskan LUX instrument (Thermo Fisher Scientific), as previously described in ref. ^97^. Experiments were performed independently five times for the time-course analysis and four times for the endpoint dose–response/IC_50_ analysis. Endpoint fluorescence values at 180 min were normalized to the mean of the no-peptide controls for each peptide series, and IC_50_ values were calculated by nonlinear regression analysis using the built-in dose–response inhibition model “[Inhibitor] vs. response — Variable slope (four parameters)” in GraphPad Prism version 10.2.2 (GraphPad Software, Boston, MA, USA).

### In vitro transcription assay

An in vitro transcription inhibition assay was conducted using the Thermo T7 Transcription Kit (Toyobo, TSK-101). For each reaction, 1 µg of pET22b-sfGFP plasmid was mixed with 7 µL of C42 variants (WT, 4 A, or 16 A) or actinomycin D (as a positive control) and 12 µL of the transcription premix supplied with the kit. The reaction mixture was incubated at 40 °C for 2 h. Following transcription, 1 mL of TRIzol reagent (Ambion, 15596026) was added, followed by 200 µL of chloroform. After mixing and standing for 2–3 min, the sample was centrifuged at 12,000 × g for 15 min at 4 °C. The upper aqueous phase (approximately 300 µL), containing RNA, was collected and precipitated with 500 µL of isopropanol (Wako, 166-21671). After incubation at 4 °C for 10 min, the sample was centrifuged at 12,000 × g for 10 min at 4 °C. The RNA pellet was washed with 1 mL of 75% ethanol, centrifuged at 7500 × g for 5 min at 4 °C, and air-dried at room temperature for 5–10 min. The dried pellet was resuspended in 40 µL of RNase-free water, and RNA concentration was measured by absorbance at 260 nm using a NanoPhotometer NP80 Touch (IMPLEN, T81327). The amount of synthesized RNA was quantified using an Agilent 2100 Bioanalyzer with RNA 6000 Nano Reagents Part I (Agilent) and RNA Nano Chips (Agilent), based on the area under the target RNA peak.

### Purification of mCherry-tagged ribosomes

mCherry-tagged ribosomes were purified from *E. coli* strain QC101. The bacteria were first inoculated into 20 mL of LB medium and incubated overnight at 37 °C with shaking. The overnight culture was then added to 1 L of 2 × YT medium and grown at 37 °C with shaking until the OD_600_ reached 0.5–0.8. Cells were harvested by centrifugation at 5000 ×g for 15 min at 4 °C and resuspended in 10 mL of RBS buffer (20 mM HEPES (pH 7.6), 30 mM NH_4_Cl, 10 mM Mg(OAc)_2_) supplemented with RNase inhibitor (Promega, N2611) at 1:200 dilution, to maintain ribosome complex stability. For cell lysis, 100 µL of 50 mg/mL lysozyme (FUJIFILM Wako, 123-06721) was added to 10 mL of the suspension, which was then incubated at −80 °C overnight. Following thawing, lysates were cleared by centrifugation at 15,000 ×g for 15 min at 4 °C. Ribosomes were pelleted by ultracentrifugation at 100,000–200,000 ×g for 3 h at 4 °C. The quality and integrity of the purified ribosomes were assessed by measuring absorbance at 260 nm, agarose gel electrophoresis, and negative staining.

### In vitro condensation experiments

For the *E. coli* extract-based condensation assay, NEBExpress cell-free *E. coli* protein synthesis lysate (New England Biolabs, E5360) was incubated with DHFR-His DNA template at a final concentration of 5 ng/µL at 37 °C for 2 h to generate a complex bacterial lysate environment. FITC-labeled C42 peptide variants were then added to the lysate. The final mixtures contained 50% (v/v) lysate and 100 µM peptide, in the absence or presence of 500 mM NaCl. After spinning down by a tabletop centrifuge, the appearance of the samples was photographed.

For purified ribosome condensation assays, 70S ribosomes were mixed with Nur peptides in Polymix buffer (5 mM K_2_HPO_4_/KH_2_PO_4_ (pH 7.5), 95 mM KCl, 5 mM NH_4_Cl, 5 mM Mg(OAc)_2_, 0.5 mM CaCl_2_, 8 mM putrescine, 1 mM spermidine, and 1 mM DTT). The final concentrations were 0–10 µM for 70S ribosomes and 0–100 µM for Nur peptides. Turbidity of the samples was measured as the optical density at 600 nm. For morphological observation of the assembly states, samples were mounted on glass-bottom dishes (MatTek, P35G-1.5-14-C), and assembly formation was observed using a confocal laser microscope (Olympus, FV3000). To visualize C42 and ribosome localization, Alexa Fluor 488-labeled C42 and mCherry-tagged ribosomes purified from *E. coli* strain QC101 (see above) were used, and FRAP analysis was performed as described above.

For nucleic acid condensation assays, oligonucleotides with or without fluorophore labeling were synthesized by Integrated DNA Technologies. The sequence of the ssDNA was 5’-TGAGAATGAATAGAATGAATAA-3’, with a 5’-FAM modification. For dsDNA, the same sequence was used as the sense strand, annealed to its complementary antisense strand, with FAM attached to the 5’ end of the sense strand. The ssRNA sequence was 5’-UGAGAAUGAAUAGAAUGAAUAA-3’, labeled with TYE665 at the 5’ end. Samples were prepared in Polymix buffer, with a final total charge concentration of 5 mM from nucleic acids, including 4 µM each dye-labeled oligonucleotide. Samples were mounted on glass-bottom dishes (MatTek, P35G-1.5-14-C), and assembly formation was observed using a confocal laser microscope (Olympus, FV3000).

For poly-L-lysine-based multiphase droplet assays, stock solutions of oligo DNA and RNA were prepared at 22 mM total charges, and PLL (Sigma-Aldrich, P6516) was prepared at 100 mM total charges. All stock solutions were prepared in water and stored at −20 °C. In the aqueous mixtures, each lysine residue contributes one positive charge, and each DNA or RNA phosphate group contributes one negative charge. Samples were prepared in buffer containing 150 mM NaCl and 20 mM Tris-HCl (pH 8.0), with a final total charge concentration of 5 mM each from oligonucleotides (including 4 µM dye-labeled oligonucleotide) and PLL. For samples containing multiple oligonucleotide species, the combined total charge concentration of oligonucleotides was adjusted to 5 mM. Similarly, the concentration of purified mCherry-tagged ribosomes (see above) was adjusted to a total charge concentration of 5 mM, estimated from their ribosomal RNA content. Fluorescently labeled C42 (C42-TAMRA or C42-FITC) was added to a final concentration of 5 µM. Assembly formation was observed in a glass-bottom dish (MatTek, P35G-1.5-14-C) using a confocal laser microscope (Olympus, FV3000).

### Negative staining and EM observation

One drop of 50 ng/µL mCherry-tagged ribosomes (see above) purified from *E. coli* strain QC101 in RBS buffer (20 mM HEPES (pH 7.6), 30 mM NH_4_Cl, 10 mM Mg(OAc)_2_) was applied to a carbon-coated grid. Following 1 min of incubation, the grid was stained three times with 1% uranyl acetate, blotting with filter paper after each wash^98^. Data collection was performed on a JEM-1400plus electron microscope (JEOL) operated at 80 kV. Micrographs were collected with an EM-Z12162TTKDC CCD camera (JEOL) at a pixel size of 2.94 Å/pixel. Single-particle analysis was performed using RELION-5 software^99^. Ribosome particles were automatically picked from the 27 micrographs via Laplacian-of-Gaussian auto-picking^100^ and extracted with a box size of 80 pixels and 2× binning. After two rounds of 2D classification and selection of good classes, an initial model was created from 2000 randomly selected particles. The final 3D map was reconstructed from 2,468 particles after two rounds of 3D classification. The reconstructed 3D map was aligned with the previously reported reference map (EMD-15793)^101^.

### Cryo-EM observation

Purified 70S ribosomes (final: 65 nM) were mixed with or without 30 µM C42 in 70S buffer (20 mM HEPES-KOH (pH 7.6), 100 mM KOAc, 10 mM Mg(OAc)_2_, 7 mM 2-mercaptoethanol). A 3 µL aliquot of the mixture was applied onto a Quantifoil R1.2/1.3 Cu 200 grid with an additional 2 nm carbon layer (Quantifoil) and vitrified using a Vitrobot Mark IV (Thermo Fisher Scientific). The grid was imaged using a Glacios cryo-TEM equipped with a Falcon4i direct electron detector (Thermo Fisher Scientific).

### Hemolytic assay

Sheep red blood cells (RBCs; Cedarlane Laboratories, CLD68) were centrifuged at 800 ×g for 5 min at 4 °C, washed three times with PBS, and adjusted to 1.2 × 10^7^ cells/mL using a hemocytometer. A volume of 100 µL of RBC suspension was mixed with 100 µL of 1 mg/mL test peptides and incubated at 37 °C for 1 h. PBS or 20% SDS served as negative and positive controls, respectively. Following incubation, samples were centrifuged at 800 × g for 5 min at 25 °C. Hemolysis was assessed by measuring the absorbance of the supernatant at 405 nm using a Synergy LX multi-mode reader (BioTek). Hemolysis percentage was calculated using values obtained from samples with test compounds (*A*_*test*_) relative to positive (100% lysis; *A*_*pos*_) and negative (untreated; *A*_0_) controls using the following equation:

$${{\rm{Hemolysis}}}\,{{\rm{percentage}}} \; (\%)=\frac{({A}_{{test}}-{A}_{0})}{({A}_{{pos}}-{A}_{0})}\times 100$$

### MTT assay

PANC-1 cells were seeded in a flat-bottomed 96-well plate at a density of 1.0 × 10^4^ cells/well in DMEM and incubated overnight. Then, serially diluted peptides were added and the cells were incubated for 72 h. For L-1210 cells, 0.5 × 10^4^ cells/well were seeded in RPMI 1640 medium, treated similarly, and incubated for 48 h. After the incubation period, 0.5 mg/mL MTT solution (Dojindo, M009) was added to each well and incubated for 4 h. The supernatant was then removed, and 100 µL of DMSO was added to dissolve the formazan crystals. Absorbance was measured at 570 nm using a microplate reader. DMEM or RPMI 1640 without peptide served as the negative control. All measurements were performed using technical triplicates in two independent experiments.

### MTS assay

S2R+ cells (8000 per well) were plated in 96-well plates with 100 µL of medium containing Nur peptides at the indicated concentrations. After 36 h of incubation, 20 µL of MTS reagent (CellTiter 96® AQueous One Solution; Promega, Madison, WI, USA) was added to each well for a total volume of 120 µL and incubated for 90 min at 25 °C. Absorbance at 490 nm (reference 630 nm) was measured using a PowerScan HT microplate reader (BioTek Instruments, Winooski, VT, USA). Data represent mean ± SEM from three technical replicates per condition.

### Cross-linking mass spectrometry analysis

C42 (final: 5, 10, and 20 µM) was mixed with 1 µM 70S ribosomes from the PUREfrex 1.0 kit (GeneFrontier Corporation, Japan) in 70S buffer (20 mM HEPES-KOH (pH 7.6), 100 mM KOAc, 10 mM Mg(OAc)_2_, 7 mM 2-mercaptoethanol) and incubated at 37 °C for 30 min. Then, DSSO crosslinker (Thermo Fisher Scientific, A33545) was added at a 200-fold molar excess, and the mixture was incubated for 1 h at room temperature. The reaction was quenched by adding Tris-HCl buffer (pH 7.5) to a final concentration of 20 mM. The cross-linked products were denatured by adding an equal amount of 2× PTS solution (200 mM Tris-HCl (pH 9.0), 24 mM sodium deoxycholate, 24 mM sodium *N*-lauroyl sarcosinate) and reduced by treatment with 10 mM DTT for 30 min at room temperature. The products were alkylated with 50 mM iodoacetamide for 30 min at room temperature in the dark. The products were then diluted five-fold with 50 mM NH_4_HCO_3_ and digested with Trypsin/Lys-C mix (Promega, V5072) at enzyme-to-protein ratio of 1:25 at 37 °C for 16 h. After the digestion, an equal volume of ethyl acetate and 1/20 volume of 10% trifluoroacetic acid (TFA) were added to the products, and the solution was mixed vigorously for 2 min. The mixture was centrifuged at 15,700 ×g for 2 min, and the lower aqueous phase was dried with a centrifugal evaporator after the upper ethyl acetate layer containing the surfactants was withdrawn. The peptides were re-dissolved in 0.1% TFA and 2% acetonitrile and desalted with a GL-tip SDB (GL Sciences, Japan).

nanoLC-ESI-MS/MS analysis was performed using a Q Exactive tandem mass spectrometer and an EASY-nLC1000 nanoflow HPLC system (Thermo Fisher Scientific). The trap column used for nanoHPLC was a 2 cm × 75 µm capillary column packed with 3 µm C18-silica particles (Thermo Fisher Scientific), and the separation column was a 12.5 cm × 75 µm capillary column packed with 3 µm C18-silica particles (Nikkyo Technos, Japan). The flow rate for nanoHPLC was 300 nL/min. The separation was conducted using a 10–40% linear acetonitrile gradient over 70 min in the presence of 0.1% formic acid. The nanoLC–MS/MS data were acquired in DDA mode controlled by the Xcalibur 4.0 software (Thermo Fisher Scientific). The DDA settings were as follows: the resolution was 70,000 for a full MS scan and 17,500 for an MS2 scan; the AGC target was 1 × 10^6^ for a full MS scan and 1 × 10^5^ for an MS2 scan; the maximum IT was 100 ms for a full MS and 60 ms for an MS2 scan; the full MS scan range was 310–1500 *m/z*; the loop count was 10; the isolation window was 2.0 *m/z*; the stepped NCE was set as 27 ± 6; the signals with a charge of +2 to +6 were included; the dynamic exclusion was set to 15 s. The cross-linked sites were identified using the XlinkX software bundled with Proteome Discoverer version 3.1 and illustrated using the UCSF ChimeraX package^102^. For structural visualization, cross-linked lysine residues were mapped onto the *E. coli* 70S ribosome structure (PDB ID: 6OFX)^103^. The amino acid sequences of *E. coli* ribosomal subunit proteins used for XlinkX searches were obtained from the UniProt database (downloaded on September 28, 2023). The mass spectrometry proteomics data were deposited to the ProteomeXchange Consortium via jPOST^104^.

### Statistics and reproducibility

Statistical analyses were performed using GraphPad Prism version 10.2.2 (GraphPad Software, Boston, MA, USA). Data are presented as mean ± SEM. Sample sizes, definitions of n, statistical tests, and details of data processing or model fitting are provided in the corresponding figure legends and relevant Methods sections. Sample sizes were not determined by statistical methods. No data were excluded from the analyses. Representative microscopy, gel, blot, and electron microscopy images show results that were reproduced in at least two independent experiments, unless otherwise stated.

### Reporting summary

Further information on research design is available in the Nature Portfolio Reporting Summary linked to this article.

## Supplementary information

Supplementary Information

Description of Additional Suppl. Files

Supplementary Data 1

Supplementary Data 2

Reporting Summary

Transparent Peer Review file

## Source data

Source Data

## Data Availability

All data supporting the findings of this study are available within the article, its Suppl. Information, and the repositories listed below. The mass spectrometry-based proteomics data generated in the TurboID experiments have been deposited in the ProteomeXchange Consortium via the PRIDE partner repository under accession code PXD072620 (https://www.ebi.ac.uk/pride/archive/projects/PXD072620). The processed TurboID proteomics data are provided in Suppl. Data 1. The XL-MS data have been deposited in the ProteomeXchange Consortium via the jPOST partner repository under accession codes PXD073821 and JPST004364 (https://repository.jpostdb.org/entry/JPST004364). The processed XL-MS data are provided in Suppl. Data 2. The negative-stain EM map has been deposited in the Electron Microscopy Data Bank (EMDB) under accession code EMD-68592 (https://www.ebi.ac.uk/emdb/EMD-68592). The microscopy image data have been deposited in figshare (https://doi.org/10.6084/m9.figshare.33364299)^105^. Source data are provided with this paper.
