# Supplementary material for: Antimicrobial peptides derived from Nemuri induce ribosomal condensation to inhibit translation
Source: Nat Commun. 2026 Oct 29;17:11037. doi: 10.1038/s41467-026-78960-5 (PMC13720017; doi:10.1038/s41467-026-78960-5)

Supplementary Information for:

Antimicrobial peptides derived from Nemuri induce ribosomal condensation to inhibit translation

Suguru Nishinami et al.

Correspondence: Nobuo N. Noda (nn@igm.hokudai.ac.jp) and
Hirofumi Toda (toda.hirofumi.gu@u.tsukuba.ac.jp)

Contents:

- Supplementary Figures 1–10
- Supplementary Tables 1–4
- Uncropped blots, gels, and plate images for the Supplementary Figures

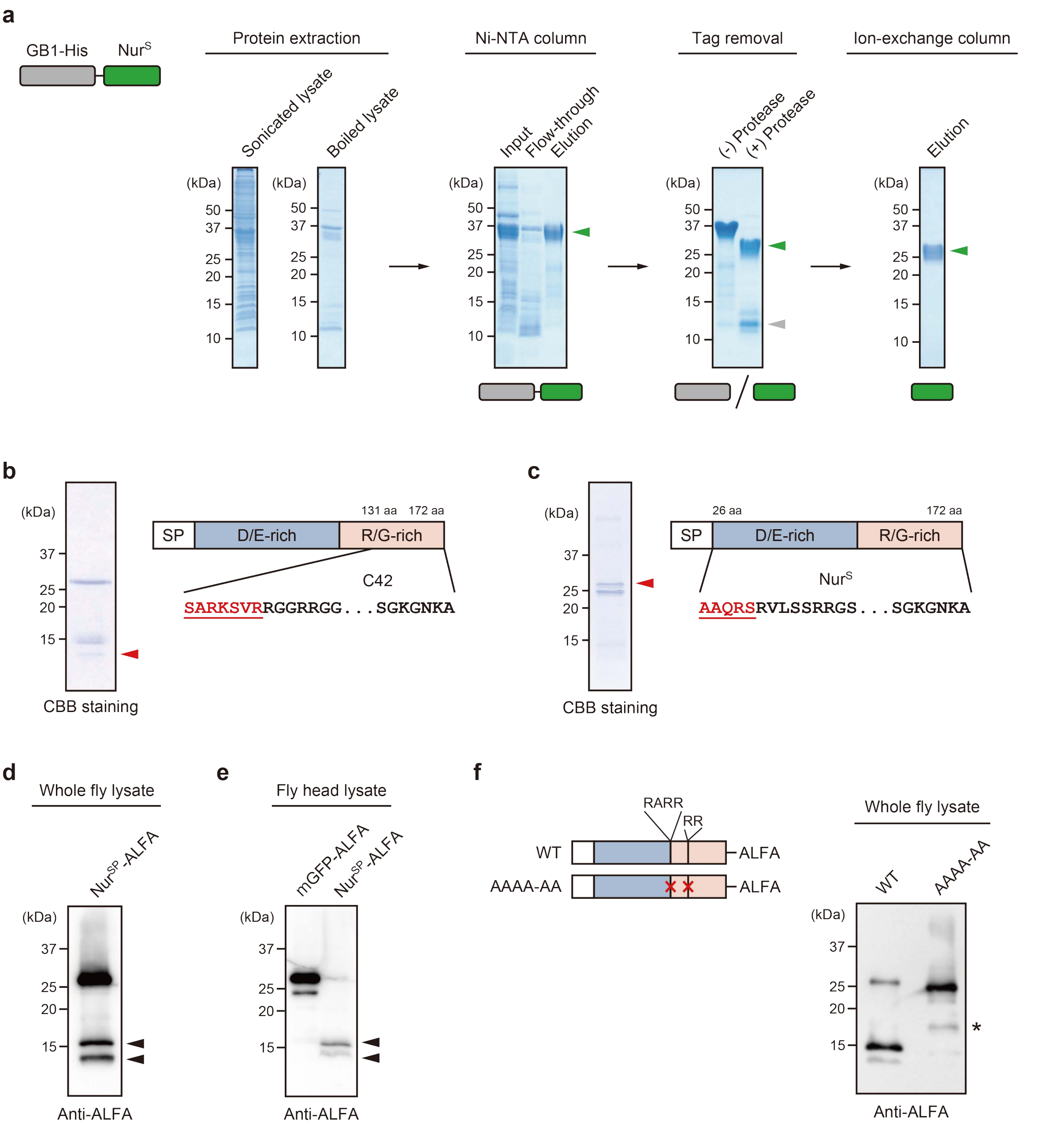

Supplementary Fig. 1. Identification of Nur processing products.

a, Purification of recombinant Nur protein lacking its signal peptide (Nur^S). *E. coli* expressing GB1-His-tagged Nur^S were lysed by sonication or boiling, and the boiled lysate was subjected to Ni-NTA affinity chromatography. Following tag removal, Nur^S was further purified using ion-exchange chromatography. SDS-PAGE was performed at each step. Arrowheads indicate Nur^S (green) and GB1-His tag (gray). **b, c**, Coomassie Brilliant Blue (CBB) staining of Nur products immunoprecipitated from the culture medium (**b**) or cell lysates (**c**) of S2 cells expressing ALFA-tagged Nur containing its signal peptide (Nur^{SP}-ALFA). Arrowheads indicate bands subjected to protein sequencing. The identified N-terminal sequences are underlined in red. **d**, Western blot of whole-fly lysates from flies expressing Nur^{SP}-ALFA under the control of the RU486-inducible Actin-GS driver. **e**, Western blot of head lysates from flies expressing Nur^{SP}-ALFA or mGFP-ALFA driven by nsyb-Gal4. Arrowheads in **d** and **e** indicate processed peptides. **f**, Western blot of whole-fly lysates from flies expressing Nur^{SP}-ALFA (WT) or a non-cleavable Nur mutant ($Nur^{AAAA\text{-}AA}$-ALFA) driven by nsyb-Gal4. In the mutant, the cleavage motifs RARR (107–110 aa) and RR (129–130 aa) were replaced with AAAA and AA, respectively. Asterisk indicates a nonspecific band.

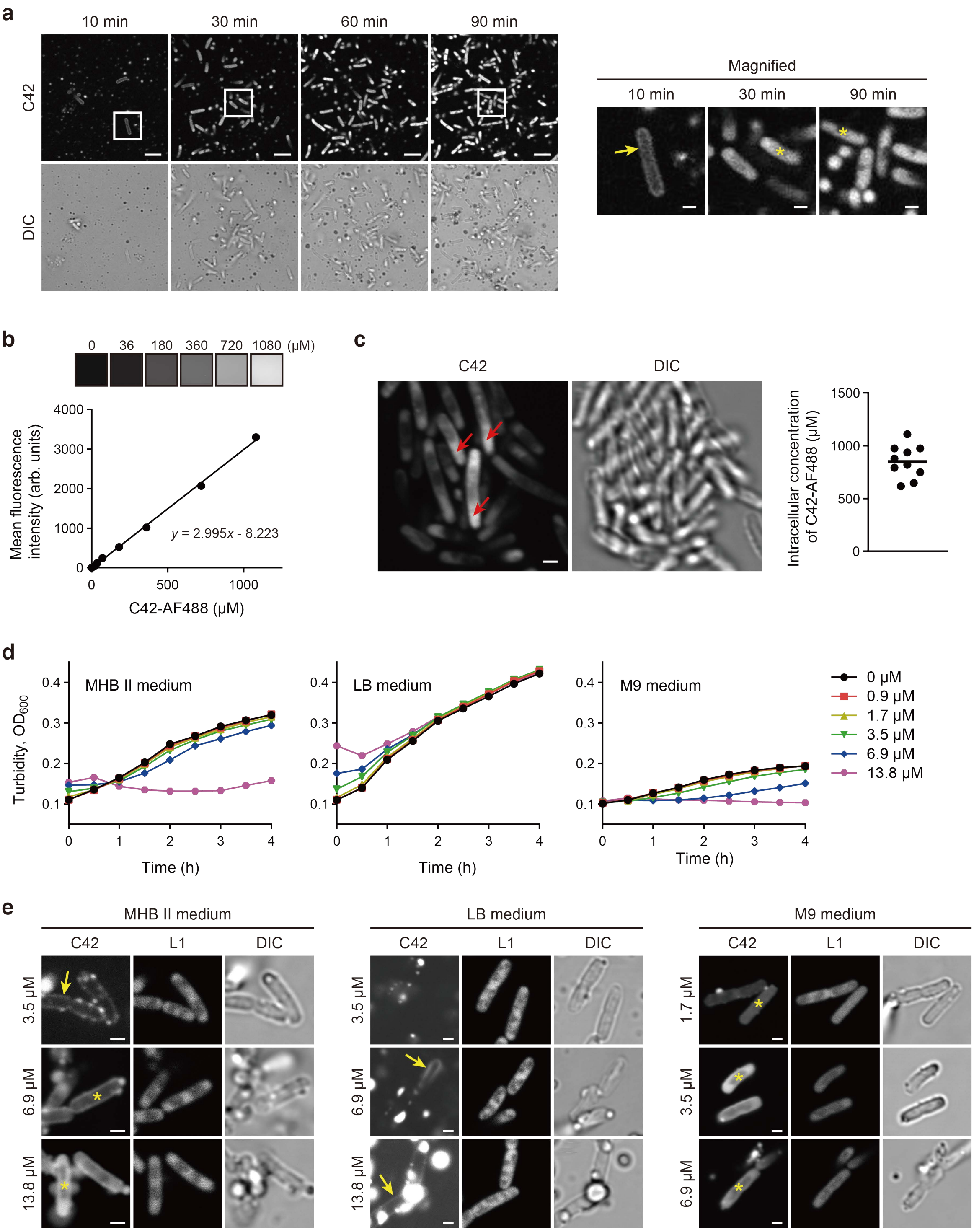

Supplementary Fig. 2. Bacterial uptake and antimicrobial activity of Nur peptides.

a, Confocal images of log-phase *E. coli* ATCC25922 incubated with 12 μM FITC-labeled C42 (C42-FITC) in MHB II medium. Arrow indicates membrane localization, and asterisks indicate intracellular localization of the peptide. Scale bars, 5 μm (main images); 1 μm (magnified images). **b**, Calibration images and calibration curve generated using defined concentrations of C42-AF488 in MHB II medium. Data are shown as mean ± SEM (n = 3 technical replicates). **c**, Representative confocal images of *E. coli* ATCC25922 treated with 36 μM C42-AF488 for 30–60 min in MHB II medium, with intracellular concentrations estimated from the calibration curve in **b**. Arrows indicate cells showing intracellular accumulation of C42-AF488. Dots represent individual cells (n = 10 cells); bars indicate mean ± SEM. Scale bars, 1 μm. **d**, Growth

curves of *E. coli* CJW7020 expressing L1-msfGFP in the presence of C42 in MHB II (left), LB (middle), or M9 medium (right). Data are shown as mean ± SEM (*n* = 2 independent experiments per condition). **e**, Confocal images of log-phase *E. coli* CJW7020 expressing L1-msfGFP incubated with the indicated concentrations of C42-TAMRA in MHB II (left), LB (middle), or M9 medium (right) for 30–60 min. Arrows indicate membrane localization. Asterisks indicate intracellular localization. Scale bars, 1 μm. Source data are provided as a Source Data file.

a

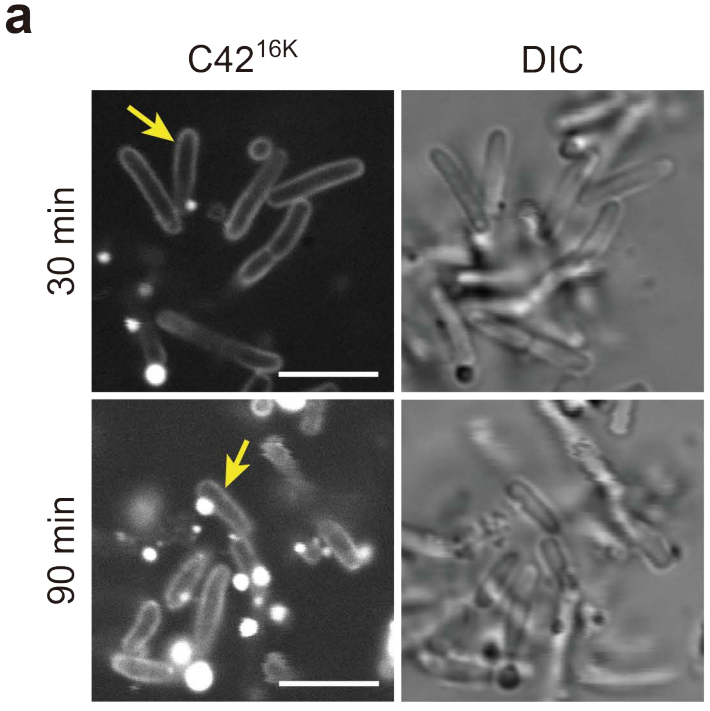

b

Test compound	Hemolytic concentration (μM)
Nur^S	>30
C42	>104

c

Test compound	MIC (μM)	
	E. coli BW25113	*E. coli* JW0368 (Δ*sbmA*)
Nur^S	>15	>15
C42	13	13

d

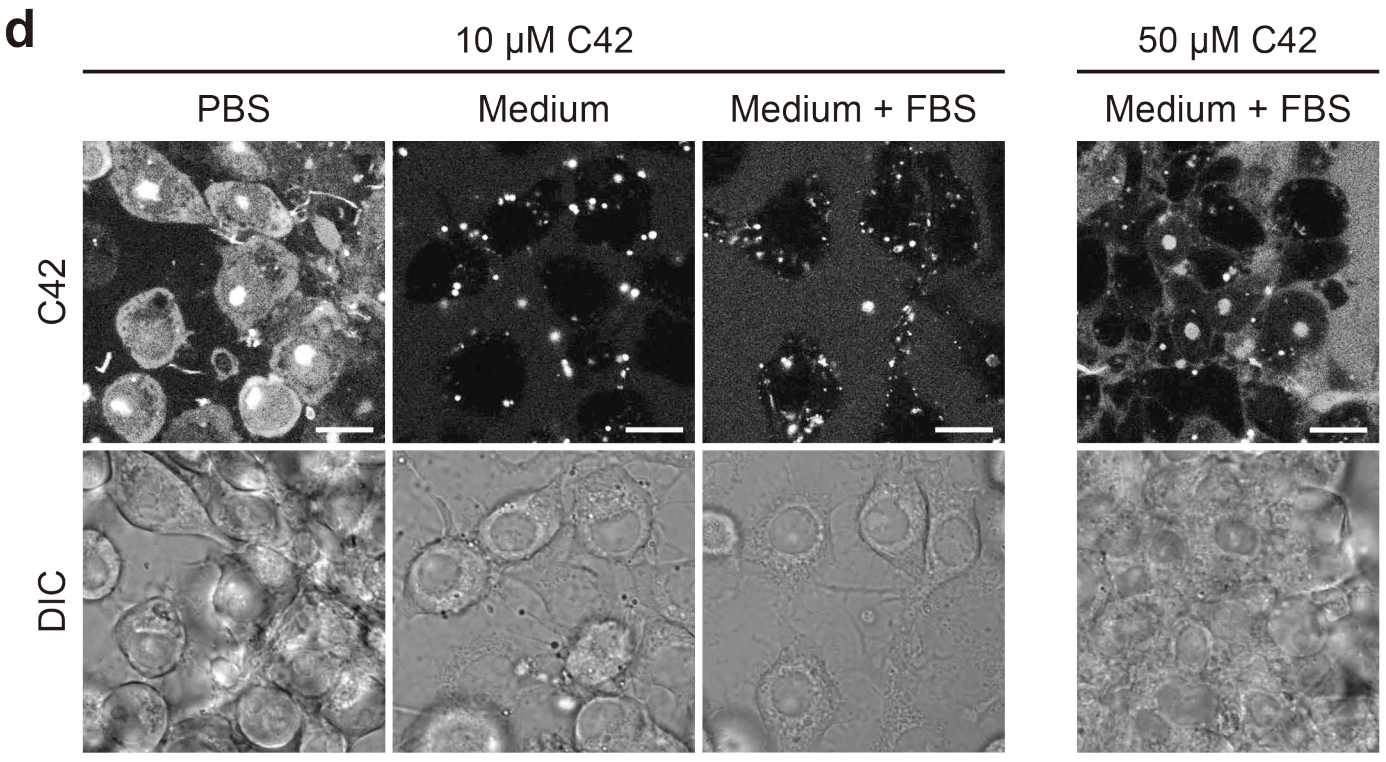

Supplementary Fig. 3. Membrane integrity and cell-selective uptake of Nur peptides.

a, Confocal images of log-phase *E. coli* ATCC25922 incubated with 14 μM C42^{16K}-FITC, a concentration at which C42^{WT}, but not C42^{16K}, exhibits antimicrobial activity. Images were taken after 30 min (top) and 90 min (bottom) of incubation. Arrows indicate membrane localization of the peptide. Scale bars, 5 μm. **b**, Hemolysis assay showing that Nur^S and C42 did not induce detectable hemolysis of sheep red blood cells at the concentrations tested. **c**, MICs of Nur^S and C42 against *E. coli* JW0368, which lacks the SbmA transporter. Strain BW25113 was used as the control. **d**, Confocal images of *Drosophila* S2R+ cells after a 1-h incubation with the indicated concentrations of C42-TAMRA. Before peptide treatment, S2R+ cells were transferred to the indicated media: Schneider's *Drosophila* medium, Schneider's *Drosophila* medium supplemented with 10% FBS, or PBS. Scale bars, 10 μm.

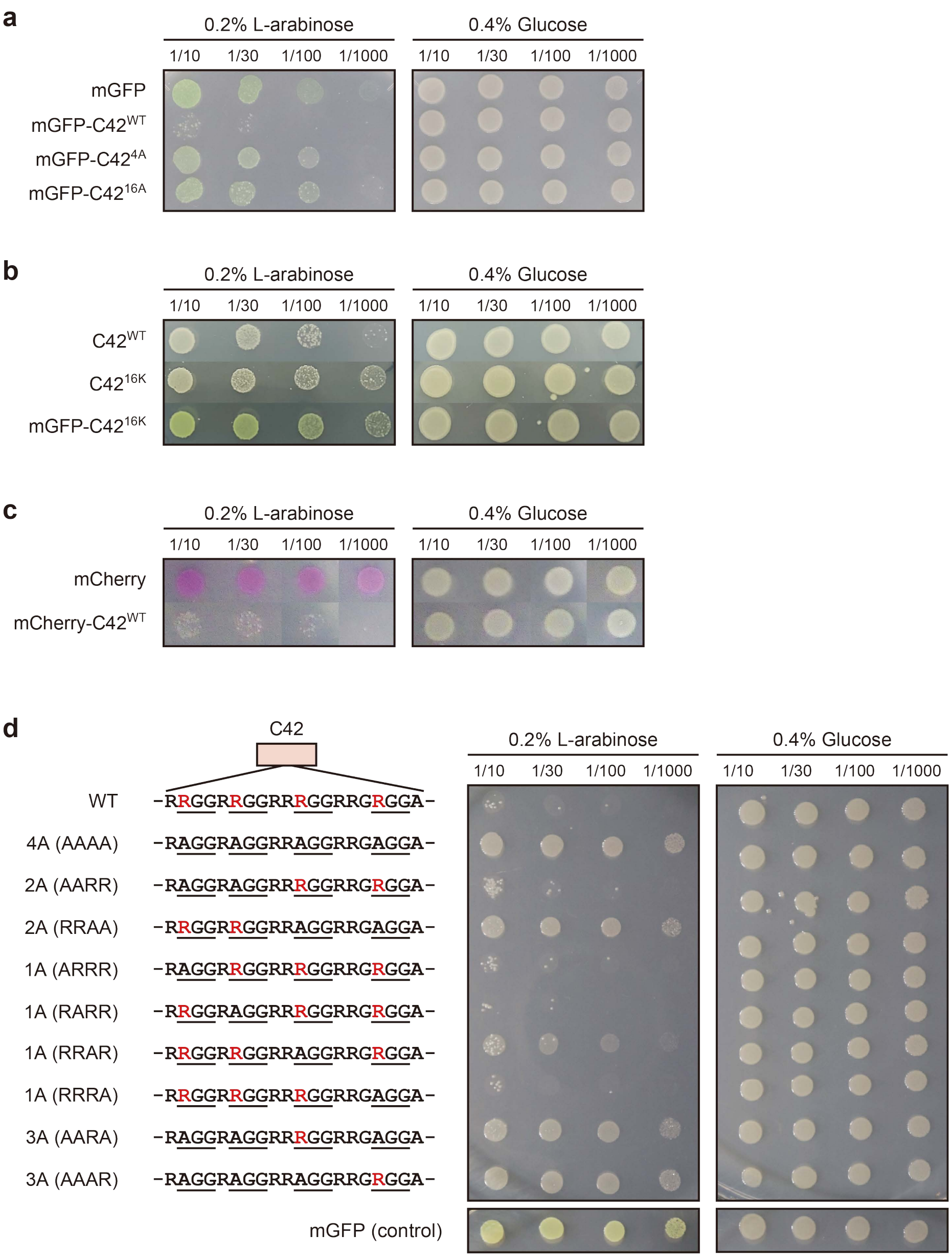

Supplementary Fig. 4. Antimicrobial activity of intracellularly expressed Nur peptides.

Spot assays of *E. coli* expressing C42 variants under the control of the pBAD promoter. **a**, Cells expressing mGFP-C42^{WT}, mGFP-C42^{4A}, mGFP-C42^{16A}, or mGFP alone. **b**, Cells expressing C42^{WT}, C42^{16K}, or mGFP-C42^{16K}. **c**, Cells expressing mCherry or mCherry-C42^{WT}. **d**, Cells expressing C42^{WT} or a series of arginine-substitution mutants of C42. mGFP was included as a control. In all panels, expression was induced with 0.2% L-arabinose (left), and 0.4% glucose served as a repression control (right).

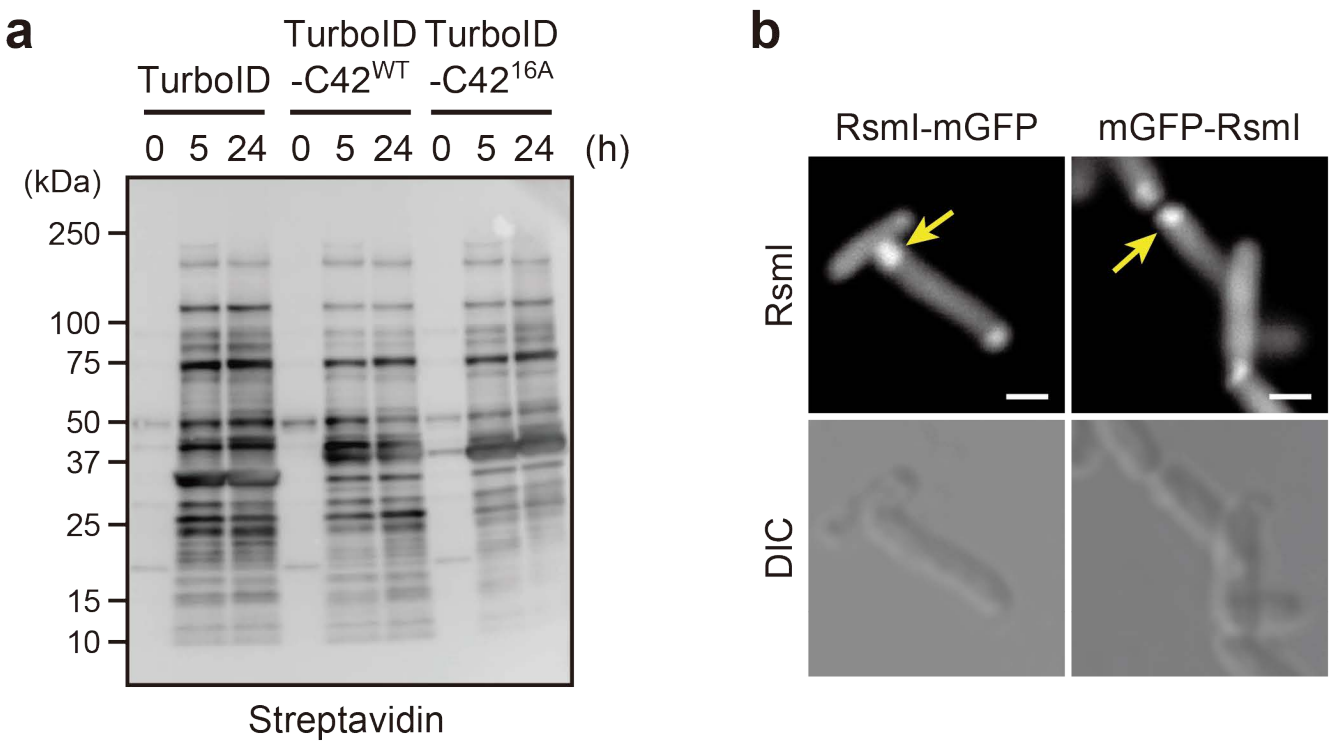

c

Test compound	MIC (μM) *Escherichia coli* BL21(DE3) Star
C62	4.5
C42	3.5
Amikacin	3.5

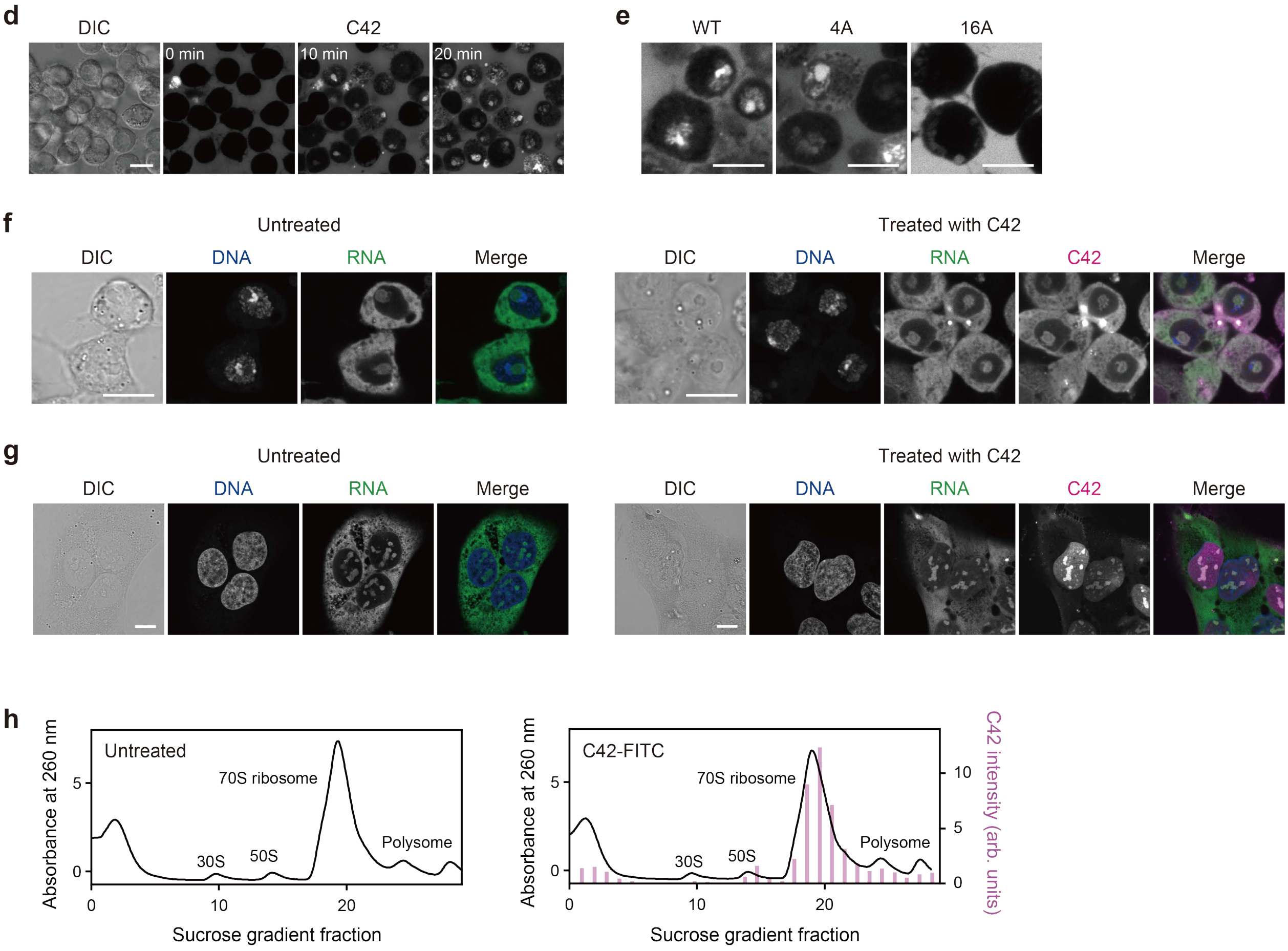

Supplementary Fig. 5. Association of Nur peptides with RNA- and ribosome-rich regions. **a**, Streptavidin blot of biotinylated proteins in lysates from *E. coli* expressing TurboID-C42 variants (WT or 16A) or TurboID alone from the L-arabinose-inducible pBAD promoter. Cells were collected at 0, 5, and 24 h after biotin addition. **b**, Confocal images of *E. coli* expressing RsmI-mGFP (left) or mGFP-RsmI (right) from the L-arabinose-inducible pBAD promoter. Arrows indicate GFP foci. Scale bars, 1 µm. **c**, MICs of C62 and C42 against *E. coli* BL21(DE3) Star. Amikacin was included as a control. **d–f**, Confocal images of *Drosophila* S2R+ cells in serum-free Schneider's *Drosophila* medium, showing time-lapse live imaging after addition of 25 µM C42-FITC (**d**), comparison of 25 µM C42-FITC (WT, 4A, and 16A) after 30 min (**e**), and cells incubated without or with 25 µM C42-FITC (magenta) for 30 min, followed by fixation and co-staining with Hoechst for DNA (blue) and Nucleolus Bright Red for RNA (green) (**f**). Scale bars, 10 µm. **g**, Confocal images of human U2OS cells incubated without or with 25 µM C42-FITC (magenta) in PBS for 30 min, followed by fixation and co-staining with DAPI for DNA (blue) and Nucleolus Bright Red for RNA (green). Scale bars, 10

µm. **h**, Sucrose density gradient profiles of *E. coli* MRE600 lysates incubated without (left) or with (right) C42-FITC, showing 260-nm absorbance (black; left *y*-axis) and C42-FITC fluorescence (magenta; right *y*-axis) against fraction number (*x*-axis). Source data are provided as a Source Data file.

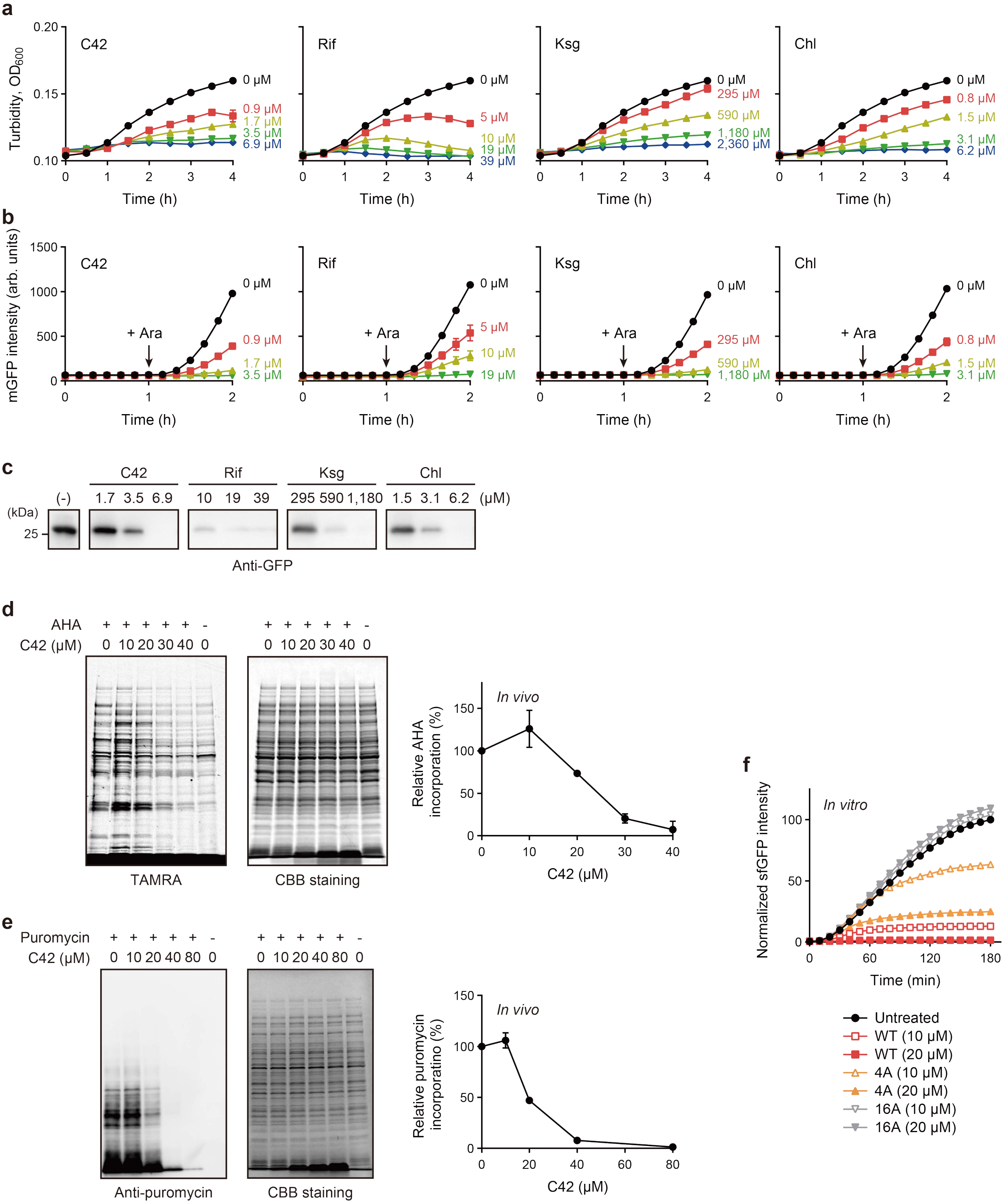

Supplementary Fig. 6. Translation inhibition by Nur peptides.

a, b, Growth curves (**a**) and mGFP fluorescence levels (**b**) of *E. coli* carrying the pBAD-mGFP plasmid in the absence or presence of C42, rifampicin (Rif), kasugamycin (Ksg), or chloramphenicol (Chl). In **b**, the compounds were added 1 h before L-arabinose (Ara), as indicated by the arrows. Data are shown as mean ± SEM (**a**, n = 3 independent cultures per condition; **b**, n = 3, 10, 3, and 9 independent cultures for C42, Rif, Ksg, and Chl, respectively). **c**, Western blot of lysates from the samples in **b**, probed with an anti-GFP antibody. The minus sign indicates the untreated control. **d, e**, Global nascent protein synthesis measured by L-azidohomoalanine (AHA) metabolic labeling (**d**) or puromycin incorporation (**e**). *E. coli* cells

were treated with C42 and then labeled with AHA or puromycin. AHA-containing proteins were detected by click labeling with TAMRA alkyne, whereas puromycin incorporation was detected by immunoblotting with an anti-puromycin antibody. Total protein loading was assessed by Coomassie Brilliant Blue (CBB) staining. TAMRA fluorescence (**d**) and puromycin signals (**e**) were normalized to the corresponding CBB total protein signals. For background correction, the CBB-normalized no-AHA and no-puromycin control values were subtracted from the corresponding sample values in **d** and **e**, respectively. Values were expressed relative to the no-C42 control (set to 100%). Data are shown as mean ± SEM (n = 2 independent experiments per condition). **f**, *In vitro* translation inhibition assay measuring sfGFP production in the presence of C42 variants (WT, 4A, or 16A; red, orange, and gray, respectively) at the indicated concentrations. Data are shown as mean ± SEM (n = 5 independent experiments per condition). Source data are provided as a Source Data file.

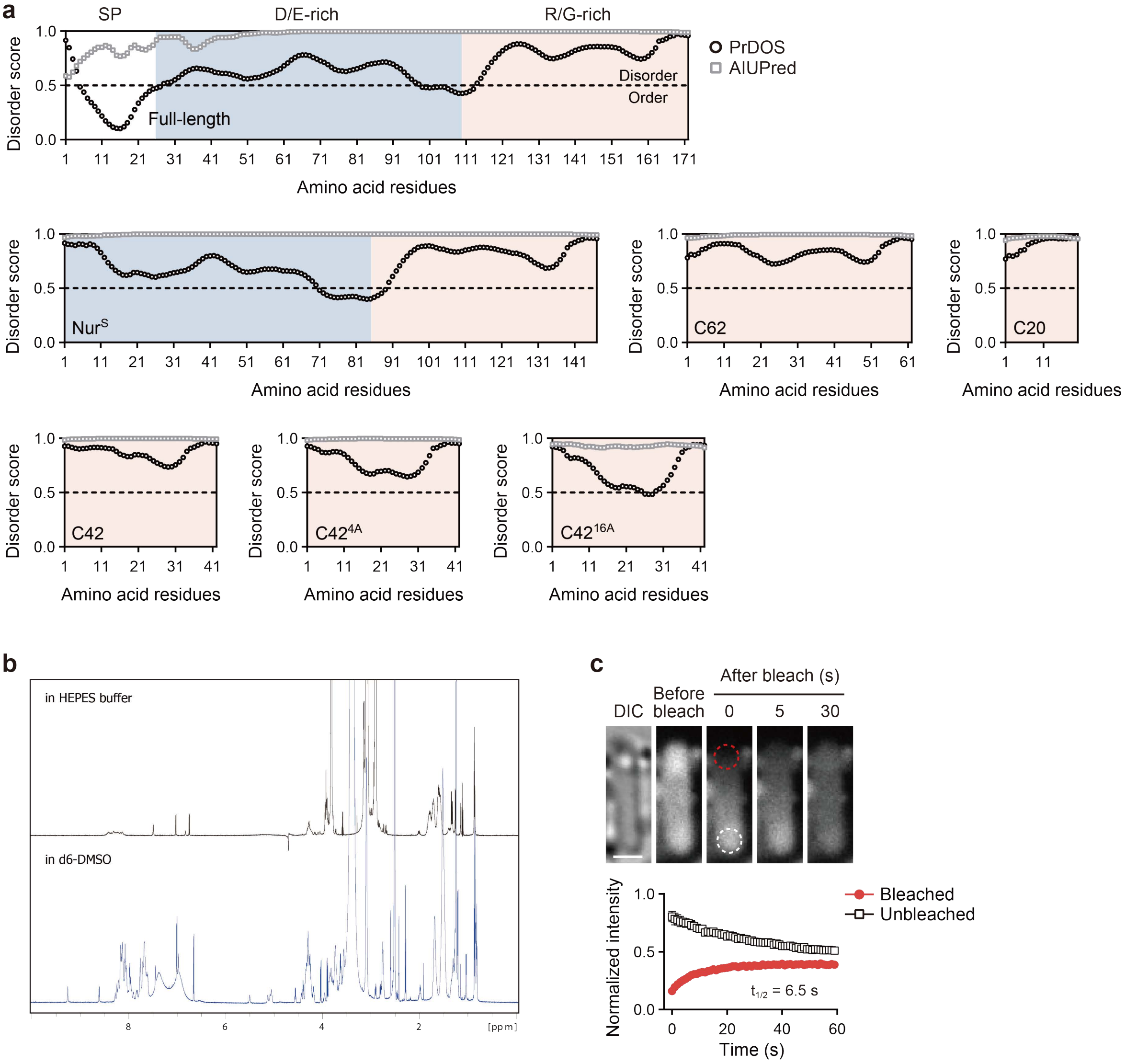

Supplementary Fig. 7. Biophysical properties of Nur peptides.

a, Intrinsic disorder predictions of Nur and its peptides by PrDOS (black circles) and AIUPred (gray squares): full-length Nur (1–172 aa), Nur^{S} (26–172 aa), C62 (111–172 aa), C20 (111–130 aa), and C42 variants (WT, 4A, and 16A; 131–172 aa). Regions shaded white, blue, and red indicate the signal peptide (SP), D/E-rich regions, and R/G-rich regions, respectively. **b**, 1D ^{1}H NMR spectra of C42 in HEPES buffer (top) and DMSO (bottom). **c**, FRAP analysis of log-phase *E. coli* ATCC25922 after a 30-min incubation with 12 μM C42-FITC in MHB II medium. Red and white dotted circles indicate the photobleached and unbleached regions, respectively. The graph shows normalized C42-FITC fluorescence in the photobleached regions (red circles) and unbleached regions (open squares). The estimated fluorescence recovery half-time ($t_{1/2}$) was 6.5 s. Data are shown as mean ± SEM (n = 3 cells). Scale bar, 1 μm. Source data are provided as a Source Data file.

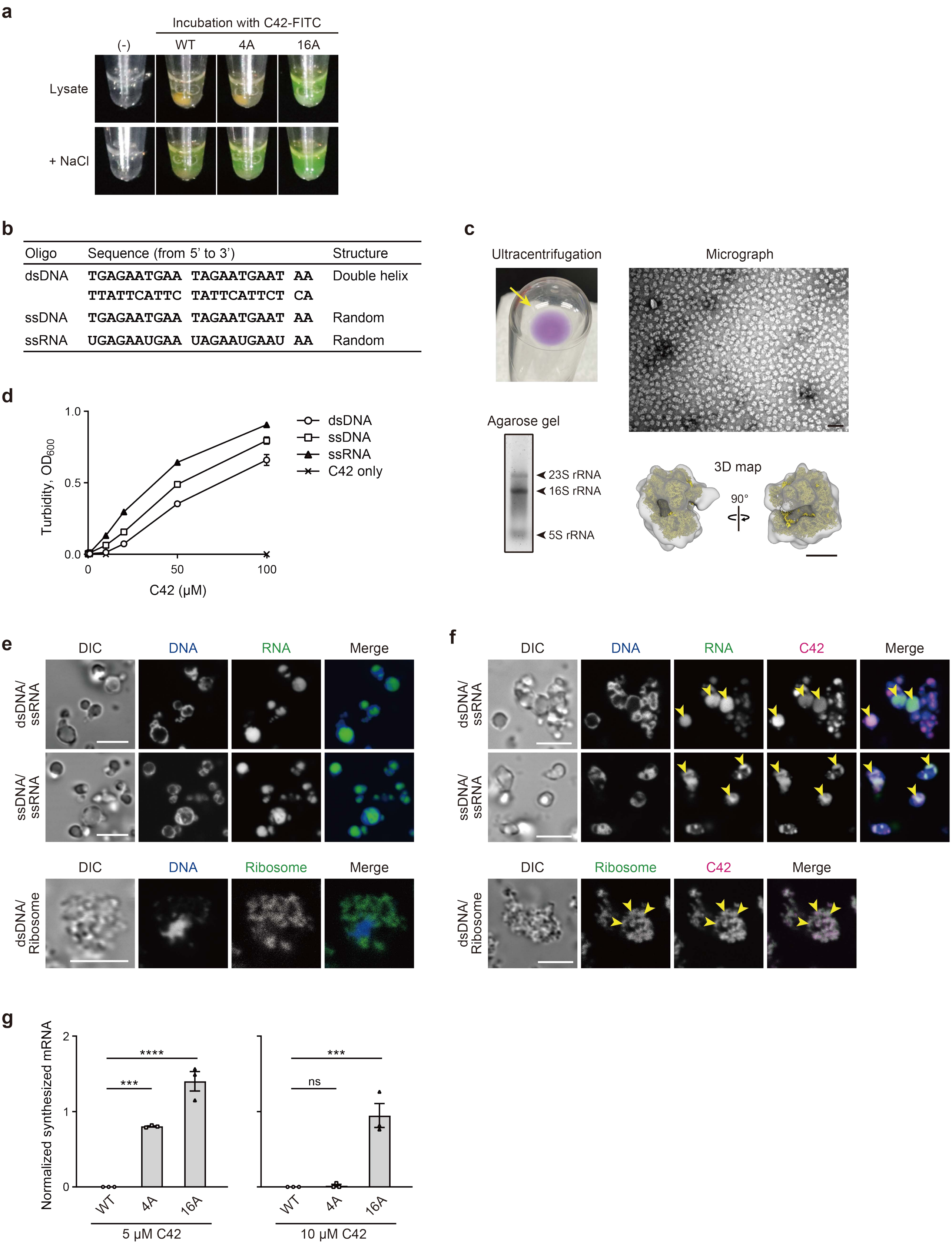

Supplementary Fig. 8. *In vitro* condensation induced by Nur peptides.

a, Images of 50% (v/v) *E. coli* lysate incubated with C42-FITC variants (WT, 4A, or 16A) in the absence (top) or presence (bottom) of 500 mM NaCl. The minus sign indicates the no-peptide control. **b**, Sequences and structures of oligonucleotides used in this study. **c**, Characterization of ribosomes purified from *E. coli* QC101 expressing L9-mCherry.

Ribosomes were pelleted by ultracentrifugation (arrow). The integrity of ribosomal RNA was confirmed by agarose gel electrophoresis. The quality of the purified ribosome particles was assessed by negative-stain electron microscopy. A representative micrograph is shown. The reconstructed 3D map (gray) is compared with the reference map (EMD-15793; yellow). Scale bars, 50 nm (micrograph); 10 nm (3D map). **d**, Turbidity of C42 alone or mixed with dsDNA, ssDNA, or ssRNA (each at 5 mM charge) at the indicated C42 concentrations. Data are shown as mean ± SEM ($n = 3$ independently prepared samples per condition). **e**, Confocal images of *in vitro* reconstituted multiphase condensates containing poly-L-lysine (PLL), DNA, and either RNA or ribosomes. Oligonucleotide DNA (dsDNA or ssDNA; blue), ssRNA or ribosomes (green), and PLL were used as indicated. Scale bars, 1 µm. **f**, Localization of TAMRA- or FITC-labeled C42 (magenta) within *in vitro* reconstituted multiphase condensates containing PLL, DNA (blue), and either RNA or ribosomes (green). Arrowheads indicate regions enriched in both C42 and RNA or ribosomes. Scale bars, 5 µm. **g**, *In vitro* transcription in the absence or presence of 5 µM (left) or 10 µM (right) C42 (WT, 4A, or 16A). mRNA yields were normalized to the untreated control (set to 1), with undetectable values set to 0. Data are shown as mean ± SEM ($n = 3$ independently prepared reactions per condition). At each concentration, differences among the C42 variants were assessed using one-way ANOVA followed by Dunnett's multiple-comparisons test, with WT as the control (****: $P < 0.0001$, ***: $P < 0.001$, ns: $P \geq 0.05$). Source data are provided as a Source Data file.

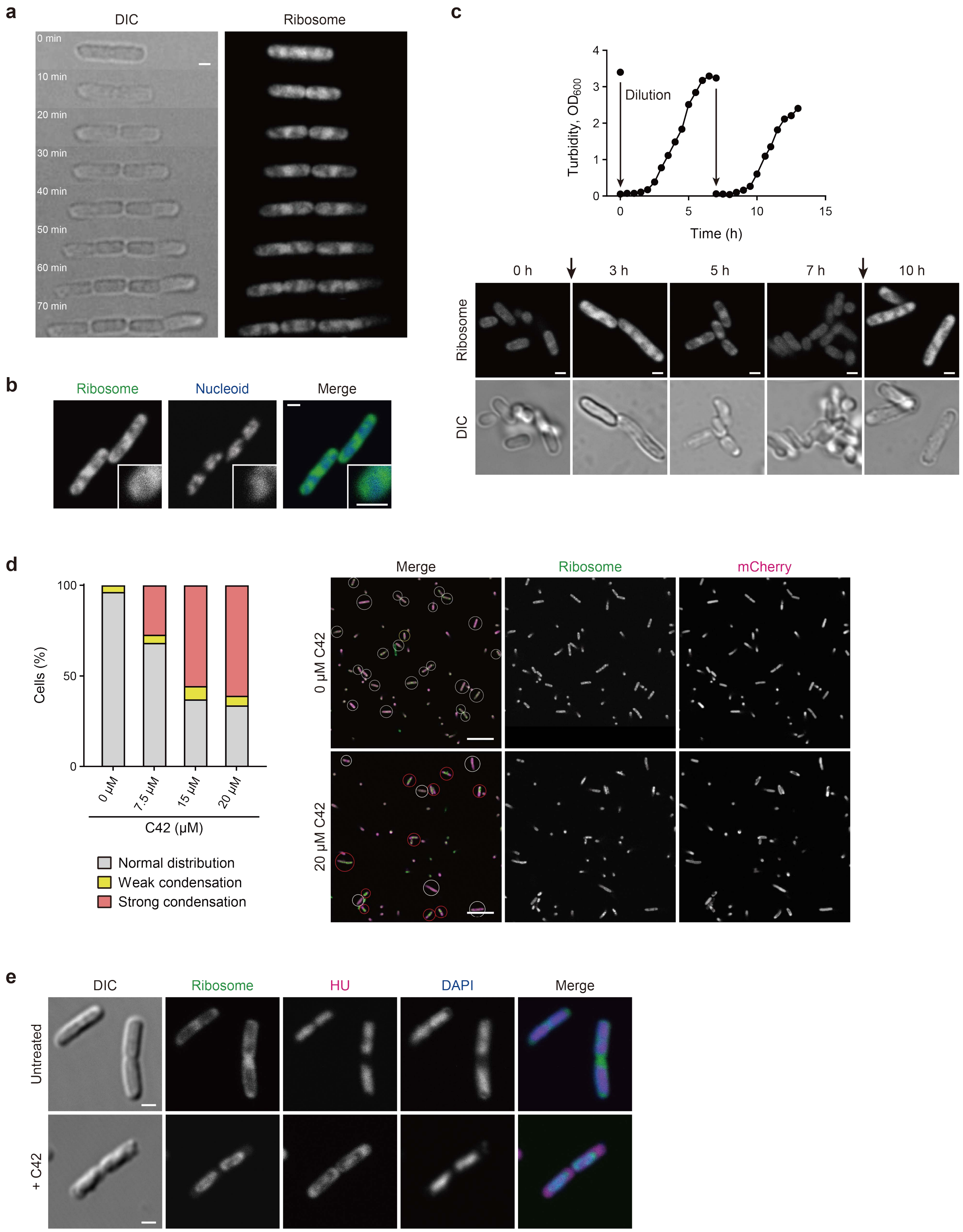

Supplementary Fig. 9. Spatial organization of ribosome-rich regions in *E. coli*.

a, Time-lapse confocal images of *E. coli* CJW7020 expressing L1-msfGFP as a ribosomal marker during cell division in LB medium. Scale bar, 1 μm. **b**, Confocal images of log-phase *E. coli* expressing L1-msfGFP (ribosomes, green) and HU-mCherry (nucleoids, blue) in LB medium. Insets show magnified regions. Scale bars, 1 μm. **c**, Growth curve and confocal images of *E. coli* CJW7020 expressing L1-msfGFP in LB medium at the indicated culture

times. Arrows indicate two successive 1:1,000 culture dilutions. Turbidity is shown as mean ± SEM (n = 3 technical replicates). Scale bars, 1 µm. **d**, Confocal images and quantification of *E. coli* expressing L1-msfGFP (ribosomes, green) and L-arabinose-induced cytoplasmic mCherry (magenta) after treatment with C42 at the indicated concentrations in M9 medium. Cells were classified as showing normal L1-msfGFP distribution (gray), weak condensation (yellow), or strong condensation (red). Colored outlines indicate the corresponding categories. The stacked bar graph shows the percentage of cells in each category. A total of 107, 66, 97, and 92 cells were analyzed for 0, 7.5, 15, and 20 µM C42, respectively. Scale bars, 10 µm. **e**, Simultaneous imaging of *E. coli* expressing L1-msfGFP (ribosomes, green) and HU-mCherry (nucleoids, magenta) and stained with DAPI (DNA, blue) after treatment without (top) or with (bottom) 13 µM C42 in M9 medium. Scale bars, 1 µm. Source data are provided as a Source Data file.

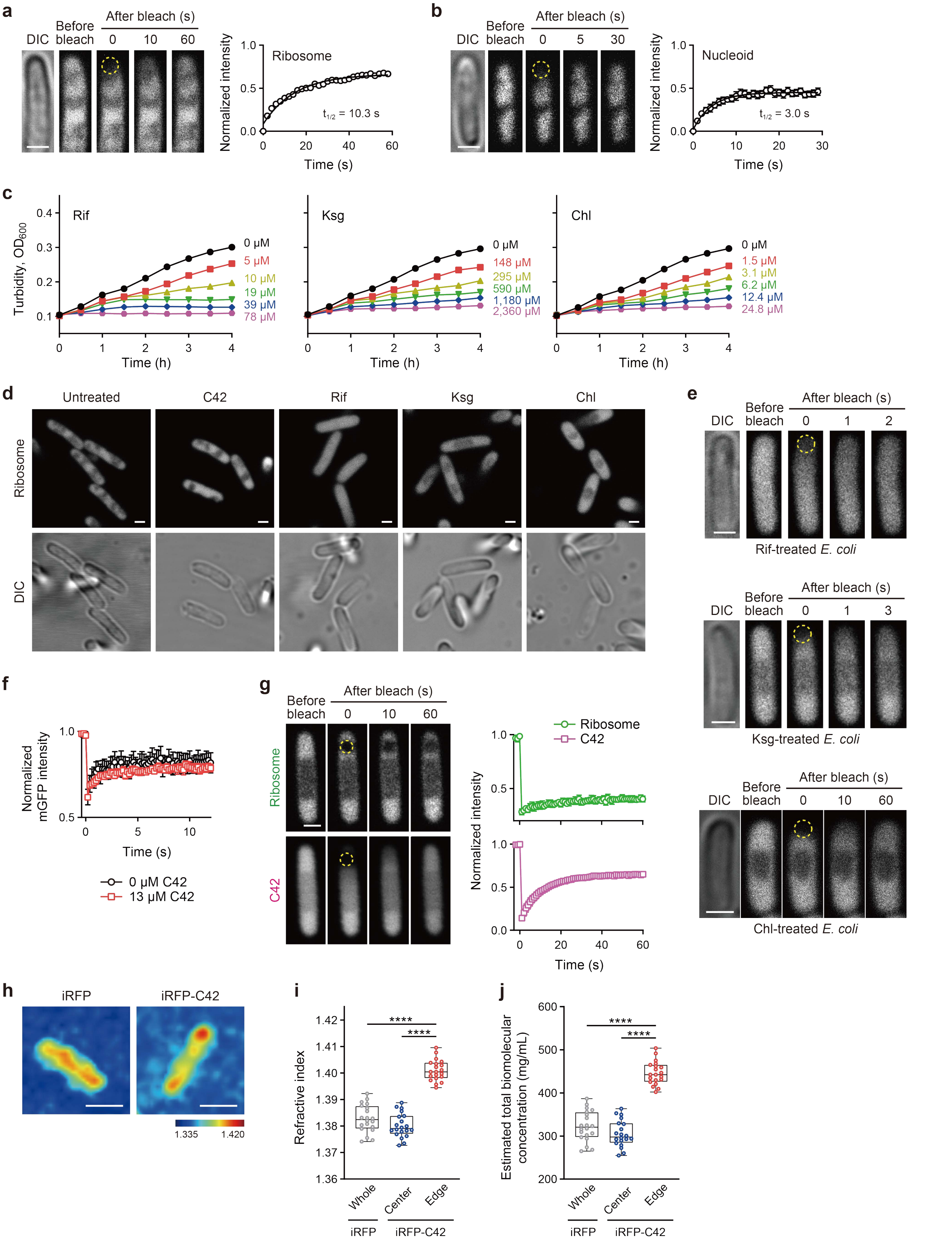

Supplementary Fig. 10. Nur peptide-induced changes in ribosomal mobility and biomolecular distribution in *E. coli*.

a, b, FRAP of log-phase *E. coli* expressing L1-msfGFP (**a**) or HU-mCherry (**b**). Curves show normalized fluorescence (prebleach = 1; postbleach = 0; mean ± SEM; *n* = 10 cells per panel). Recovery half-times ($t_{1/2}$) are shown. Scale bars, 1 µm. **c**, Growth curves of *E. coli* CJW7020 expressing L1-msfGFP with the indicated concentrations of rifampicin (Rif; left), kasugamycin (Ksg; middle), or chloramphenicol (Chl; right) in MHB II medium. Data are shown as mean ± SEM (*n* = 3 independent experiments per condition). **d**, Confocal images of *E. coli* expressing L1-msfGFP untreated or treated for 1 h with C42, Rif, Ksg, or Chl (13, 156, 4,721, and 50 µM, respectively). Scale bars, 1 µm. **e**, FRAP of *E. coli* CJW7020 expressing L1-msfGFP after the antibiotic treatments in **d**. Scale bars, 1 µm. **f**, FRAP of *E. coli* expressing L-arabinose-induced soluble mGFP without or with 13 µM C42 in M9 medium. Curves show normalized fluorescence (mean ± SEM; *n* = 3 untreated and 5 C42-treated cells). **g**, FRAP of *E. coli* expressing L1-msfGFP (green) and pBAD-driven mCherry-C42 (magenta) after 15 h of L-arabinose induction in LB medium. Curves show normalized fluorescence (mean ± SEM; *n* = 3 cells). Scale bar, 1 µm. Dotted circles in **a**, **b**, **e**, and **g** indicate photobleached regions. **h**, Refractive index (RI) images of *E. coli* expressing iRFP (left) or iRFP-C42 (right). The color scale indicates RI. Scale bars, 1 µm. **i**, Cellular RI quantified from **h** across whole-cell regions of iRFP-expressing cells (*n* = 20 cells) and at the center and edge of iRFP-C42-expressing cells (*n* = 20 cells). **j**, Total biomolecular concentrations estimated from **i**. Box plots in **i** and **j** show medians and interquartile ranges, with whiskers extending to the minimum and maximum values and dots representing individual measurements. Differences were assessed using one-way ANOVA followed by Dunnett's multiple-comparisons test, with the edge region of iRFP-C42-expressing cells as the control (****: $P < 0.0001$). Source data are provided as a Source Data file.

Supplementary Table 1. MIC values of Nur peptides against a broad range of microbes.

Class	Species	Strain	MIC (μM)										
			C20	C42	C62	Amikacin	Amphotericin	Vancomycin	Clarithromycin	Rifampicin	Meropenem	CPFX	Colistin
Gram-negative bacteria	*Escherichia coli*	K-12	16	8	4	2	N.T.	N.T.	N.T.	N.T.	N.T.	N.T.	N.T.
	Escherichia coli	K-12 Δ tolC	8	8	4	1	N.T.	N.T.	N.T.	N.T.	N.T.	N.T.	N.T.
	Escherichia coli	K-12 lptD4213	4	2	2	0.125	N.T.	N.T.	N.T.	N.T.	N.T.	N.T.	N.T.
	Escherichia coli	ATCC BAA-2452 (NDM-1)	16	8	4	>64	N.T.	N.T.	N.T.	N.T.	N.T.	N.T.	N.T.
	Salmonella enteritidis	1891	32	8	4	8	N.T.	N.T.	N.T.	N.T.	N.T.	N.T.	N.T.
	Pseudomonas aeruginosa	A3	>64	32	8	0.3	N.T.	N.T.	N.T.	N.T.	0.1	0.2	2.7
	Pseudomonas aeruginosa	PAO1	>64	64	8	0.6	N.T.	N.T.	N.T.	N.T.	0.9	0.4	2.7
	Pseudomonas aeruginosa	GTC14657 MDRP (blaVIM-2)	>64	32	16	37.5	N.T.	N.T.	N.T.	N.T.	56	12	2.7
	Pseudomonas aeruginosa	GTC14660 MDRP (bla$_{IMP-1}$)	>64	>64	8	75.0	N.T.	N.T.	N.T.	N.T.	>56	12	2.7
	Pseudomonas aeruginosa	GTC14659 MDRP (bla$_{IMP-1}$, AAC(6'))	>64	64	8	9.4	N.T.	N.T.	N.T.	N.T.	>56	96.6	2.7
	Klebsiella pneumoniae	PCI602	32	8	4	2	N.T.	N.T.	N.T.	N.T.	N.T.	N.T.	N.T.
	Klebsiella pneumoniae	ATCC BAA-1705 (KPC-2)	64	32	8	32	N.T.	N.T.	N.T.	N.T.	N.T.	N.T.	N.T.
	Acinetobacter baumannii	ATCC19606	32	8	4	16	N.T.	N.T.	N.T.	N.T.	N.T.	N.T.	N.T.
	Acinetobacter baumannii	ATCC BAA-1710	32	8	8	64	N.T.	N.T.	N.T.	N.T.	N.T.	N.T.	N.T.
	Serratia marcescens	B-0524	>64	>32	>32	1	N.T.	N.T.	N.T.	N.T.	N.T.	N.T.	N.T.
	Serratia marcescens	ATCC14756	>64	>32	>32	0.5	N.T.	N.T.	N.T.	N.T.	N.T.	N.T.	N.T.
	Serratia marcescens	JCM20081 (IAM1161)	>64	>32	>32	0.5	N.T.	N.T.	N.T.	N.T.	N.T.	N.T.	N.T.
	Serratia marcescens	JCM20091 (IAM1184)	>64	>32	>32	1	N.T.	N.T.	N.T.	N.T.	N.T.	N.T.	N.T.
	Serratia marcescens	IFO3046	>64	>32	>32	0.5	N.T.	N.T.	N.T.	N.T.	N.T.	N.T.	N.T.
Gram-positive bacteria	*Staphylococcus aureus*	FDA209P	8	4	2	1	N.T.	N.T.	N.T.	N.T.	N.T.	N.T.	N.T.
	Staphylococcus aureus	Mut50 (VISA/MRSA)	32	8	4	16	N.T.	N.T.	N.T.	N.T.	N.T.	N.T.	N.T.
	Streptococcus pyogenes	MH750	16	4	4	2	N.T.	N.T.	N.T.	N.T.	N.T.	N.T.	N.T.
	Streptococcus pyogenes	MH759 (ermB)	16	4	4	2	N.T.	N.T.	N.T.	N.T.	N.T.	N.T.	N.T.
	Enterococcus faecalis	JCM5803	>64	32	8	N.T.	N.T.	0.25	N.T.	N.T.	N.T.	N.T.	N.T.
	Enterococcus faecalis	NCTC12201 (VRE)	>64	32	16	N.T.	N.T.	>64	N.T.	N.T.	N.T.	N.T.	N.T.
Mycobacteria	*Mycobacterium smegmatis*	ATCC607	>64	4	4	N.T.	N.T.	N.T.	0.25	4	N.T.	N.T.	N.T.
	Mycobacterium abscessus	JCM13569	>64	>32	>32	N.T.	N.T.	N.T.	0.5	8	N.T.	N.T.	N.T.
	Mycobacterium avium	ATCC25291	>64	>32	>32	N.T.	N.T.	N.T.	0.25	2	N.T.	N.T.	N.T.
	Mycobacterium intracellulare	JCM6384	>64	32	32	N.T.	N.T.	N.T.	0.0625	0.0078	N.T.	N.T.	N.T.
Yeast	*Candida albicans*	TIMM 3169 (QC)	32	32	16	N.T.	<0.0625	N.T.	N.T.	N.T.	N.T.	N.T.	N.T.

Supplementary Table 2. Comparison of MIC and MBC values of Nur peptides.

Species	Measurement	C20	C42	C62	Chloramphenicol	Amikacin
S. aureus (FDA209P)	MIC (µM)	8	4	2	2	0.5
	MBC (µM)	8	4	4	>8	1
	MBC/MIC	1	1	2	>4	2
E. coli (K12)	MIC (µM)	16	4	4	1	1
	MBC (µM)	16	4	4	>4	1
	MBC/MIC	1	1	1	>4	1

Supplementary Table 3. Plasmids used in this study.

Name	System	Experiment in this study	Vector	Cloning detail	Template
pET22b-sfGFP	*In vitro*	cell-free RNA synthesis	pET22b	Previous work (Tomono et al., 2024)	-
pGBHPS-Nur^{S}-YC	*E. coli*	protein expression and purification	pGBHPS	NEBuilder HiFi DNA Assembly Cloning Kit	nur wt
pBAD-C42^{WT}	*E. coli*	spot assay	pBAD	Restriction ligation (NdeI/EcoRI)	nur wt
pBAD-C42^{4A}	*E. coli*	spot assay	pBAD	Restriction ligation (NdeI/EcoRI)	nur AAAA mutant
pBAD-C42^{16A}	*E. coli*	spot assay	pBAD	Restriction ligation (NdeI/EcoRI)	nur (16A) mutant
pBAD-C42^{16K}	*E. coli*	spot assay	pBAD	Restriction ligation (NdeI/EcoRI)	nur (16K) mutant
pBAD-C42$^{3A(AARA)}$	*E. coli*	spot assay	pBAD	Restriction ligation (NdeI/EcoRI)	nur AARR mutant
pBAD-C42$^{3A(AAAR)}$	*E. coli*	spot assay	pBAD	Restriction ligation (NdeI/EcoRI)	nur AAAR mutant
pBAD-C42$^{2A(AARR)}$	*E. coli*	spot assay	pBAD	Restriction ligation (NdeI/EcoRI)	nur AARR mutant
pBAD-C42$^{2A(RRAA)}$	*E. coli*	spot assay	pBAD	Restriction ligation (NdeI/EcoRI)	nur RRAA mutant
pBAD-C42$^{1A(ARRR)}$	*E. coli*	spot assay	pBAD	Restriction ligation (NdeI/EcoRI)	nur ARRR mutant
pBAD-C42$^{1A(RARR)}$	*E. coli*	spot assay	pBAD	Restriction ligation (NdeI/EcoRI)	nur RARR mutant
pBAD-C42$^{1A(RRAR)}$	*E. coli*	spot assay	pBAD	Restriction ligation (NdeI/EcoRI)	nur RRAR mutant
pBAD-C42$^{1A(RRRA)}$	*E. coli*	spot assay	pBAD	Restriction ligation (NdeI/EcoRI)	nur RRRA mutant
pBAD-mCherry	*E. coli*	microscopic imaging	pBAD	Restriction ligation (NdeI/EcoRI)	mCherry
pBAD-mCherry-C42^{WT}	*E. coli*	microscopic imaging	pBAD	Restriction ligation (NdeI/EcoRI)	mCherry tagged with nur 42 wt
pBAD-mGFP	*E. coli*	spot assay, transcription and translation assay	pBAD	Restriction ligation (NdeI/EcoRI)	mGFP
pBAD-mGFP-C42^{WT}	*E. coli*	microscopic imaging	pBAD	Restriction ligation (NdeI/EcoRI)	mGFP tagged with nur 42 wt
pBAD-mGFP-C42^{4A}	*E. coli*	microscopic imaging	pBAD	Restriction ligation (NdeI/EcoRI)	mGFP tagged with nur 42 AAAA mutant
pBAD-mGFP-C42^{16A}	*E. coli*	microscopic imaging	pBAD	Restriction ligation (NdeI/EcoRI)	mGFP tagged with nur 42 (16A) mutant
pBAD-mGFP-C42^{16K}	*E. coli*	microscopic imaging	pBAD	Restriction ligation (NdeI/EcoRI)	mGFP tagged with nur 42 (16K) mutant
pBAD-RsmI-mGFP	*E. coli*	microscopic imaging	pBAD	Restriction ligation (NdeI/EcoRI)	RsmI tagged with mGFP
pBAD-mGFP-RsmI	*E. coli*	microscopic imaging	pBAD	Restriction ligation (NdeI/EcoRI)	mGFP tagged with RsmI
pBAD-TurboID	*E. coli*	protein proximity labelling by TurboID	pBAD	Restriction ligation (NdeI/EcoRI)	TurboID
pBAD-TurboID-C42^{WT}	*E. coli*	protein proximity labelling by TurboID	pBAD	Restriction ligation (NdeI/EcoRI)	TurboID tagged with nur 42 wt
pBAD-TurboID-C42^{16A}	*E. coli*	protein proximity labelling by TurboID	pBAD	Restriction ligation (NdeI/EcoRI)	TurboID tagged with nur 42 (16A) mutant
pAc5.1-Nur^{SP}-ALFA	S2 cell	Western blotting	pAC5.1	Restriction ligation (EcoRI/NotI)	nur wt
pAc5.1-Nur^{S}-ALFA	S2 cell	Western blotting	pAC5.1	Restriction ligation (EcoRI/NotI)	no-sp-nur
pAc5.1-Nur^{AARR}-ALFA	S2 cell	Western blotting	pAC5.1	Restriction ligation (EcoRI/NotI)	nur AARR mutant
pAc5.1-Nur^{AA}-ALFA	S2 cell	Western blotting	pAC5.1	Restriction ligation (EcoRI/NotI)	nur AA mutant
pAc5.1-Nur$^{AARR\text{-}AA}$-ALFA	S2 cell	Western blotting	pAC5.1	Restriction ligation (EcoRI/NotI)	nur AARR-AA mutant
pUAST-attB-Nur-ALFA	*D. melanogaster*	actin-GS or nsyb-Gal4/UAS-NurWT-ALFA	pUAST-attB	Restriction ligation (EcoRI/XbaI)	nur wt
pUAST-attB-mGFP-ALFA	*D. melanogaster*	nsyb-Gal4/UAS-mGFP-ALFA	pUAST-attB	Restriction ligation (EcoRI/XbaI)	mGFP
pUAST-attB-NurAAAA-AA-ALFA	*D. melanogaster*	nsyb-Gal4/UAS-NurAAAA-AA-ALFA	pUAST-attB	Restriction ligation (EcoRI/NoTI)	nur AAAA-AA mutant

Supplementary Table 4. Proteins and peptides used in this study.

Name	Amino acid sequence	MW (g/mol)	Generation method
Nur^{S}-YC	QRSRVLSSRRGSELVEKTSDNKEDSELAAQEQDLERQEQEEQNDRLEGRSDDVAEGSDNKEDKETATNNKDTIVK PNKDDARARRIVRAGRRRGGRRGGRRGGRRSARKSVRRGGRRGGRRRGGRRGRGGARRRTSVKRRSGKGNKAYC	16871.48	recombinantly expressed in *E. coli* and purified in this study
$C62^{WT}$	IVRAGRRRGGRRGGRRGGRRSARKSVRRGGRRGGRRRGGRRGRGGARRRTSVKRRSGKGNKA	6876.91	synthesized in this study
$C20^{WT}$	IVRAGRRRGGRRGGRRGGRR	2262.62	synthesized in this study
Nur #1 fragment	RSRVLSSRRGSELVEKTSDNKEDSELAAQY	3411.9	synthesized by GL Biochem (Shanghai) Ltd.
Nur #3 fragment	KEDKETATNNKDTIVKPNKDDARARRIVRY	3546.6	synthesized by GL Biochem (Shanghai) Ltd.
Nur #4 fragment	AGRRRGGRRGGRRGGRRSARKSVRRGGRRY	3425.6	synthesized by GL Biochem (Shanghai) Ltd.
Nur #5 fragment	GGRRRGGRRGRGGARRRTSVKRRSGKGNKY	3357.2	synthesized by GL Biochem (Shanghai) Ltd.
$C42^{WT}$-Y	SARKSVRRGGRRGGRRRGGRRGRGGARRRTSVKRRSGKGNKAY	4795.48	synthesized by GL Biochem (Shanghai) Ltd.
$C42^{WT}$	SARKSVRRGGRRGGRRRGGRRGRGGARRRTSVKRRSGKGNKA	4632.25	synthesized by GL Biochem (Shanghai) Ltd.
$C42^{4A}$	SARKSVRAGGRAGGRRAGGRRGAGGARRRTSVKRRSGKGNKA	4291.82	synthesized by GL Biochem (Shanghai) Ltd.
$C42^{16A}$	SAAKSVAAGGAAGGAAAGGAAGAGGAAAATSVKAASGKGNKA	3270.52	synthesized by GL Biochem (Shanghai) Ltd.
$C42^{16K}$	SAKKSVKKGGKKGGKKKGGKKGKGGAKKKTSVKKKSGKGNKA	4184.04	synthesized by GL Biochem (Shanghai) Ltd.
$C42^{WT}$-FITC	SARKSVRRGGRRGGRRRGGRRGRGGARRRTSVKRRSGKGNKAK(FITC)	5149.8	synthesized by GL Biochem (Shanghai) Ltd.
$C42^{4A}$-FITC	SARKSVRAGGRAGGRRAGGRRGAGGARRRTSVKRRSGKGNKAK(FITC)	4809.37	synthesized by GL Biochem (Shanghai) Ltd.
$C42^{16A}$-FITC	SAAKSVAAGGAAGGAAAGGAAGAGGAAAATSVKAASGKGNKAK(FITC)	3788.07	synthesized by GL Biochem (Shanghai) Ltd.
$C42^{16K}$-FITC	FITC-Ahx-SAKKSVKKGGKKGGKKKGGKKGKGGAKKKTSVKKKSGKGNKA	4686.58	synthesized by GL Biochem (Shanghai) Ltd.
$C42^{WT}$-AF488	C(AF488)SARKSVRRGGRRGGRRRGGRRGRGGARRRTSVKRRSGKGNKA	5434.14	synthesized by Bio-Synthesis, Inc.
$C42^{WT}$-TAMRA	5-TAMRA-SARKSVRRGGRRGGRRRGGRRGRGGARRRTSVKRRSGKGNKA	5044.69	synthesized by GL Biochem (Shanghai) Ltd.
Magainin 2 F12W	GIGKFLHSAKKWGKAFVGEIMNS	2505.96	synthesized by GL Biochem (Shanghai) Ltd.

Note: Amino acid residues, linkers, and fluorophores shown in red are not native to Nur.

Uncropped gel for Supplementary Fig. 1a

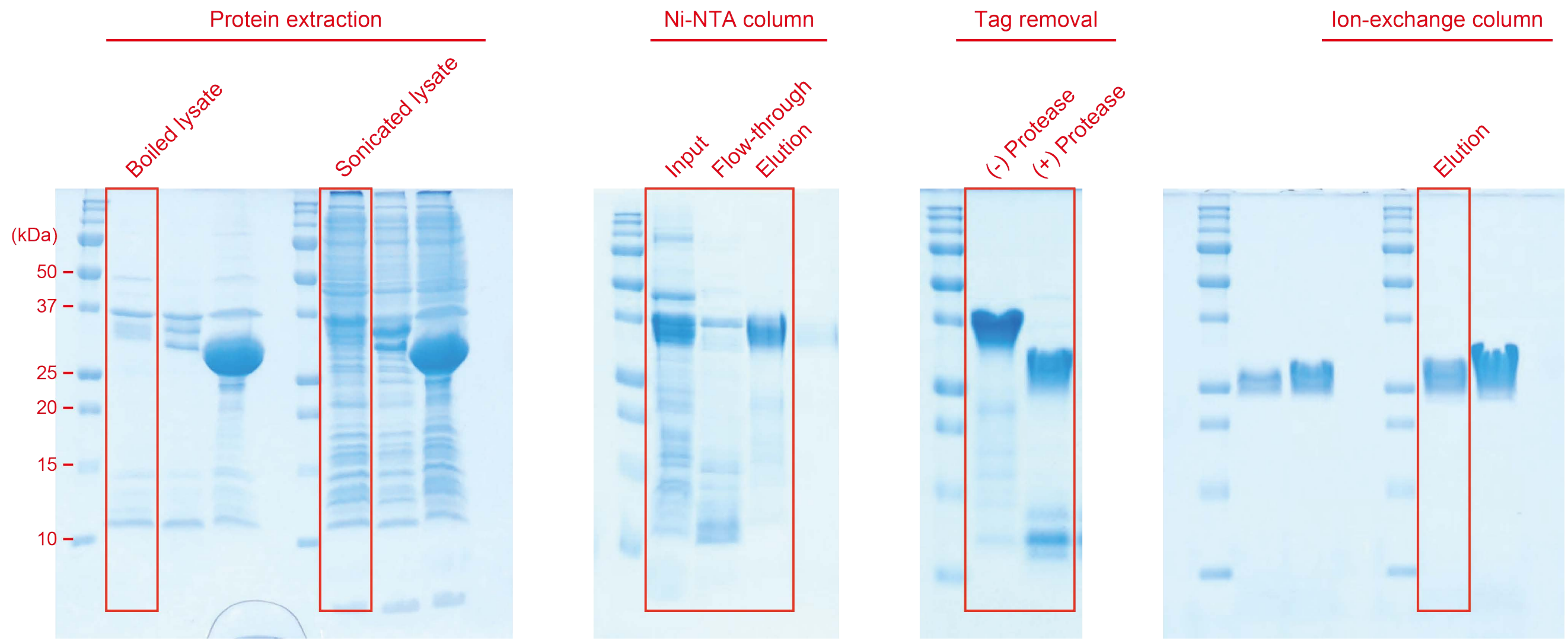

Uncropped blot for Supplementary Fig. 1b, c

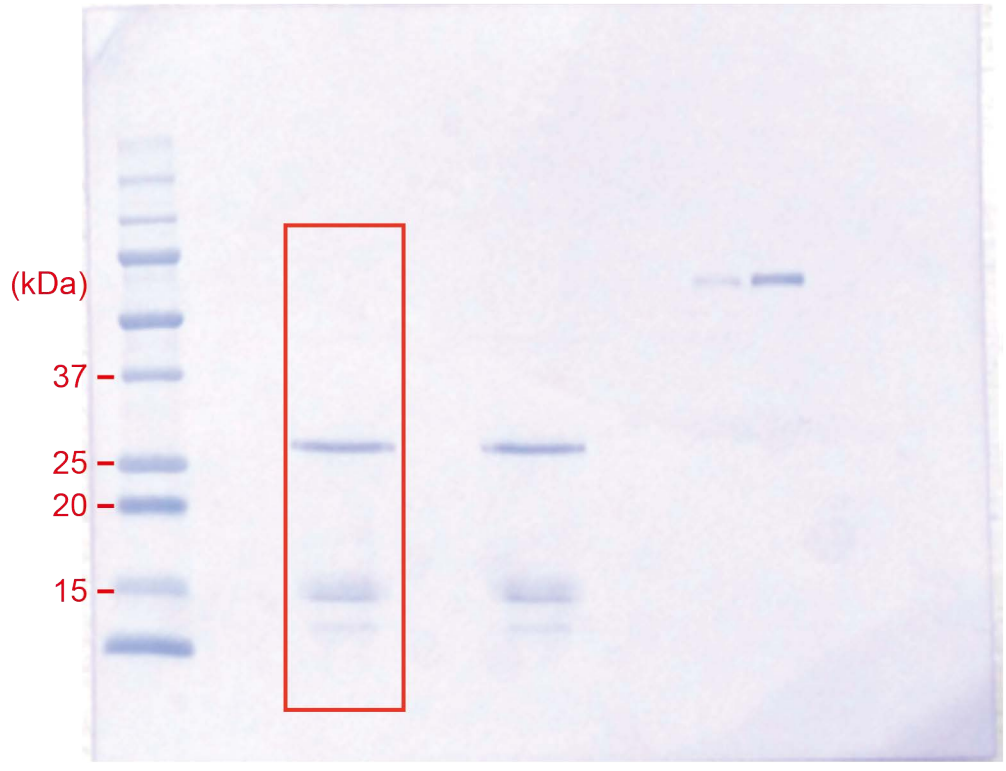

Uncropped blot for Supplementary Fig. 1c

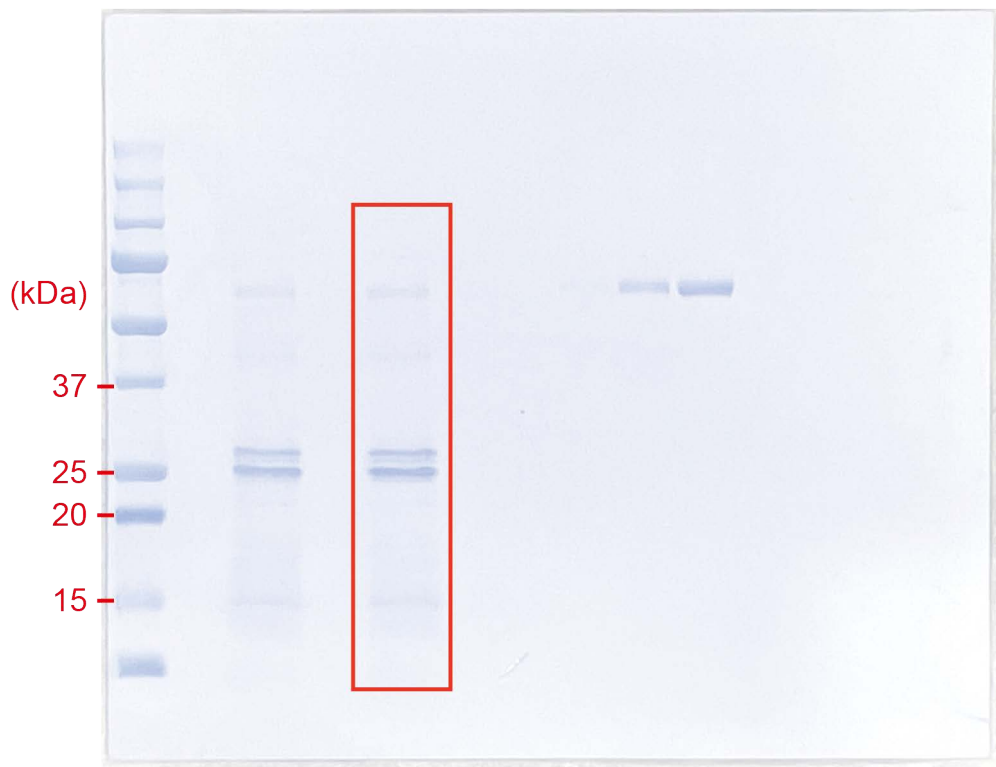

Uncropped blot for Supplementary Fig. 1d

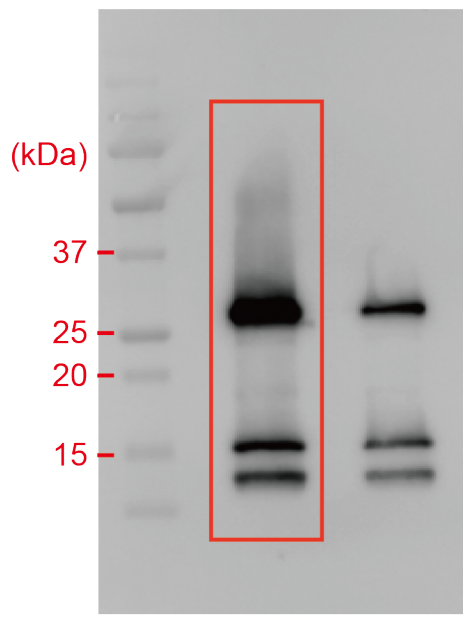

Uncropped blot for Supplementary Fig. 1e

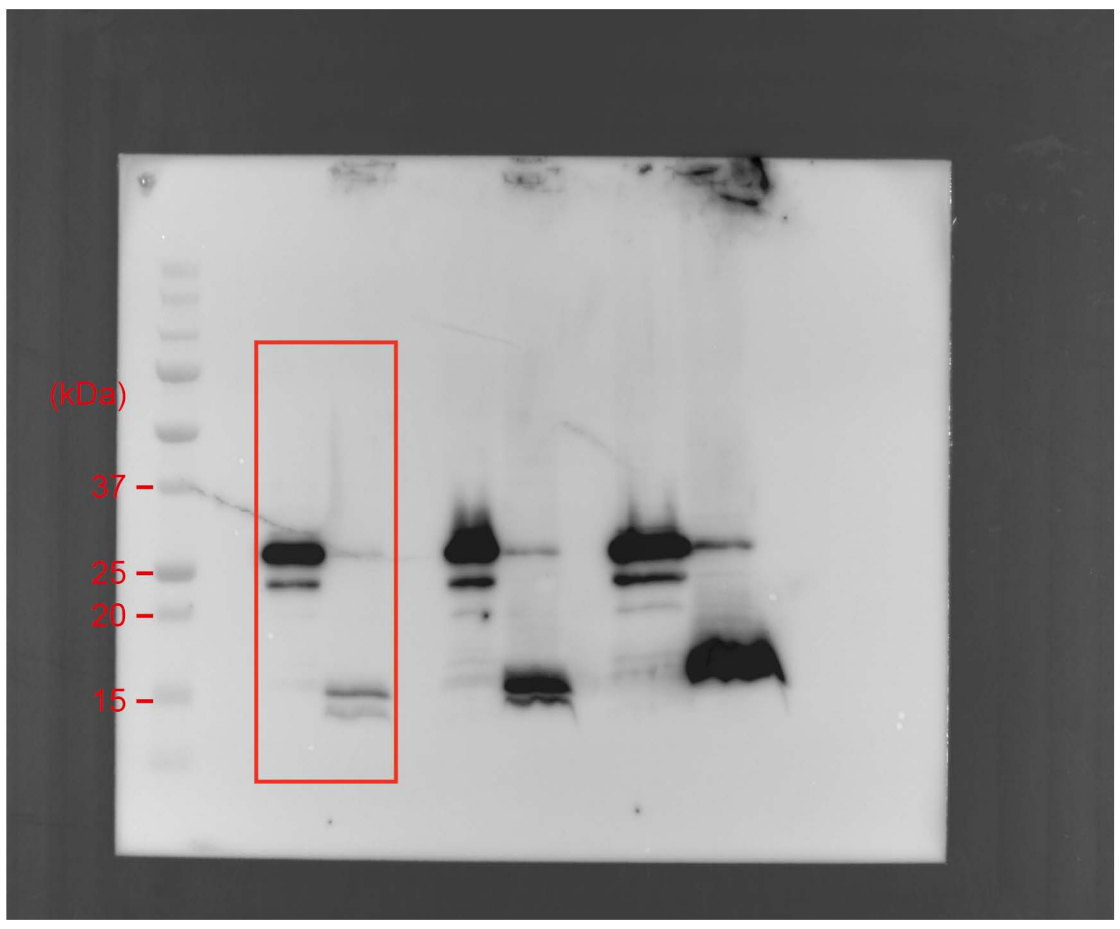

Uncropped blot for Supplementary Fig. 1f

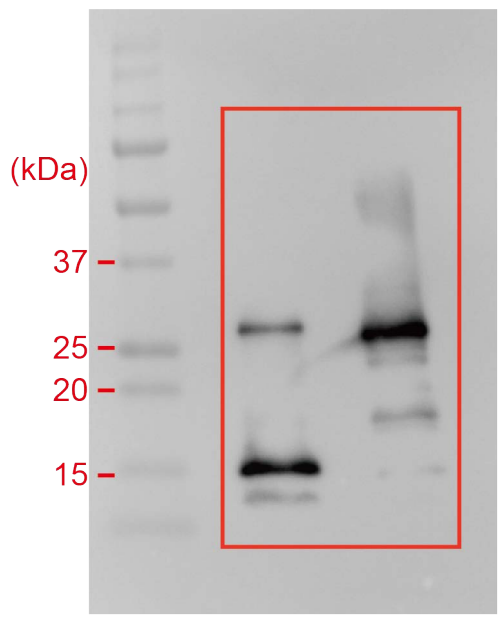

Uncropped agar plate image for Supplementary Fig. 4a

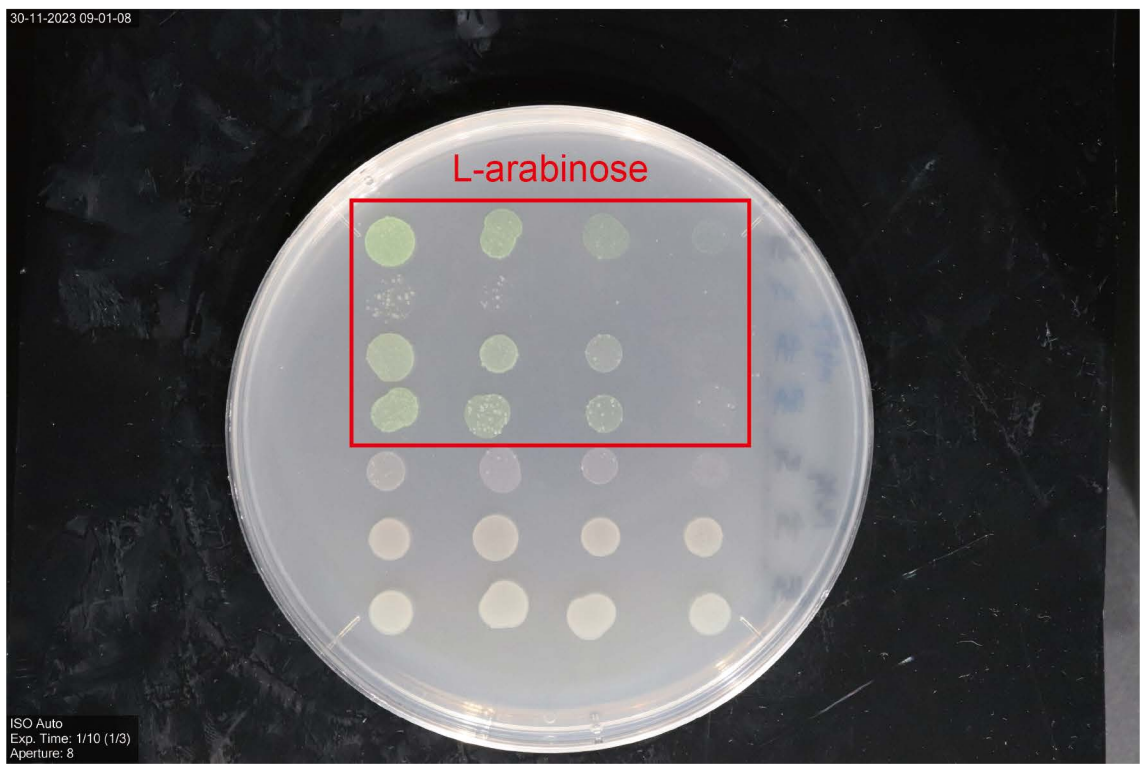

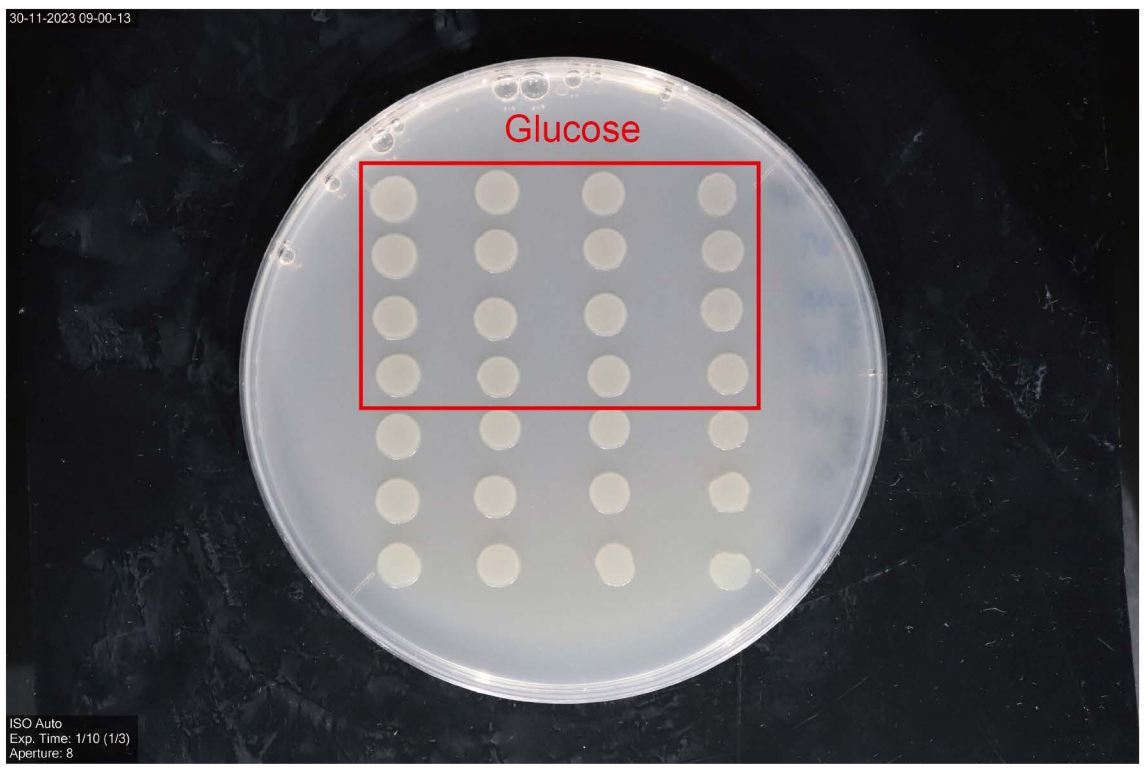

Uncropped agar plate image for Supplementary Fig. 4b

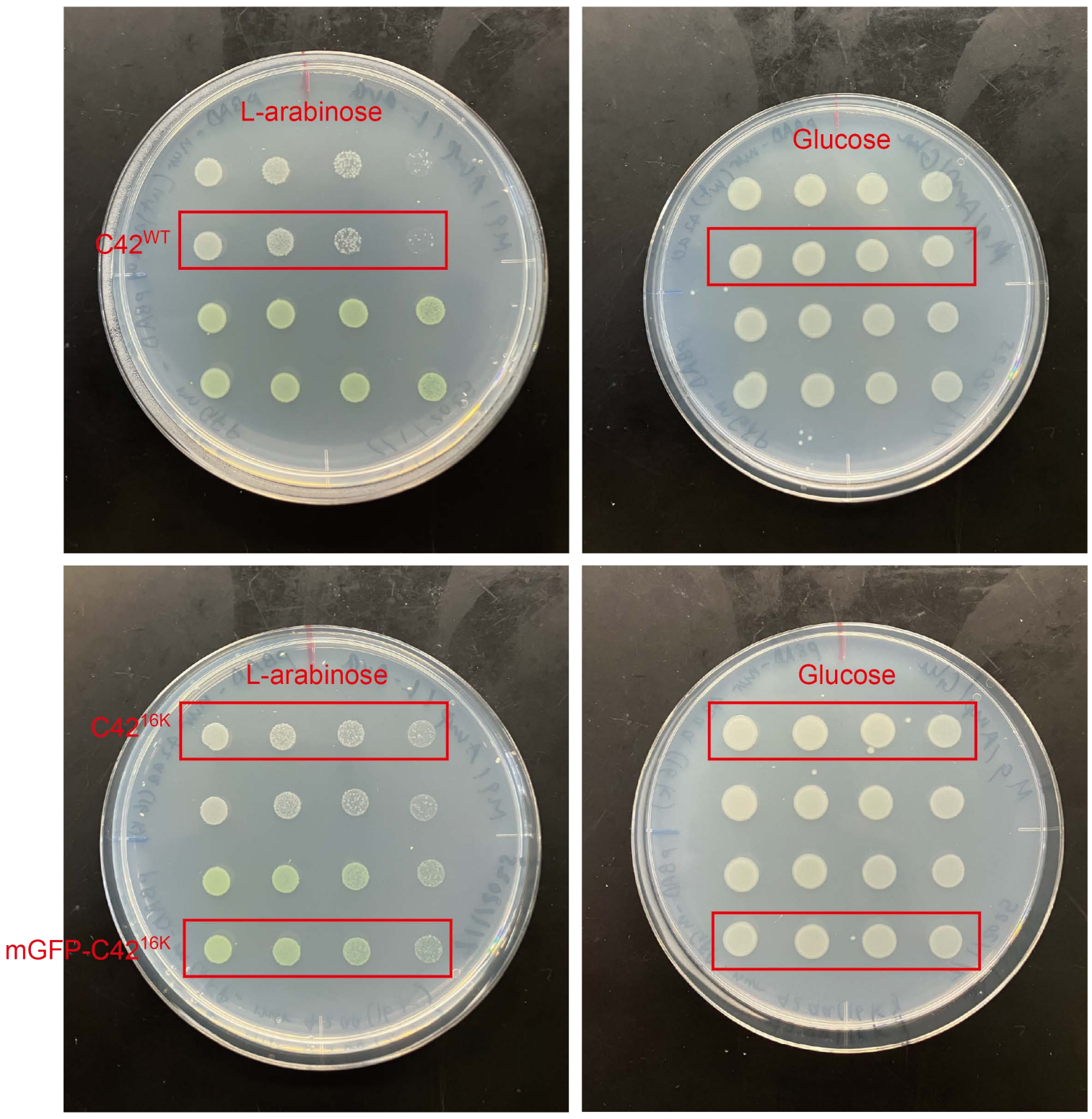

Uncropped agar plate image for Supplementary Fig. 4c

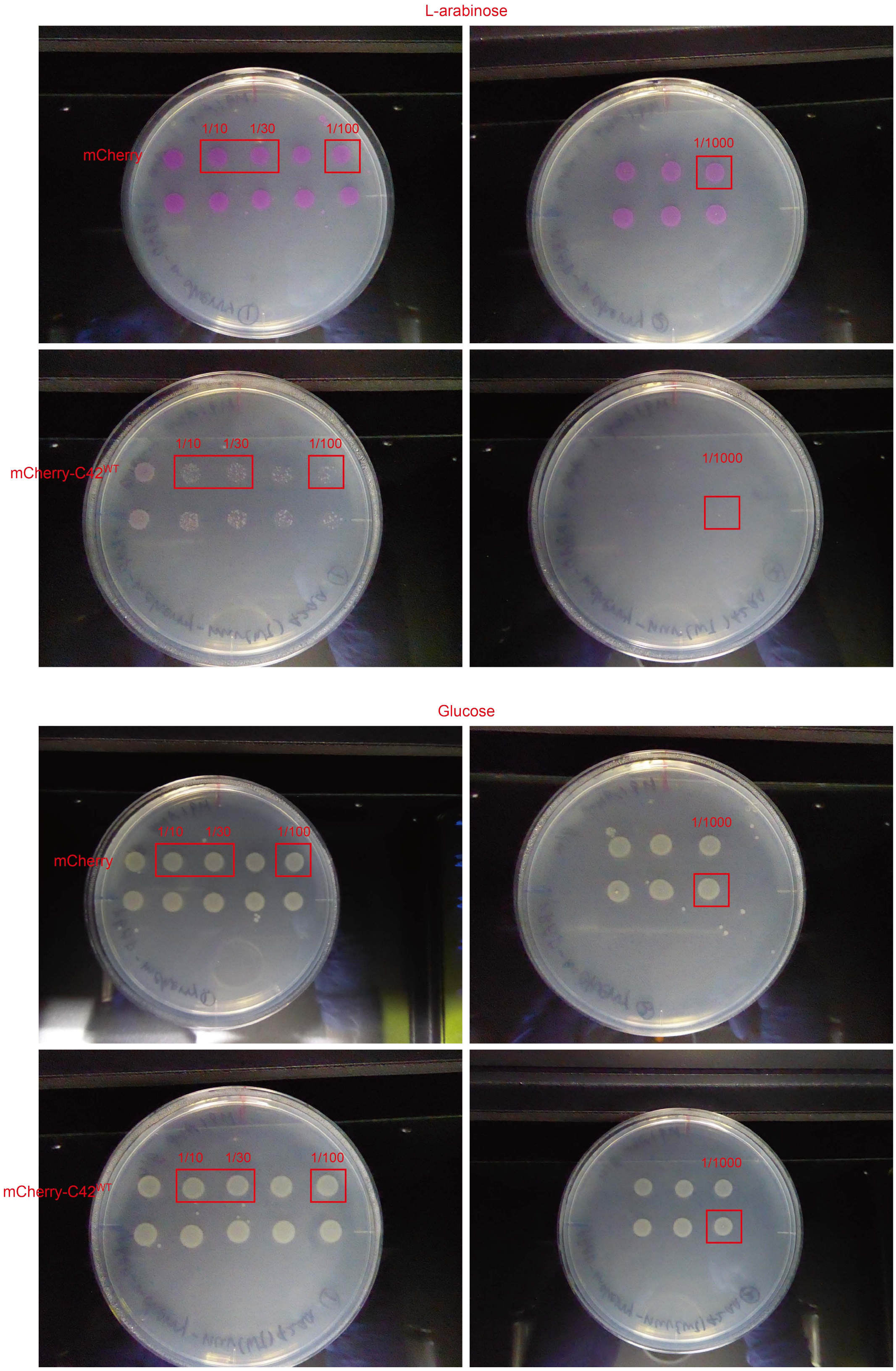

Uncropped agar plate image for Supplementary Fig. 4d

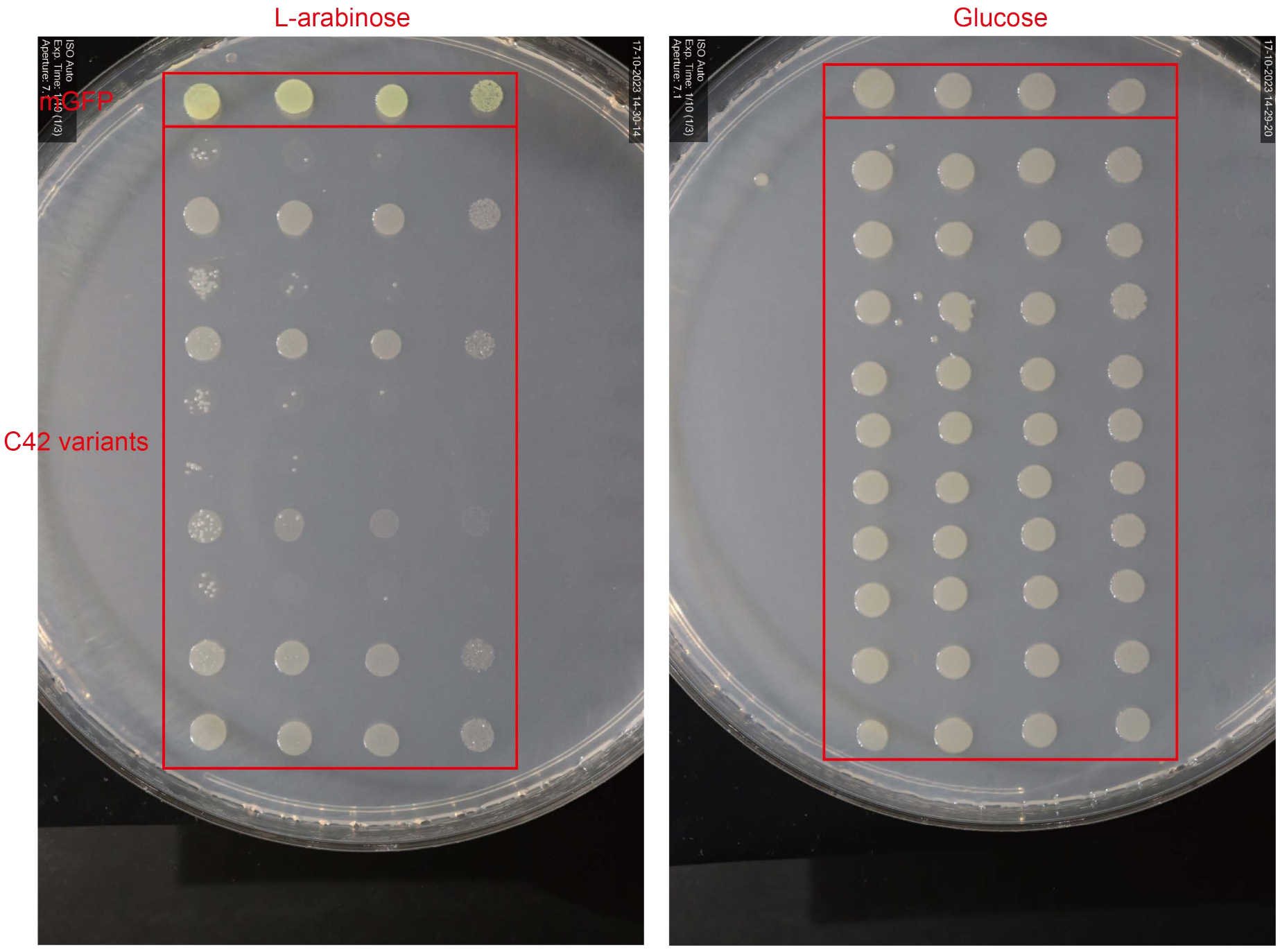

Uncropped blot for Supplementary Fig. 5a

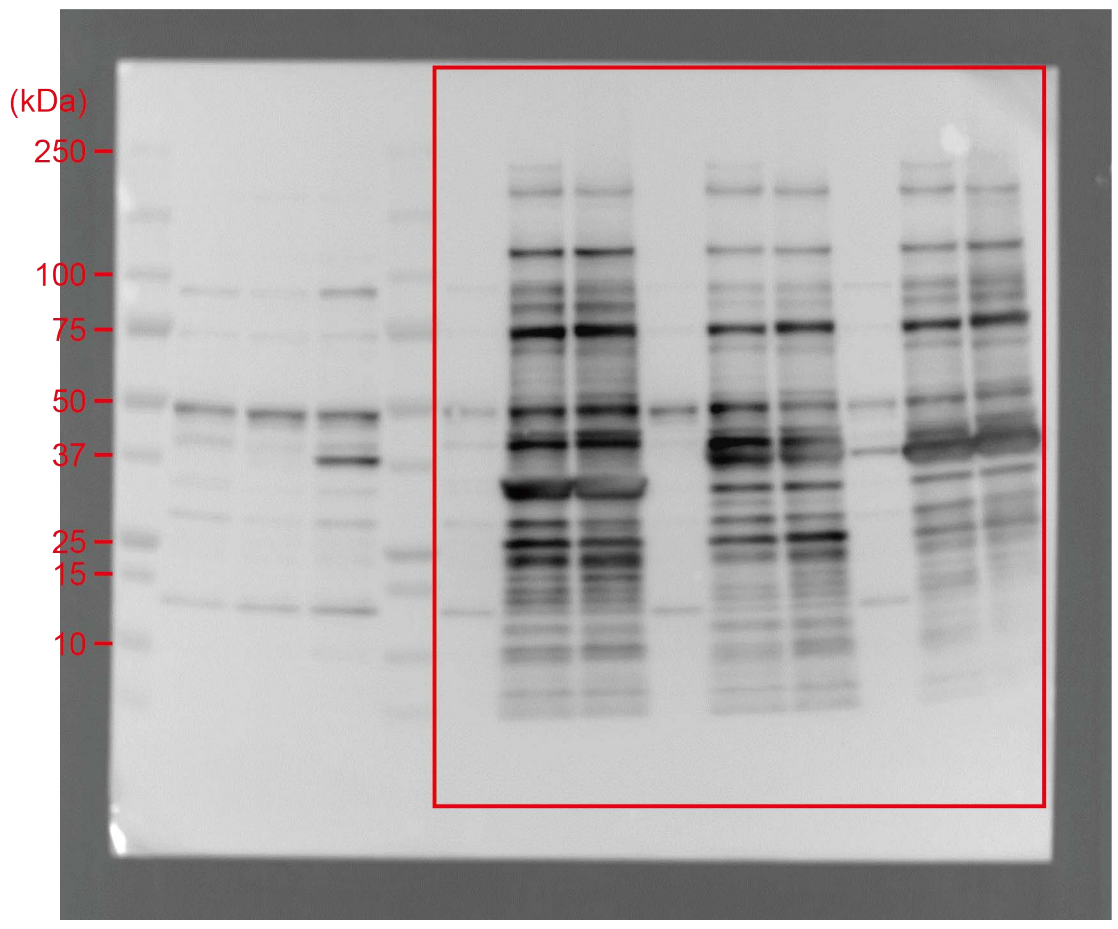

Uncropped blot for Supplementary Fig. 6c

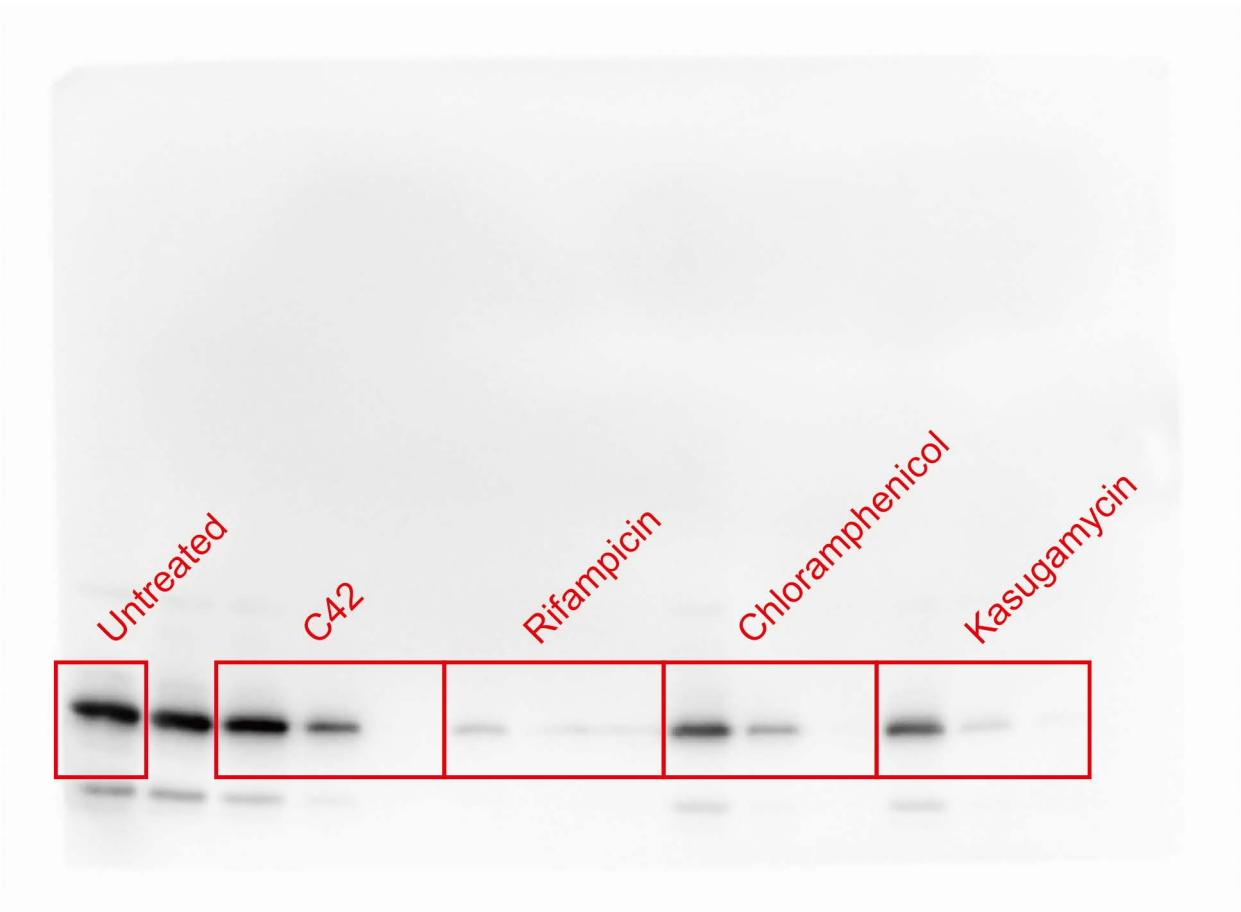

Uncropped gel for Supplementary Fig. 6d

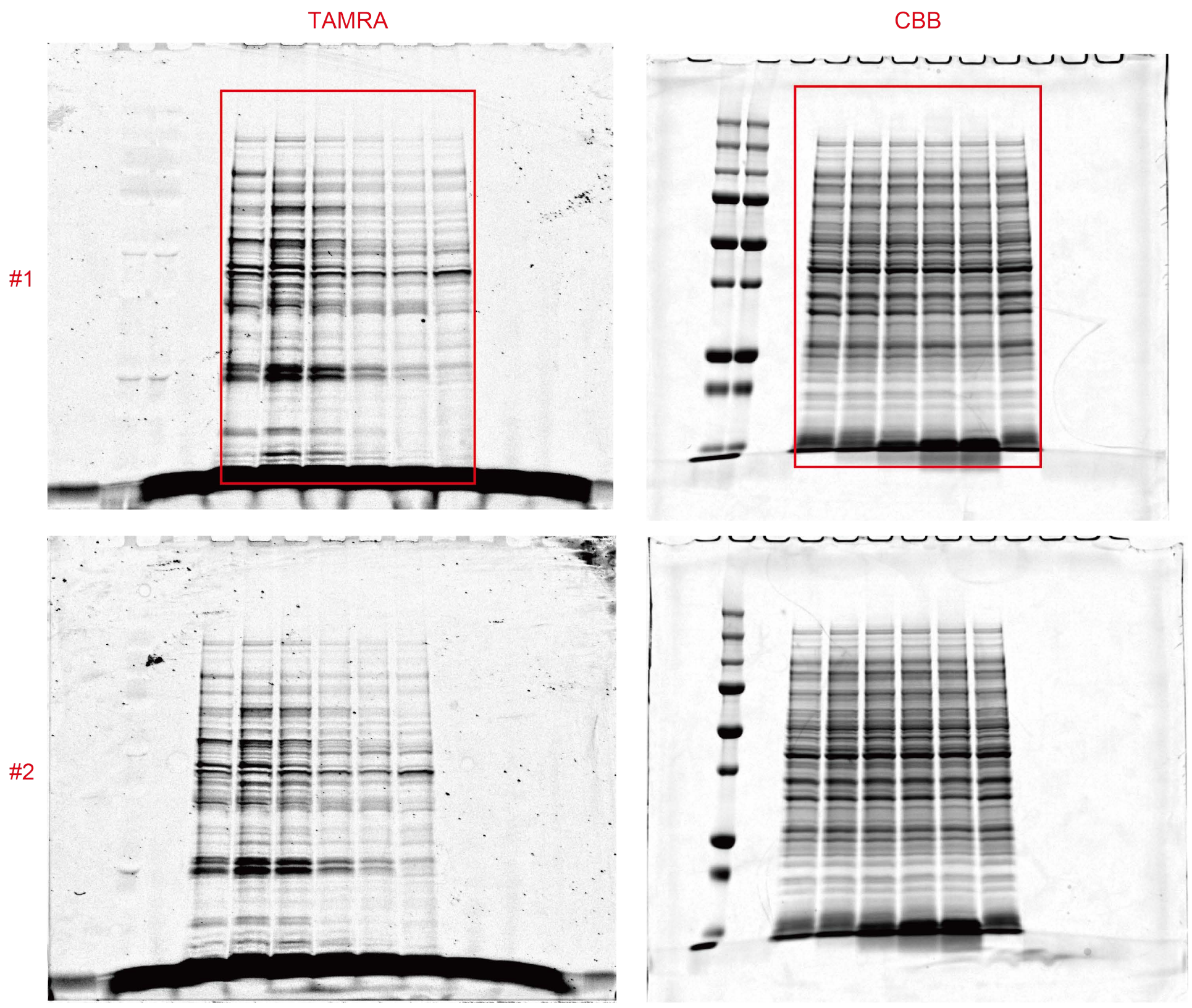

Uncropped gel and blot for Supplementary Fig. 6e

Anti-puromycin

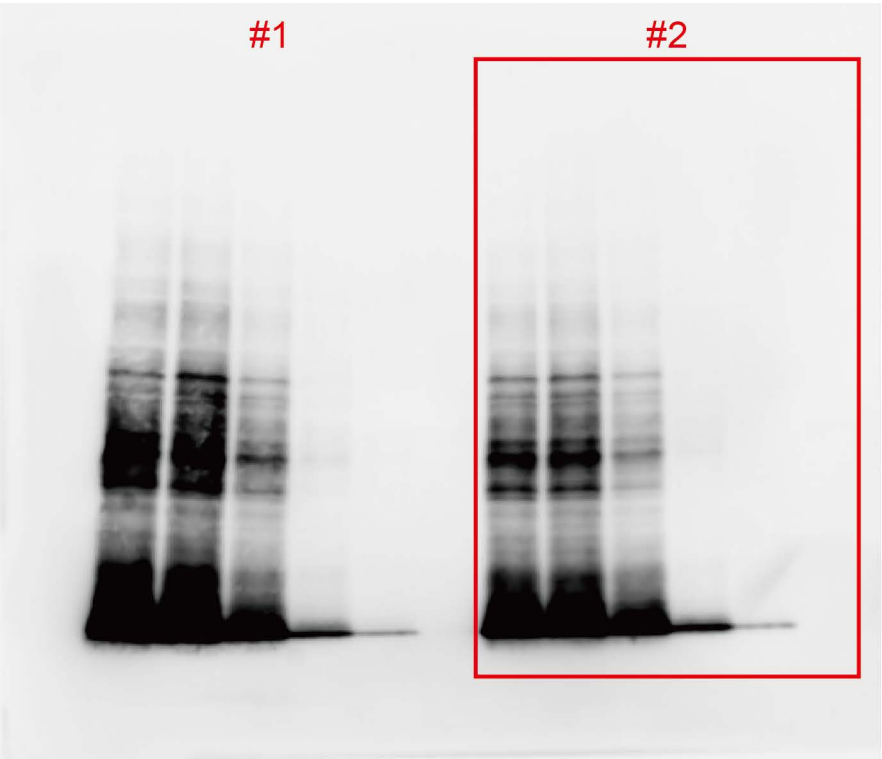

CBB

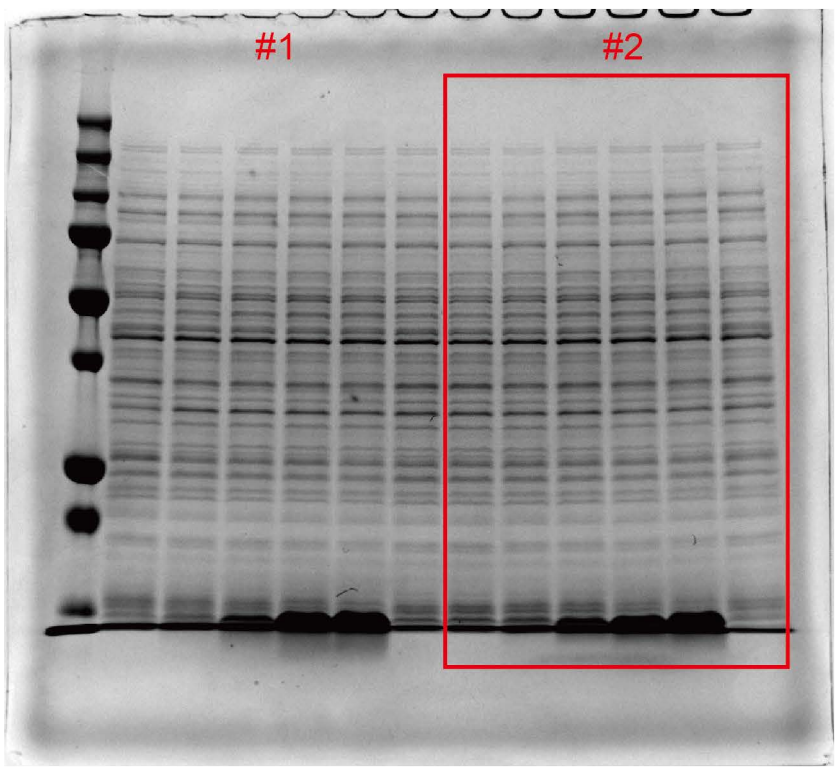

Supplement: Supplementary file 1 [file 41467_2026_78960_MOESM1_ESM.pdf]
