# Supplementary material for: Antimicrobial peptides derived from Nemuri induce ribosomal condensation to inhibit translation
Source: Nat Commun. 2026 Oct 29;17:11037. doi: 10.1038/s41467-026-78960-5 (PMC13720017; doi:10.1038/s41467-026-78960-5)
Supplement: Supplementary file 2 [file 41467_2026_78960_MOESM2_ESM.pdf]

Description of Additional Supplementary Files

File Name: Supplementary Data 1
Description: Processed TurboID proteomics data. This Excel file contains the list of all identified proteins, the analysis used to identify 58 proteins enriched in TurboID-C42 samples, and their abundance profiles and Gene Ontology annotations.

File Name: Supplementary Data 2
Description: Processed XL-MS data. This Excel file contains C42–ribosomal protein cross-links identified by XL MS at the indicated C42 concentrations, including maximum XlinkX scores and numbers of cross-link spectrum matches.
