# Supplementary material for: Antimicrobial peptides derived from Nemuri induce ribosomal condensation to inhibit translation
Source: Nat Commun. 2026 Oct 29;17:11037. doi: 10.1038/s41467-026-78960-5 (PMC13720017; doi:10.1038/s41467-026-78960-5)
Supplement: Supplementary file 3 [file 41467_2026_78960_MOESM5_ESM.pdf]

Corresponding author(s): Nobuo N. Noda, Hirofumi Toda

Last updated by author(s): Oct 7, 2026

Reporting Summary

Nature Portfolio wishes to improve the reproducibility of the work that we publish. This form provides structure for consistency and transparency in reporting. For further information on Nature Portfolio policies, see our Editorial Policies and the Editorial Policy Checklist.

Statistics

For all statistical analyses, confirm that the following items are present in the figure legend, table legend, main text, or Methods section.

n/a	Confirmed	
☐	☒	The exact sample size (*n*) for each experimental group/condition, given as a discrete number and unit of measurement
☐	☒	A statement on whether measurements were taken from distinct samples or whether the same sample was measured repeatedly
☐	☒	The statistical test(s) used AND whether they are one- or two-sided<br>*Only common tests should be described solely by name; describe more complex techniques in the Methods section.*
☒	☐	A description of all covariates tested
☐	☒	A description of any assumptions or corrections, such as tests of normality and adjustment for multiple comparisons
☐	☒	A full description of the statistical parameters including central tendency (e.g. means) or other basic estimates (e.g. regression coefficient) AND variation (e.g. standard deviation) or associated estimates of uncertainty (e.g. confidence intervals)
☐	☒	For null hypothesis testing, the test statistic (e.g. *F*, *t*, *r*) with confidence intervals, effect sizes, degrees of freedom and *P* value noted<br>*Give P values as exact values whenever suitable.*
☒	☐	For Bayesian analysis, information on the choice of priors and Markov chain Monte Carlo settings
☒	☐	For hierarchical and complex designs, identification of the appropriate level for tests and full reporting of outcomes
☒	☐	Estimates of effect sizes (e.g. Cohen's *d*, Pearson's *r*), indicating how they were calculated

Our web collection on statistics for biologists contains articles on many of the points above.

Software and code

Policy information about availability of computer code

Data collection	Confocal images were obtained using FV3000 (Olympus), A1 with ECLIPSE Ti2 (Nikon), LSM800, and LSM900 with Airyscan 2 (Zeiss).<br>Raman images were obtained using Nanofinder flex2 (Tokyo Instruments) integrated with ECLIPSE Ti2 (Nikon).<br>3D refractive index images were obtained using HT-2H (Tomocube).<br>Spinning disk confocal images were obtained using X-light V2 (Crest Optics).<br>Electron microscopy data were obtained using JEM-1400plus (JEOL) and Glacios cryo-TEM equipped with a Falcon4i direct electron detector (Thermo Fisher Scientific). Cryo-EM grids were vitrified using a Vitrobot Mark IV (Thermo Fisher Scientific).<br>Spectroscopic and plate reader data were obtained using J-820 (JASCO), Synergy LX and PowerScan HT (BioTek), Infinite 200 Pro (Tecan), Varioskan LUX (Thermo Fisher Scientific), NanoPhotometer NP80 Touch (IMPLEN), and Agilent 2100 Bioanalyzer (Agilent).<br>NMR data were obtained using AVANCE NEO 800 MHz spectrometer (Bruker).<br>Mass spectrometry data were obtained using Q Exactive and Q Exactive Plus (Thermo Fisher Scientific).<br>Gel and blot images were obtained using Fusion Solo S (Vilber Bio Imaging), FAS-DiGiPRO (Nippon Genetics), Amersham Typhoon scanner (Cytiva), and LAS4000 image analyzer (FUJIFILM).<br>qPCR data were obtained using CFX96 Real-Time PCR system (Bio-Rad).
Data analysis	Image and gel/blot intensity analysis: Fiji/ImageJ and Multi Gauge 3.1 (FUJIFILM)<br>Raman and fluorescence data analysis: IGOR Pro (WaveMetrics)<br>Electron microscopy data analysis: RELION-5<br>NMR data analysis: TopSpin 4.4.1 (Bruker) and Sparky 3.113<br>Mass spectrometry data analysis: Proteome Discoverer 3.1, Xcalibur 4.0, and XlinkX (Thermo Fisher Scientific)<br>Molecular visualization: UCSF ChimeraX

CD data analysis: BeStSel
Protein disorder predictions: PrDOS and AIUPred
Gene Ontology enrichment analysis: DAVID
Statistical analysis: GraphPad Prism 10.2.2 (GraphPad Software)

For manuscripts utilizing custom algorithms or software that are central to the research but not yet described in published literature, software must be made available to editors and reviewers. We strongly encourage code deposition in a community repository (e.g. GitHub). See the Nature Portfolio guidelines for submitting code & software for further information.

Data

Policy information about availability of data

All manuscripts must include a data availability statement. This statement should provide the following information, where applicable:

- Accession codes, unique identifiers, or web links for publicly available datasets
- A description of any restrictions on data availability
- For clinical datasets or third party data, please ensure that the statement adheres to our policy

The mass spectrometry-based proteomics data generated in the TurboID experiments have been deposited in the ProteomeXchange Consortium via the PRIDE partner repository under accession code PXD072620 (https://www.ebi.ac.uk/pride/archive/projects/PXD072620). The processed TurboID proteomics data are provided in Supplementary Data 1. The XL-MS data have been deposited in the ProteomeXchange Consortium via the jPOST partner repository under accession codes PXD073821 and JPST004364 (https://repository.jpostdb.org/entry/JPST004364). The processed XL-MS data are provided in Supplementary Data 2. The negative-stain EM map has been deposited in the Electron Microscopy Data Bank (EMDB) under accession code EMD-68592 (https://www.ebi.ac.uk/emdb/EMD-68592). The microscopy image data have been deposited in figshare (https://doi.org/10.6084/m9.figshare.33364299). All other data supporting the findings of this study are available within the article and its supplementary information files. Source data are provided with this paper.

Research involving human participants, their data, or biological material

Policy information about studies with human participants or human data. See also policy information about sex, gender (identity/presentation), and sexual orientation and race, ethnicity and racism.

Reporting on sex and gender	n/a
Reporting on race, ethnicity, or other socially relevant groupings	n/a
Population characteristics	n/a
Recruitment	n/a
Ethics oversight	n/a

Note that full information on the approval of the study protocol must also be provided in the manuscript.

Field-specific reporting

Please select the one below that is the best fit for your research. If you are not sure, read the appropriate sections before making your selection.

☒ Life sciences ☐ Behavioural & social sciences ☐ Ecological, evolutionary & environmental sciences

For a reference copy of the document with all sections, see nature.com/documents/nr-reporting-summary-flat.pdf

Life sciences study design

All studies must disclose on these points even when the disclosure is negative.

Sample size	Sample sizes for bacterial, eukaryotic cell, and fly experiments were determined based on standard practices and sample sizes commonly used in this field.
Data exclusions	No exclusion.
Replication	All experiments were performed independently two or more times with similar results.
Randomization	Randomization is not relevant to this study because the used samples are restricted to genetically defined organisms, cultured cells, purified protein, and peptides.
Blinding	Blinding is not relevant to this study because the experimental groups were defined by genotype or specific treatments.

Reporting for specific materials, systems and methods

We require information from authors about some types of materials, experimental systems and methods used in many studies. Here, indicate whether each material, system or method listed is relevant to your study. If you are not sure if a list item applies to your research, read the appropriate section before selecting a response.

Materials & experimental systems

n/a	Involved in the study
☐	☒ Antibodies
☐	☒ Eukaryotic cell lines
☒	☐ Palaeontology and archaeology
☐	☒ Animals and other organisms
☒	☐ Clinical data
☒	☐ Dual use research of concern
☒	☐ Plants

Methods

n/a	Involved in the study
☒	☐ ChIP-seq
☒	☐ Flow cytometry
☒	☐ MRI-based neuroimaging

Antibodies

Antibodies used	HRP-conjugated anti-ALFA antibody (NanoTag Biotechnologies, cat. no. N1505-HRP, clone 1G5, lot no. 072407, 1:4,000)<br>Mouse monoclonal anti-GFP antibody (Roche, cat. no. 11814460001, clones 7.1 and 13.1, lot no. 70378300, 1:1,000)<br>Peroxidase-conjugated AffiniPure Donkey anti-Mouse IgG (Jackson ImmunoResearch, cat. no. 715-035-151, lot no. 168069, 1:5,000)<br>Mouse monoclonal anti-puromycin antibody (Sigma-Aldrich, cat. no. MABE343, clone 12D10, 1:20,000)<br>HRP-conjugated goat anti-mouse IgG (Sigma-Aldrich, cat. no. A4416, 1:10,000).
Validation	Validation for the HRP-conjugated anti-ALFA antibody was described in https://nano-tag.com/product/anti-alfa-sdab/. Validation for the anti-GFP antibody was described in https://www.sigmaaldrich.com/US/en/product/roche/11814460001. Validation for the Peroxidase-conjugated AffiniPure Donkey anti-Mouse IgG was described in https://www.jacksonimmuno.com/catalog/products/715-035-151. Validation for the anti-puromycin antibody was described in https://www.sigmaaldrich.com/NL/en/product/mm/mabe343. Validation for the HRP-conjugated goat anti-mouse IgG was described in https://www.sigmaaldrich.com/HR/en/product/sigma/a4416.

Eukaryotic cell lines

Policy information about cell lines and Sex and Gender in Research

Cell line source(s)	Drosophila S2 and S2R+ cells were provided by R. Niwa and M. Miura. Human U2OS cells were provided by A. Suzuki. Human PANC-1 cells and murine L-1210 cells were obtained from the Institute of Microbial Chemistry (BIKAKEN).
Authentication	The cell lines were not authenticated.
Mycoplasma contamination	All cell lines tested negative for mycoplasma contamination.
Commonly misidentified lines (See ICLAC register)	No commonly misidentified cell lines were used.

Animals and other research organisms

Policy information about studies involving animals; ARRIVE guidelines recommended for reporting animal research, and Sex and Gender in Research

Laboratory animals	Drosophila melanogaster
Wild animals	n/a
Reporting on sex	mixed
Field-collected samples	n/a
Ethics oversight	Not required under institutional guidelines for Drosophila

Note that full information on the approval of the study protocol must also be provided in the manuscript.

Plants

Seed stocks	n/a
Novel plant genotypes	n/a
Authentication	n/a
