# Supplementary material for: Antimicrobial peptides derived from Nemuri induce ribosomal condensation to inhibit translation
Source: Nat Commun. 2026 Oct 29;17:11037. doi: 10.1038/s41467-026-78960-5 (PMC13720017; doi:10.1038/s41467-026-78960-5)
Supplement: Supplementary file 4 [file 41467_2026_78960_MOESM6_ESM.pdf]

nature portfolio

Peer Review File

Antimicrobial peptides derived from Nemuri induce ribosomal condensation to inhibit translation

Corresponding Author: Professor Hirofumi Toda

This file contains all reviewer reports in order by version, followed by all author rebuttals in order by version.

Version 0:

Reviewer comments:

Reviewer #1

(Remarks to the Author)
The growing increase in multidrug resistant bacteria represents a global threat that highlights the urgent need for new antimicrobial agents. One alternative to small molecule antibiotics is the development of antimicrobial peptides (AMPs) for therapeutic use. While most AMPs kill bacteria by disrupting the cell membrane, specific subclass of AMPs have been identified that pass through the membrane and target intracellular processes.
The manuscript of Nishinami and coworkers follows previous work identfying a Drosophila peptide termed Nemuri (Nur) that can kill bacteria, however, the mechanism has remained unclear. In this manuscript, the authors demonstrate that Nur undergoes proteolytic cleavage upon expression and that the C-terminal region (C62) is responsible for the killing activity and contains two subpeptides C20 and C42 that display similar MIC. The peptides are shown to have broadspectrum activity against a range of Gram-positive and Gram-negative bacteria, with C62 being the most active, followed by C42 and C20. The peptides did not display toxicity against Drosophila S2R+, murine L-1210 and human PANC-1 cells. The authors then focus on the C42 peptide (why not the most active C62 peptide?) and show that it associates with ribosomes and inhibits translation. Rather then a specific inhibition, the authors suggest that the intrinsically disordered regions of the peptide target ribosomal liquid condensates and reduce their fluidity.
Overall, the manuscript is well written, results nicely presented and appropriately interpreted. The finding of novel peptides causing ribosomal condensation will be of general interest to the translation community. I have only a series of comments, which if addressed, should improve the clarity of the manuscript.

Points to address.
1. The Nur peptides are bactericidal, yet the experiments only report MIC and no MBC values are shown. Is the bactericidal effect only observed at higher concentrations where membrane damage is observed or is there another mechanism for their bactericidal activity?
2. The Nur peptides inhibit translation in vivo and in vitro, yet I found it difficult to understand if the inhibition of translation is occurring at the same concentrations as the condensate formation i.e. is it possible that these are uncoupled such that translation is inhibited at lower concentrations than where LLPS is observed?
3. In Figure 2k, it would be better to have concentration as the x-axis…Time is not very informative. If more concentrations were tested, then one can determine IC50 easier. Also I don’t see replicates anywhere or statistics for signifcance. The 4A peptide has still quite some activity at inhibiting translation – does this correlate with the expected ability to still form condensates?
4. The in vitro assay is coupled. Perhaps it would be better to use transcribed mRNA and determine directly the IC50 of peptides on translation and separately determine the IC50 for transciption.
5. Do condensates still form if one inhibits translation in vivo with other antibiotics, e.g. Ksg or Chl, prior to addition of C42 peptides? Just wondering whether actively translating ribosomes can end up in condensates.
6. Line 275, the crosslinking experiment is not well explained but appears to crosslink lysines of ribosomal proteins to the peptides - This is not exactly mapping the binding site of the peptides on the ribosome…the peptides interact with RNA and not protein and the ribosomes are predominantly rRNA – can one crosslink the peptides to rRNA and more accurately define the scope of the peptide binding sites on the ribosome.
7. In Fig 1f, the C20 peptide is assigned region 111-172, but presumably this should read 111-131.
8. In Figure 4f and ED Fig 9d ribosomes are visualized using EM, however, the results are confusing. In Figure 4f, there appear to be some blobs when ribosomes and C42 are mixed…are these condensates? Do they contain ribosomes or are they just ethane blobs? There don’t appear to be any controls using inactive peptides or positive control…also why does

one no see ribosomes in 4f where C42 is not added? In ED Fig 9d there are lots of ribosomes visualized by negative strain EM but in the absence of C42…where is the experiment with C42 present and other controls? In ED Fig 10b there appear to be lots of aggregates when C42 is mixed with ribosomes and visualized by cryo-EM, but I don't see any controls with ribosomes in the absence of C42. Do the same aggregates form when using 30S or 50S rather than 70S particles?
9. Perhaps some discussion about the feasibility of these peptides as antibiotics could be added in the discussion section. Generally, one would want the smallest peptide fragment that retains activity but the C20 and C42 already see loses of activity in the MICs. Is it feasible to use the full-length peptide.

Reviewer #2

(Remarks to the Author)
Nishinami et al. investigate the antimicrobial mechanism by cleavage fragments of the nemuri peptide. In previous work, the authors made the riveting discovery that this peptide functions in the regulation of sleep in Drosophila but can moonlight as an antimicrobial peptide (AMP) as well. Here, the authors identify cationic cleavage fragments of the full-length protein that are secreted and responsible for the antimicrobial activity. They continue to explore how these peptides exert their function. AMPs come in many flavors. While the initial focus on the field centered on amphiphilic helical peptides that are membrane active (i.e., their chemistry allows them to insert themselves into membranes to form pores), in recent years accumulating evidence from both prokaryote and eukaryote systems suggests that another class of AMPs is pervasive: disordered cationic peptides. These peptides have been shown by several groups to spontaneously enter cells, without overt membrane disruptions, and subsequently kill cells by binding to RNA and ribosomes, owing to their charge, and shutting down translation. The authors find that the nemuri cleavage fragments follow a similar principle: their evidence strongly suggests that these peptides enter cells, slow down the diffusion of ribosomes (indicating their interaction with them), and halt translation. Unfortunately, the authors failed to properly cite the rich literature that preceded them and had already laid out these biophysical principles in great detail. Was this done to enhance the "novelty" of the work? Additionally, the authors make some puzzling conclusions from their experimental observations and bold claims regarding the role for liquid-liquid phase separation as a major organizational mechanism of the bacterial cytosol. In my opinion, these claims are not grounded in fact or supported by the experimental evidence. I will explain why.

First, several papers by now have shown that cationic peptides bind to ribosomes in prokaryote and eukaryote systems. Hereby, they can (1) prevent ribosomal assembly (Kwon et al. 2014), (2) directly impede translation by binding to catalytic sites (Loveland et al. 2022), (3) condense ribosomes via complex coacervation (Sneideris et al. 2023), or (4) cause ribosomal-mediated cytosolic gelation (Zhu, Mohapatra, and Weisshaar 2019). The authors do cite some papers in the introduction but fail to acknowledge the rich detail these papers have already given us into the mechanism of cationic peptides like the ones studied here. Moreover, they especially fail to mention that ribosomal condensation and clustering have been previously reported and studied in great mechanistic and biophysical detail:

(Schavemaker, Śmigiel, and Poolman 2017) - Demonstrated that positively charged proteins bind ribosomes in the bacterial cytosol and slow down their diffusion dynamics.

(Chongsiriwatana et al. 2017) - Demonstrated that cationic AMPs drive ribosome coacervation in bacteria, which correlates with their killing activity.

(Boeynaems et al. 2023) - Demonstrated that cationic peptides can cause the coacervation of many anionic proteins, including ribosomes, in eukaryote and prokaryote systems.

As the main claim of this paper is they discovered a novel mechanism that centers on the condensation of ribosomes. It is puzzling as to why these previous reports were omitted.

Second, the authors make the bold claim that the cationic peptides they study target pre-existing ribosomal condensates, which subsequently harden. It is my understanding that these claims were made on an incorrect understanding of the cellular organization of bacteria. Let me elaborate. The authors image nucleoids and ribosomes. In actively growing bacteria that have ongoing transcription, it is well documented that the nucleoid forms its own entity that excludes ribosomes. There are countless papers that have illuminated key details of this organizational principle. This is a great example: (Gray et al. 2019). So, if the nucleoid is its own entity, with a certain mesh size and rigidity, it will obviously act as a ribosome exclusion barrier. The same is true for protein-based (Bowman et al. 2010) and glycogen-based (Thappeta et al. 2025) bacterial condensates. Thus, the ribosomes occupy all empty space in a bacterial cell that is not occupied by other condensates or large assemblies. We also call this…the cytosol (i.e., the cytosolic space that is not occupied by large stuff). In this study, the authors label the entire cytosolic space of these bacteria first as "ribosome-rich regions". Nothing wrong with saying that, but at the end of the day it remains simply the cytosol. However, the authors proceed to call this region "ribosomal liquid condensates". Why is puzzling. It seems the authors make this conclusion based on three observations that by no means can be considered proof of the existence of a large ribosomal enriched condensates that takes up the whole cell expect the nucleoid. In other words, I do not think any proof has been shown to justify that we from now on consider the cytosol of any cell as a condensate.

(1) Ribosomes recover via FRAP in cells indicating they are liquid part of liquid compartments. Yes, this is true. They are part of the cytosol and will therefore diffuse. I do understand how diffusion of a protein in the cytosol implies that the cytosol

is a liquid. I agree with that statement. I just fail to understand why this means we need to describe the cytosol as a whole as a condensate.
(2) The authors can phase separate DNA and RNA (or ribosomes) in vitro by combining it with poly-L-lysine, an extremely charged polymer that will drive their condensation via complex coacervation. These mixtures form multiphase solid-like gels (indicated by the arrested fusion events visible in the pictures). This is something that has been done by countless labs that have generated multiphase assemblies with countless proteins and nucleic acids in the test tube. How any of this can be interpreted as proof that ribosomes in cells can only exist in a condensed state is a mystery. The fact that the peptides prefer to target the RNA phase of these in vitro assemblies is expected based on the favorable interactions of disordered peptides with ssRNA over DNA (this relates to backbone flexibility).
(3) The authors claim to see condensate hardening. I 100% agree that the data support a slowing and even arrest of ribosomal diffusion, as is expected based on the interaction with and crosslinking of ribosomes by these peptides (Schavemaker, Śmigiel, and Poolman 2017). But again, it is not because the ribosomes stop diffusion once a cell becomes overrun with cationic peptides that this meant that the ribosomes needed to exist in a condensed state prior. What the authors essentially observe is the complete gelation of the cytosol as a whole. This is exactly what other have observed and measured before for other cationic AMPs (Zhu, Mohapatra, and Weisshaar 2019). System-spanning gelation without condensation (Harmon et al. 2017) is the mechanism we observe here, not phase separation.

Third, the authors misconstrue critical concepts regarding the theory behind and study of biomolecular condensation. Here, are some examples:

“As intrinsically disordered proteins often undergo LLPS, we hypothesized that the Nur-enriched compartments are liquid condensates formed via LLPS. To test this, we performed fluorescence recovery after photobleaching (FRAP) analysis on intracellular C42-FITC in E. coli. Within C42-FITC enriched regions, fluorescence in the bleached regions recovered to the intensity of unbleached regions within 20 seconds, indicating that these structures are dynamic and fluid (Extended Data Fig. 8a).”
FRAP is NOT a proof of condensation, it only reports on the dynamics of molecules. It is not because a molecule has slowed dynamics that is in a condensate, especially for large particles like mRNA-ribosome complexes which inherently have slower dynamics (Schavemaker, Śmigiel, and Poolman 2017). Also, whether one component of a condensate can diffuse rapidly or not, does not mean that the condensate is liquid as a whole (Woodruff et al. 2017).

“Nur peptides target ribosomal condensates via LLPS”
Exogenously peptides seem to localize to parts of the cell where ribosomes are but do not cause their condensation, as evidenced by a lack of change in ribosomal localization (Fig. 2,4). They only thing we see is a slowed diffusion of ribosomes, as is expected (Schavemaker, Śmigiel, and Poolman 2017). When peptides are endogenously expressed (Fig. 3), they do condense—likely with ribosomes—at the cell poles as was previously seen for other cationic proteins expressed in bacteria (Kidane et al. 2025). Whether this latter phenomenon is truly liquid-liquid phase separation is untested, and I would urge the authors to use less specific terms (e.g., condensation). Also, it does not seem that the exogenously applied peptides—which is the real life situation of how bacteria encounter AMPs—does induce condensation, but rather modulates ribosomal dynamics by clustering them.

There are many other instances where the author do not accurately interpret their experimental findings regarding condensation. I would urge the authors to dig deeply into some fantastic overviews on experimental standards in the field: (Alberti et al. 2025; Alberti, Gladfelter, and Mittag 2019)

References:
Alberti, Simon, Paolo Arosio, Robert B. Best, Steven Boeynaems, Danfeng Cai, Rosana Collepardo-Guevara, Gregory L. Dignon, et al. 2025. “Current Practices in the Study of Biomolecular Condensates: A Community Comment.” Nature Communications 16 (1): 7730.
Alberti, Simon, Amy Gladfelter, and Tanja Mittag. 2019. “Considerations and Challenges in Studying Liquid-Liquid Phase Separation and Biomolecular Condensates.” Cell 176 (3): 419–34.
Boeynaems, Steven, X. Rosa Ma, Vivian Yeong, Garrett M. Ginell, Jian-Hua Chen, Jacob A. Blum, Lisa Nakayama, et al. 2023. “Aberrant Phase Separation Is a Common Killing Strategy of Positively Charged Peptides in Biology and Human Disease.” BioRxivorg. https://doi.org/10.1101/2023.03.09.531820.
Bowman, Grant R., Luis R. Comolli, Guido M. Gaietta, Michael Fero, Sun-Hae Hong, Ying Jones, Julie H. Lee, et al. 2010. “Caulobacter PopZ Forms a Polar Subdomain Dictating Sequential Changes in Pole Composition and Function.” Molecular Microbiology 76 (1): 173–89.
Chongsiriwatana, Nathaniel P., Jennifer S. Lin, Rinki Kapoor, Modi Wetzler, Jennifer A. C. Rea, Maruti K. Didwania, Christopher H. Contag, and Annelise E. Barron. 2017. “Intracellular Biomass Flocculation as a Key Mechanism of Rapid Bacterial Killing by Cationic, Amphipathic Antimicrobial Peptides and Peptoids.” Scientific Reports 7 (1). https://doi.org/10.1038/s41598-017-16180-0.
Gray, William T., Sander K. Govers, Yingjie Xiang, Bradley R. Parry, Manuel Campos, Sangjin Kim, and Christine Jacobs-Wagner. 2019. “Nucleoid Size Scaling and Intracellular Organization of Translation across Bacteria.” Cell 177 (6): 1632-1648.e20.
Harmon, Tyler S., Alex S. Holehouse, Michael K. Rosen, and Rohit V. Pappu. 2017. “Intrinsically Disordered Linkers Determine the Interplay between Phase Separation and Gelation in Multivalent Proteins.” ELife 6 (November). https://doi.org/10.7554/eLife.30294.

Kidane, Aaron K., Jacob R. Rosenfeld, Jake D. Johnston, Charlie Dubbeldam, Mohammadreza Paraan, and Allie C. Obermeyer. 2025. “Cationic Amino Acid Identity and Net Charge Influence Condensate Properties in E. Coli.” Biomacromolecules 26 (10): 6475–85.
Kwon, Ilmin, Siheng Xiang, Masato Kato, Leeju Wu, Pano Theodoropoulos, Tao Wang, Jiwoong Kim, Jonghyun Yun, Yang Xie, and Steven L. McKnight. 2014. “Poly-Dipeptides Encoded by the C9orf72 Repeats Bind Nucleoli, Impede RNA Biogenesis, and Kill Cells.” Science (New York, N.Y.) 345 (6201): 1139–45.
Loveland, Anna B., Egor Svidritskiy, Denis Susorov, Soojin Lee, Alexander Park, Sarah Zvornicanin, Gabriel Demo, Fen-Biao Gao, and Andrei A. Korostelev. 2022. “Ribosome Inhibition by C9ORF72-ALS/FTD-Associated Poly-PR and Poly-GR Proteins Revealed by Cryo-EM.” Nature Communications 13 (1): 2776.
Schavemaker, Paul E., Wojciech M. Śmigiel, and Bert Poolman. 2017. “Ribosome Surface Properties May Impose Limits on the Nature of the Cytoplasmic Proteome.” ELife 6 (November). https://doi.org/10.7554/elife.30084.
Sneideris, Tomas, Nadia A. Erkamp, Hannes Ausserwöger, Kadi L. Saar, Timothy J. Welsh, Daoyuan Qian, Kai Katsuya-Gaviria, et al. 2023. “Targeting Nucleic Acid Phase Transitions as a Mechanism of Action for Antimicrobial Peptides.” Nature Communications 14 (1): 7170.
Thappeta, Yashna, Silvia J. Cañas-Duarte, Haozhen Wang, Till Kallem, Alessio Fragasso, Yingjie Xiang, William Gray, et al. 2025. “Glycogen Phase-Separation Drives Macromolecular Rearrangement and Asymmetric Division in E. Coli.” The EMBO Journal 44 (24): 7434–76.
Woodruff, Jeffrey B., Beatriz Ferreira Gomes, Per O. Widlund, Julia Mahamid, Alf Honigmann, and Anthony A. Hyman. 2017. “The Centrosome Is a Selective Condensate That Nucleates Microtubules by Concentrating Tubulin.” Cell 169 (6): 1066-1077.e10.
Zhu, Yanyu, Sonisilpa Mohapatra, and James C. Weisshaar. 2019. “Rigidification of the Escherichia Coli Cytoplasm by the Human Antimicrobial Peptide LL-37 Revealed by Superresolution Fluorescence Microscopy.” Proceedings of the National Academy of Sciences of the United States of America 116 (3): 1017–26.

Reviewer #3

(Remarks to the Author)
In this study by Nishinami et al, the authors dissected the bacteria killing mechanism of peptides derived from Nemuri, which was originally discovered in Drosophila as a sleeping-inducing protein with antimicrobial activities by one of the leading authors of this manuscript. They discovered that shorter versions of peptides derived from Nemuri have broad antimicrobial activities but with low toxicity towards mammalian cells, indicating that these peptides have good potential as antibiotics in clinical settings. The authors further found that these Nemuri-derived peptides bind to RNA and induce formation of RNA/peptide condensates via its RG-rich sequences. In bacteria, these peptides penetrate cell membranes and co-condensate with bacterial ribosomes via rather nonspecific (and so more generic) peptide/RNA interactions. Importantly, these peptides can decrease the dynamics of bacterial ribosome condensate and concomitantly inhibit protein translation. Thus, the Nemuri-derived peptides kill bacteria by inhibiting bacterial ribosomes via a distinct mechanism compared to known ribosome targeting antibacterial agents.

This is a very interesting study from angles of antibacterial peptides, phase separation in bacteria, and peptide-ribosome interactions. The experiments in the manuscripts are carefully designed and well executed. The results are clean and properly interpreted. The manuscript is a very strong candidate for NC. I only have two minor comments for the authors to consider.

1. Fig. 2g: It appears that the bacteria cell pole localization of ribosomes upon C42 expression is weakened. Why?
2. Fig. 3: the authors claimed, based on NMR-based peak broadening, that the C42 peptide can self-oligomerize in HEPES buffer. The authors may consider using a light scattering experiment to better demonstrate that the peptide can aggregate in the HEPES buffer (by adding a dye tag to the peptide for scattering detection).

Reviewer #4

(Remarks to the Author)
This manuscript presents exciting data showing that the antimicrobial peptide Nemuri is able to undergo biomolecular condensation, and this may be linked to bacteriocidal activity. Overall this is rather exciting, as this AMP is not directly creating pores in the membrane, but instead entering the cells and colocalizating with ribosomes. In addition, it has broad spectrum activity, and present a rather excited platform for novel antimicrobial development. Much of the work is very well done, but I feel a major gap exists: it is not well established that nucleoid/ribosomes are demixing through phase-separation in E coli. Therefore I feel the results are highly correlative in this regard, without enough evidence to support a mechanism by which the Nur peptides impact polysome phase-separation, which leads to cell death.

Major issues:
It has not been well established that polyribosomes and the nucleoid are able to demix into condensates in bacteria. The evidence for this is not compelling, and FRAP alone does not provide enough evidence to describe the material properties of such examples. It cannot be concluded from this alone that polyribosomes form via LLPS. I would suggest removing this language from the manuscript, as it distracts from the key story about the nur peptides.

Does the potential loss in FRAP recovery of ribosomes occur due to a drop in translation, or an alteration of the resulting nur peptide causing the ribosomes directly to lose dynamics. FRAP of the in vitro reconstituted droplets would be useful in

disentangling this.

Translation assay of measuring GFP fluorescence is indirect, and only measures GFP, not whole cell translation. It would be ideal to examine something more direct, like S35 methionine incorporation as a proxy for global translation.

Interestingly, the Nur peptide has charge patterning that is reminiscent of RNase E's C-terminal IDR (large patches of positive and negative amino acids). This C-terminal IDR is known to drive phase separation of the RNA degradosomes into BR-bodies, which are observed in E coli on the inner membrane. The nur peptide appears to accumulate there at low concentrations (extended Fig 3). It could remain possible that this peptide might also be inhibiting RNase E, as some proteomics reveals RNA degradosome proteins (such as PNPase). Was Rnase E lost in the pelleting of membranes during fractionation of the proteomics experiment? Would a strain lacking the CTD of Rnase E (such as BL21(DE3)* be more or less susceptible to the nur peptides?

Minor comments:
The in vitro droplet assays are interesting, but the addition of poly-lysine is problematic, as that does not exist in E coli. It would be ideal to repeat these assays without poly-lys present. In addition, it would be useful to use a more physiological buffer like polymix used for in vitro translation experiments.

"Harden" seems to improperly describe the FRAP results of ribosomes. I would suggest changing to reduce ribosome dynamics.

The GUV experiments, please justify the lipid composition selected.

Fig 1b - X axis should read concentration, what happened to #2?

Fig 2i - an untreated polysome control for comparison would be very useful to show inhibition of translation.

I feel the discussion of nucleoid/polysome separtion has omitted enough discussion of this paper (10.1016/j.cell.2019.05.017).

Version 1:

Reviewer comments:

Reviewer #2

(Remarks to the Author)
The authors addressed all concerns raised by this reviewer.

Reviewer #4

(Remarks to the Author)
The manuscript is significantly improved, however I still noticed one remaining issue relating to the mechanism of inhibition of translation in the cell.

Fig 6a - The boncat AHA pulse labeling shows only a very mild inhibition of cellular translation (~20%), in opposition to the in vitro translation and GFP translation assays. This is in opposition of the title of the manuscript, "Nemuri-derived antimicrobial peptides that induce ribosomal condensation to arrest translation" and suggests differences in translation of some mRNAs, but likely not translation as a whole.

The turboID found proximity to RhlE and SrmB, and RhlE and SrmB were found to form condensates from Hondele et al. Nature 2019. It would be useful to discuss this in the discussion, as it appears the nemuri peptide may be impacting these condensates, which are well-established ribosome assembly factors. The sucrose gradients shown in 4e appear to show higher C42 intensity in 30S/50S fractions than the A260, while lower C42 intensity in the 70S fraction compared to the A260. Perhaps ribosome assembly is being impacted in the mutant, not global translation. Or perhaps the misregulation of translation of some essential messages?

Open Access This Peer Review File is licensed under a Creative Commons Attribution 4.0 International License, which permits use, sharing, adaptation, distribution and reproduction in any medium or format, as long as you give appropriate credit to the original author(s) and the source, provide a link to the Creative Commons license, and indicate if changes were made.
In cases where reviewers are anonymous, credit should be given to 'Anonymous Referee' and the source.
The images or other third party material in this Peer Review File are included in the article's Creative Commons license, unless indicated otherwise in a credit line to the material. If material is not included in the article's Creative Commons license and your intended use is not permitted by statutory regulation or exceeds the permitted use, you will need to obtain permission directly from the copyright holder.
To view a copy of this license, visit https://creativecommons.org/licenses/by/4.0/

Responses to Reviewers

For clarity and ease of review, the reviewers' comments are reproduced in black, and our responses are provided in blue.

Reviewer 1

Reviewer comment 1.1: The growing increase in multidrug resistant bacteria represents a global threat that highlights the urgent need for new antimicrobial agents. One alternative to small molecule antibiotics is the development of antimicrobial peptides (AMPs) for therapeutic use. While most AMPs kill bacteria by disrupting the cell membrane, specific subclass of AMPs have been identified that pass through the membrane and target intracellular processes.

The manuscript of Nishinami and coworkers follows previous work identfying a Drosophila peptide termed Nemuri (Nur) that can kill bacteria, however, the mechanism has remained unclear. In this manuscript, the authors demonstrate that Nur undergoes proteolytic cleavage upon expression and that the C-terminal region (C62) is responsible for the killing activity and contains two subpeptides C20 and C42 that display similar MIC. The peptides are shown to have broadspectrum activity against a range of Gram-positive and Gram-negative bacteria, with C62 being the most active, followed by C42 and C20. The peptides did not display toxicity against Drosophila S2R+, murine L-1210 and human PANC-1 cells. The authors then focus on the C42 peptide (why not the most active C62 peptide?) and show that it associates with ribosomes and inhibits translation. Rather then a specific inhibition, the authors suggest that the intrinsically disordered regions of the peptide target ribosomal liquid condensates and reduce their fluidity.

Overall, the manuscript is well written, results nicely presented and appropriately interpreted. The finding of novel peptides causing ribosomal condensation will be of general interest to the translation community. I have only a series of comments, which if addressed, should improve the clarity of the manuscript.

Response: We thank the reviewer for these insightful and constructive comments. We have addressed each point in the revised manuscript, as detailed below.

Regarding the reviewer's question about why we focused on C42 rather than the more active C62, there are two main reasons. First, fluorescently labeled C42 could be chemically synthesized, whereas fluorescently labeled C62 was technically challenging to prepare. Second, while C62 showed stronger AMP activity, it showed modest cytotoxicity at the highest concentrations. C42 retained broad-spectrum activity and showed no detectable cytotoxicity under the conditions tested. We therefore considered C42 to be a suitable representative peptide for mechanistic analyses and have now clarified this rationale in the revised manuscript (**Page 5, Lines 167–172**).

To further address whether C62 acts through a mechanism similar to that of C42, we have added new data showing that C62 also suppresses ribosomal dynamics in *E. coli* (**Fig. 5e**). We also confirmed that, like C42, C62 does not cause membrane damage in *E. coli*, as assessed by the SYTOX Green assay (**Fig. 2c**). These additional results support the use of C42 as an experimentally tractable model for investigating the intracellular antimicrobial mechanism of Nur-derived peptides.

Reviewer Comment 1.2: 1. The Nur peptides are bactericidal, yet the experiments only report MIC and no MBC values are shown. Is the bactericidal effect only observed at higher concentrations where membrane damage is observed or is there another mechanism for their bactericidal activity?

Response: We thank the reviewer for raising an important point. To directly evaluate the bactericidal activity of Nur peptides, we performed MBC assays for C62, C42, and C20 and found that the MBC values were nearly identical to their respective MIC values, with MBC/MIC ratios of approximately 1, indicating that these peptides exert bactericidal rather than bacteriostatic activity at concentrations close to the MIC (**Supplementary Table 2**).

We also examined whether this bactericidal activity could be explained by membrane damage. Our SYTOX Green assay showed that C42 did not cause detectable membrane damage even at 3-fold above its MBC. In addition, our new data show that C62 and C20 likewise did not compromise membrane integrity at concentrations up to approximately three times their respective MIC/MBC values (**Fig. 2c**). Together, these results indicate that the bactericidal activity of Nur peptides is unlikely to be driven by membrane disruption, but is instead consistent with an intracellular mechanism of action. We have included these data and the corresponding text in the revised manuscript.

Reviewer Comment 1.3: 2. The Nur peptides inhibit translation in vivo and in vitro, yet I found it difficult to understand if the inhibition of translation is occurring at the same concentrations as the condensate formation i.e. is it possible that these are uncoupled such that translation is inhibited at lower concentrations than where LLPS is observed?

Response: We thank the reviewer for this important comment. We agree that it is important to clarify whether translation inhibition and peptide-induced changes in ribosome organization occur within the same concentration range, or whether these phenotypes could be uncoupled.

In our study, Nur peptides reduced ribosomal mobility in *E. coli* cells at concentrations around the MIC/MBC, where cellular translation was also inhibited, without causing detectable membrane damage. Importantly, this reduction in ribosomal mobility was not observed with other translation inhibitors or antibiotics tested, indicating that it is not simply a general consequence of translation inhibition. In newly added experiments, we further found that Nur peptide treatment induced condensation-like remodeling of ribosome-rich intracellular fractions at these concentrations (**Fig. 5d**). In addition, our *in vitro* reconstitution experiments in Polymix buffer showed that Nur peptides promoted ribosome condensation and nearly abolished ribosomal mobility under defined conditions (**Fig. 5a, f**). These results support a close relationship between translation inhibition, reduced ribosomal mobility, and peptide-induced remodeling of ribosome-rich fractions.

We also quantified intracellular accumulation of C42 using C42-AF488 as a fluorescent proxy. Treatment with 36 µM C42-AF488 resulted in an estimated intracellular peptide concentration of approximately 850 µM, corresponding to approximately 24-fold enrichment over the extracellular concentration (**Supplementary Fig. 2c**). Although this measurement does not directly define the local peptide concentration at ribosomes or the behavior of unlabeled C42, it indicates that C42 can accumulate to high intracellular levels under conditions in which translation inhibition, reduced ribosomal mobility, and condensation-like ribosomal remodeling are observed.

Together, these results support a model in which Nur peptides accumulate inside bacterial cells and engage ribosomes through multivalent interactions. In this model, translation inhibition, remodeling of ribosome-rich fractions, and reduced ribosomal mobility are closely related outcomes of peptide–ribosome engagement, rather than phenotypes that arise in unrelated concentration ranges. We have revised the manuscript to clarify this point while avoiding a strict linear interpretation in which one phenotype must necessarily precede the others.

Reviewer Comment 1.4: 3. In Figure 2k, it would be better to have concentration as the x-axis…Time is not very informative. If more concentrations were tested, then one can determine IC50 easier. Also I don't see replicates anywhere or statistics for signifcance. The 4A peptide has still quite some activity at inhibiting translation – does this correlate with the expected ability to still form condensates?

Response: We thank the reviewer for these helpful suggestions. We performed additional *in vitro* translation assays using a series of C42 concentrations and replaced the original time-course plot in the main figure with a dose-response curve, with peptide concentration shown on the x-axis (**Fig. 3i**). The original time-course data have been moved to the Supplementary Information (**Supplementary Fig. 6e**). This new dose-response analysis allowed us to estimate the IC50 values for translation inhibition: 14.3 µM for WT C42, 18.4 µM for the 4A mutant, and >200 µM for the 16A mutant. We have also added replicate data and statistical analyses to the revised figure and figure legend.

Regarding the 4A mutant, it consistently showed an intermediate phenotype between WT and the inactive 16A mutant. It retained substantial but reduced activity in the *in vitro* translation assay and also showed reduced but detectable RNA binding, intracellular assembly formation, and ribosome clustering activity compared with WT (**Figs. 3g, 4c, and 5a**). Thus, the residual translation-inhibitory activity of 4A is consistent with its partial ability to engage RNA/ribosome-rich assemblies. We have updated the revised manuscript accordingly.

Reviewer Comment 1.5: 4. The in vitro assay is coupled. Perhaps it would be better to use transcribed mRNA and determine directly the IC50 of peptides on translation and separately determine the IC50 for transciption.

Response: We apologize for our unclear descriptions. We would like to clarify that our *in vitro* translation and transcription assays were performed independently, rather than as a coupled transcription-translation assay. In the *in vitro* translation assay, we used pre-transcribed mRNA as the template, so the assay directly measured translation without including a transcription step. Transcription was evaluated separately in an independent *in vitro* transcription assay. We have revised the Methods section for the *in vitro* translation assay to make this experimental design clearer. We have also added IC50 values for translation inhibition, as described above.

Reviewer Comment 1.6: 5. Do condensates still form if one inhibits translation in vivo with other antibiotics, e.g. Ksg or Chl, prior to addition of C42 peptides? Just wondering whether actively translating ribosomes can end up in condensates.

Response: We thank the reviewer for this insightful suggestion. To address whether the translational state of ribosomes affects C42-mediated ribosomal remodeling, we examined the effect of C42 on *E. coli* cells pretreated with rifampicin, kasugamycin, or chloramphenicol. We found that C42-TAMRA did not efficiently penetrate antibiotic-pretreated cells (**Response Fig. 1a**). Consistently, addition of C42 up to 13 μM under these pretreatment conditions did not reduce ribosomal mobility, as assessed by FRAP analysis of L1-msfGFP (**Response Fig. 1b**). These results suggest that the lack of ribosomal mobility reduction is likely explained by impaired intracellular entry of C42 rather than by the translational state of ribosomes alone.

We also observed that C42 readily penetrates log-phase *E. coli* cells but shows reduced intracellular accumulation in stationary-phase cells (**Response Fig. 1c**), suggesting that the physiological state of bacteria influences C42 membrane penetration. Alternatively, antibiotic pretreatment may alter the bacterial envelope or cellular physiology in a way that inhibits peptide entry. Thus, although these experiments do not allow us to conclude whether actively translating ribosomes are intrinsically more susceptible to C42-mediated remodeling, they indicate that intracellular entry of C42 is required for the reduction of ribosomal mobility.

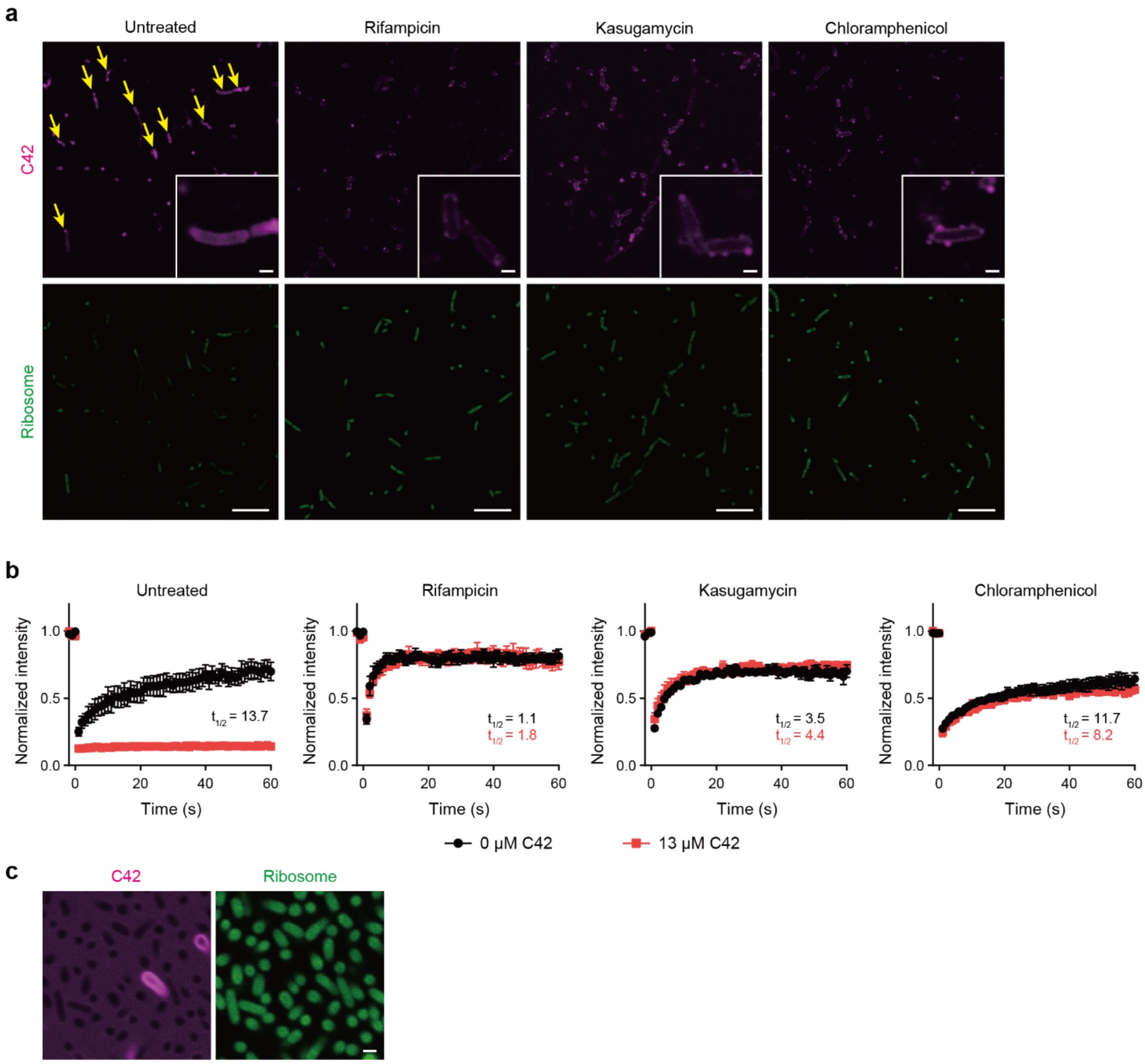

Response Fig. 1. Effect of antibiotic pretreatment on Nur peptide entry and ribosomal mobility.

a, Fluorescence images showing the uptake of C42-TAMRA (final: 20 µM) and the distribution of L1-msfGFP-labeled ribosomes following the indicated antibiotic pretreatments during logarithmic growth. Yellow arrows indicate cells showing intracellular C42-TAMRA accumulation. Scale bars, 10 µm (main images); 1 µm (magnified images). **b**, FRAP recovery curves of L1-msfGFP-labeled ribosomes in *E. coli* CJW7020 cultures with or without pretreatment with rifampicin, kasugamycin, or chloramphenicol during logarithmic growth, followed by treatment with 13 µM C42. Apparent recovery half-times ($t_{1/2}$) are shown. Data are from three independent experiments (*n* = 3). **c**, Fluorescence images of L1-msfGFP-expressing cells treated with 13 µM C42-TAMRA during the logarithmic or stationary growth phase. Scale bar, 1 µm.

Reviewer Comment 1.7: 6. Line 275, the crosslinking experiment is not well explained but appears to crosslink lysines of ribosomal proteins to the peptides - This is not exactly mapping the binding site of the peptides on the ribosome…the peptides interact with RNA and not protein and the ribosomes are predominantly rRNA – can one crosslink the peptides to rRNA and more accurately define the scope of the peptide binding sites on the ribosome.

Response: We thank the reviewer for this important comment. We agree that our original wording overstated what can be concluded from the XL-MS experiment. XL-MS detects cross-links between C42 and lysine residues in ribosomal proteins, and therefore identifies ribosomal protein-proximal contact points rather than direct binding sites on the ribosome or direct contacts with rRNA.

The purpose of this experiment was to assess whether C42 associates with restricted ribosomal regions or shows broad proximity to ribosome-associated surfaces. The observed cross-links distributed across both the 30S and 50S subunits are consistent with broad, multivalent engagement of ribosome-associated surfaces by C42. However, we no longer describe these data as precise mapping of peptide binding sites. This interpretation is supported by our complementary biochemical data showing that C42 associates with ribosomal fractions and binds broadly to RNAs, including rRNA, in an arginine-dependent manner. We have revised the manuscript accordingly.

We agree that direct peptide-rRNA crosslinking would be useful for defining rRNA contact sites more precisely. However, such experiments are technically challenging and require substantial optimization to generate and confidently identify peptide-rRNA crosslinks; therefore, we were not able to complete them within the limited timeframe of this revision. In the revised manuscript, we have therefore avoided site-specific claims and revised the statement “we mapped their binding sites on the ribosome” to “we identified ribosomal protein-proximal contact points compatible with broad ribosome-surface engagement” (**Page 7–8, Lines 267–270**).

Reviewer Comment 1.8: 7. In Fig 1f, the C20 peptide is assigned region 111-172, but presumably this should read 111-131.

Response: We thank the reviewer for the careful reading and for pointing out this error. We have corrected the residue numbering of the C20 peptide in the revised **Fig. 1f**.

Reviewer Comment 1.9: 8. In Figure 4f and ED Fig 9d ribosomes are visualized using EM, however, the results are confusing. In Figure 4f, there appear to be some blobs when ribosomes and C42 are mixed…are these condensates? Do they contain ribosomes or are they just ethane blobs? There don’t appear to be any controls using inactive peptides or positive control…also why does one no see ribosomes in 4f where C42 is not added? In ED Fig 9d there are lots of ribosomes visualized by

negative strain EM but in the absence of C42…where is the experiment with C42 present and other controls? In ED Fig 10b there appear to be lots of aggregates when C42 is mixed with ribosomes and visualized by cryo-EM, but I don't see any controls with ribosomes in the absence of C42. Do the same aggregates form when using 30S or 50S rather than 70S particles?

Response: We thank the reviewer for the careful evaluation. We apologize that our original presentation did not clearly distinguish the different imaging modalities and the intended purpose of each figure. We first clarify that the panel originally shown as **Fig. 4f** (now revised as **Fig. 5a**) shows confocal microscopy images of purified 70S ribosomes, not EM images. Therefore, the observed puncta are not ethane blobs. In the absence of C42, individual ribosomes are not visible by confocal microscopy because they are below the optical resolution limit, whereas C42 addition makes fluorescent ribosome-containing clusters visible. We have revised the figure legend and corresponding text to clarify this point.

The panel originally shown as **Extended Data Fig. 9d** (now presented as **Supplementary Fig. 8c**) shows negative-staining EM images used only as a quality control to confirm the integrity of purified mCherry-labeled ribosomes, and was not intended to show C42-induced ribosome clustering. We have clarified this purpose in the revised figure legend.

For the panel originally shown as **Extended Data Fig. 10b**, we agree that an untreated ribosome control is important. Thus, we have added cryo-EM images of 70S ribosomes without C42 as a control (**Fig. 5b**). These control images show dispersed ribosomal particles, whereas C42-treated samples show clustered ribosomal assemblies, supporting the conclusion that C42 promotes ribosome clustering under these conditions. We have not systematically examined isolated 30S or 50S subunits and therefore do not make conclusions about subunit-specific clustering in the revised manuscript. We have revised the manuscript to clarify these points.

Reviewer Comment 1.10: 9. Perhaps some discussion about the feasibility of these peptides as antibiotics could be added in the discussion section. Generally, one would want the smallest peptide fragment that retains activity but the C20 and C42 already see loses of activity in the MICs. Is it feasible to use the full-length peptide.

Response: We thank the reviewer for this insightful suggestion. We have added a brief discussion of the therapeutic implications of Nur-derived peptides (**Page 12–13, Lines 485–490**). In the revised Discussion, we note that the Nur system generates a suite of active C-terminal peptides, including C62, C42, and the putative C20, which may help optimize the balance between antimicrobial potency and host-cell safety. Among the tested fragments, C62 showed the highest antimicrobial potency, whereas shorter fragments such as C42 and C20 may provide more tractable starting points for future synthesis, modification, and optimization despite their reduced activity. Because full-length Nur contains an N-terminal acidic region that suppresses antimicrobial activity and requires proteolytic processing to generate active C-terminal peptides, the active fragments rather than the full-length protein are likely to be more relevant starting points for therapeutic development. Our sequence-function analysis further suggests that the arrangement of arginine clusters, rather than peptide length alone, is critical for antimicrobial activity, raising the possibility that shorter derivatives with optimized arginine-cluster organization could be developed. At the same time, future optimization will need to balance antimicrobial potency, stability, manufacturability, and host-cell safety.

Reviewer 2

Reviewer comment 2.1: Nishinami et al. investigate the antimicrobial mechanism by cleavage fragments of the nemuri peptide. In previous work, the authors made the riveting discovery that this peptide functions in the regulation of sleep in Drosophila but can moonlight as an antimicrobial peptide (AMP) as well. Here, the authors identify cationic cleavage fragments of the full-length protein that are secreted and responsible for the antimicrobial activity. They continue to explore how these peptides exert their function. AMPs come in many flavors. While the initial focus on the field centered on amphiphilic helical peptides that are membrane active (i.e., their chemistry allows them to insert themselves into membranes to form pores), in recent years accumulating evidence from both prokaryote and eukaryote systems suggests that another class of AMPs is pervasive: disordered cationic peptides. These peptides have been shown by several groups to spontaneously enter cells, without overt membrane disruptions, and subsequently kill cells by binding to RNA and ribosomes, owing to their charge, and shutting down translation. The authors find that the nemuri cleavage fragments follow a similar principle: their evidence strongly suggests that these peptides enter cells, slow down the diffusion of ribosomes (indicating their interaction with them), and halt translation. Unfortunately, the authors failed to properly cite the rich literature that preceded them and had already laid out these biophysical principles in great detail. Was this done to enhance the "novelty" of the work? Additionally, the authors make some puzzling conclusions from their experimental observations and bold claims regarding the role for liquid-liquid phase separation as a major organizational mechanism of the bacterial cytosol. In my opinion, these claims are not grounded in fact or supported by the experimental evidence. I will explain why.

Response: We sincerely thank the reviewer for these important and constructive comments. We appreciate the reviewer's clear explanation of the important body of prior work on cationic peptides that enter cells, engage RNA and ribosome, alter ribosomal mobility, and inhibit translation. We apologize that our original manuscript did not sufficiently acknowledge and discuss these important precedents or clearly distinguish our findings from previously established principles. This was certainly not our intention. We have therefore substantially revised the manuscript to more accurately place our work within this established literature and to define the specific contribution of the present study.

We have also carefully revised the terminology and conceptual framework related to bacterial intracellular organization and biomolecular condensation. In particular, we agree with the reviewer that our original description of ribosome-rich cytosolic regions as pre-existing "ribosomal liquid condensates" was not sufficiently supported. We no longer present untreated bacterial ribosome-rich cytosolic regions as pre-existing ribosomal condensates formed by classical LLPS. Instead, we now describe the basal organization of bacterial cells more conservatively as nucleoid exclusion and spatial segregation of ribosome-rich cytosolic regions (Gray et al., 2019).

At the same time, we retain a more restricted conclusion regarding Nur-treated cells and ribosomes. Specifically, our revised manuscript proposes that Nur-derived peptides induce peptide-dependent condensation and remodeling of ribosome-rich intracellular fractions after entering bacterial cells. This interpretation is supported by new *in vivo* and *in vitro* data added in this revision. Importantly, in response to the reviewer's concern, we performed additional experiments to distinguish the behavior of ribosomes from that of soluble cytoplasmic components (see our responses to **Reviewer 2 Comments 2.3, 2.4, 2.6, and 2.7**). These experiments showed that Nur peptide treatment selectively

induced condensation and remodeling of ribosome-rich intracellular fractions and reduced ribosomal mobility, whereas soluble cytoplasmic markers were not similarly immobilized. These results argue against nonspecific gelation of the cytosol as a whole and instead support a ribosome-centered remodeling process. *In vitro*, Nur peptides directly induced ribosomal condensation and reduced ribosomal mobility under defined buffer conditions. Thus, our revised use of terms such as "condensation" and "phase transition" refers to Nur-induced remodeling of ribosome-rich assemblies, not to pre-existing ribosomal LLPS in untreated bacterial cells. We therefore do not assign the Nur-induced phenotype specifically to classical LLPS *in vivo*.

Accordingly, we have revised the manuscript to shift the conceptual emphasis away from the "hardening" of pre-existing ribosomal condensates and toward Nur peptide engagement with RNA/ribosome-rich intracellular regions, accompanied by reduced ribosomal mobility and translation arrest. The revised manuscript now emphasizes that the contribution of this study lies in identifying Nur-derived cleavage fragments as endogenous AMPs that exploit the biophysical vulnerability of RNA/ribosome-rich intracellular states. Specifically, we link Nur processing, bacterial entry without overt membrane disruption, RNA/ribosome-rich localization, selective reduction of ribosomal mobility, translation inhibition, bactericidal activity, and peptide-induced remodeling of ribosome-rich fractions.

Thus, rather than presenting cell penetration, translation inhibition by cationic peptides, or ribosomal condensation as entirely new general principles, we now frame our findings as revealing how proteolytically generated fragments of the *Drosophila* sleep-inducing peptide Nur act as host-derived disordered cationic AMPs that target RNA/ribosome-rich intracellular states. We have also revised the Discussion to better emphasize the biological significance of Nur as a sleep-inducing peptide whose proteolytic processing generates antimicrobial fragments, thereby linking stress-related sleep biology with innate immune defense. We address the reviewer's specific concerns in detail below.

Reviewer Comment 2.2: First, several papers by now have shown that cationic peptides bind to ribosomes in prokaryote and eukaryote systems. Hereby, they can (1) prevent ribosomal assembly (Kwon et al. 2014), (2) directly impede translation by binding to catalytic sites (Loveland et al. 2022), (3) condense ribosomes via complex coacervation (Sneideris et al. 2023), or (4) cause ribosomal-mediated cytosolic gelation (Zhu, Mohapatra, and Weisshaar 2019). The authors do cite some papers in the introduction but fail to acknowledge the rich detail these papers have already given us into the mechanism of cationic peptides like the ones studied here. Moreover, they especially fail to mention that ribosomal condensation and clustering have been previously reported and studied in great mechanistic and biophysical detail:

(Schavemaker, Śmigiel, and Poolman 2017) - Demonstrated that positively charged proteins bind ribosomes in the bacterial cytosol and slow down their diffusion dynamics.

(Chongsiriwatana et al. 2017) - Demonstrated that cationic AMPs drive ribosome coacervation in bacteria, which correlates with their killing activity.

(Boeynaems et al. 2023) - Demonstrated that cationic peptides can cause the coacervation of many anionic proteins, including ribosomes, in eukaryote and prokaryote systems.

As the main claim of this paper is they discovered a novel mechanism that centers on the condensation of ribosomes. It is puzzling as to why these previous reports were omitted.

References:

1. Kwon, Ilmin, Siheng Xiang, Masato Kato, Leeju Wu, Pano Theodoropoulos, Tao Wang, Jiwoong Kim, Jonghyun Yun, Yang Xie, and Steven L. McKnight. 2014. "Poly-Dipeptides Encoded by the C9orf72 Repeats Bind Nucleoli, Impede RNA Biogenesis, and Kill Cells." Science (New York, N.Y.) 345 (6201): 1139–45.
2. Loveland, Anna B., Egor Svidritskiy, Denis Susorov, Soojin Lee, Alexander Park, Sarah Zvornicanin, Gabriel Demo, Fen-Biao Gao, and Andrei A. Korostelev. 2022. "Ribosome Inhibition by C9ORF72-ALS/FTD-Associated Poly-PR and Poly-GR Proteins Revealed by Cryo-EM." Nature Communications 13 (1): 2776.
3. Sneideris, Tomas, Nadia A. Erkamp, Hannes Ausserwöger, Kadi L. Saar, Timothy J. Welsh, Daoyuan Qian, Kai Katsuya-Gaviria, et al. 2023. "Targeting Nucleic Acid Phase Transitions as a Mechanism of Action for Antimicrobial Peptides." Nature Communications 14 (1): 7170.
4. Zhu, Yanyu, Sonisilpa Mohapatra, and James C. Weisshaar. 2019. "Rigidification of the Escherichia Coli Cytoplasm by the Human Antimicrobial Peptide LL-37 Revealed by Superresolution Fluorescence Microscopy." Proceedings of the National Academy of Sciences of the United States of America 116 (3): 1017–26.
5. Schavemaker, Paul E., Wojciech M. Śmigiel, and Bert Poolman. 2017. "Ribosome Surface Properties May Impose Limits on the Nature of the Cytoplasmic Proteome." ELife 6 (November). https://doi.org/10.7554/elife.30084.
6. Chongsiriwatana, Nathaniel P., Jennifer S. Lin, Rinki Kapoor, Modi Wetzler, Jennifer A. C. Rea, Maruti K. Didwania, Christopher H. Contag, and Annelise E. Barron. 2017. "Intracellular Biomass Flocculation as a Key Mechanism of Rapid Bacterial Killing by Cationic, Amphipathic Antimicrobial Peptides and Peptoids." Scientific Reports 7 (1). https://doi.org/10.1038/s41598-017-16180-0.
7. Boeynaems, Steven, X. Rosa Ma, Vivian Yeong, Garrett M. Ginell, Jian-Hua Chen, Jacob A. Blum, Lisa Nakayama, et al. 2023. "Aberrant Phase Separation Is a Common Killing Strategy of Positively Charged Peptides in Biology and Human Disease." BioRxivorg. https://doi.org/10.1101/2023.03.09.531820.

Response: We sincerely thank the reviewer for this detailed and important comment. We recognize that our original manuscript did not adequately acknowledge the breadth and mechanistic depth of prior studies on cationic peptide interactions with nucleic acids, ribosomes, and other biomolecules. We appreciate the opportunity to more clearly position our findings in relation to these related but distinct prior studies. We have now substantially expanded the manuscript to incorporate the studies highlighted by the reviewer.

These prior studies include work showing that positively charged proteins can interact with ribosomes and alter their diffusion (Schavemaker et al., 2017), that cationic antimicrobial peptides or peptoids can induce broad intracellular biomass flocculation, including aggregation of bacterial ribosomes (Chongsiriwatana et al., 2017), that disease-associated PR/GR peptides can inhibit ribosome function (Kwon et al., 2014; Loveland et al., 2022), that antimicrobial peptides can promote nucleic-acid phase transitions *in vitro* and nucleic-acid clustering in bacterial cells (Sneideris et al., 2023), and that cationic peptides can broadly coacervate anionic biomolecules, including ribosomes, in both prokaryotic and eukaryotic contexts (Boeynaems et al., 2023). In the revised manuscript, these

additions have been incorporated into the Introduction (**Page 3, Lines 65–78**) and Discussion (**Page 12, Lines 454–468)**.

In parallel, we have refined the central claim of our study. We no longer present ribosome-associated clustering, condensation, or reduced mobility as phenomena that are entirely without precedent. Nor do we claim that bacterial ribosomes form pre-existing liquid condensates through classical LLPS in untreated cells. Instead, we now frame our findings within the established literature on disordered cationic peptides and their interactions with RNA, ribosomes, and the bacterial cytoplasm.

Within this revised framework, the specific advance of our study is that Nur-derived peptides represent proteolytically generated, host-derived antimicrobial fragments of a *Drosophila* sleep-associated peptide, and that their antimicrobial activity can now be linked to a sequence of cellular and biochemical events: bacterial entry without overt membrane disruption, preferential accumulation in RNA/ribosome-rich intracellular regions, peptide-induced condensation and remodeling of ribosome-rich assemblies, selective reduction of ribosomal mobility, translation arrest, and broad bactericidal activity. Thus, while prior studies have established important principles of cationic peptide interactions with ribosomes, nucleic acids, and the bacterial cytoplasm, our revised data show that Nur-derived peptides exploit these biophysical vulnerabilities as host-derived AMPs that remodel ribosome-rich intracellular states into low-mobility ribosomal assemblies, thereby coupling ribosome-centered condensation and phase-state remodeling with translation arrest and broad-spectrum antimicrobial activity.

Finally, we have revised the Discussion to emphasize the biological context of Nur. Unlike synthetic polycations, antimicrobial peptides, or pathological PR/GR repeat peptides, Nur is an endogenous *Drosophila* sleep-inducing peptide whose proteolytic processing generates antimicrobial fragments. We therefore frame Nur as a biologically distinct example of a disordered cationic AMP that links stress-related sleep biology with innate immune defense while acting through a ribosome-centered intracellular mechanism. We believe these revisions more appropriately acknowledge the prior literature while clarifying how our study extends it.

Reviewer Comment 2.3: Second, the authors make the bold claim that the cationic peptides they study target pre-existing ribosomal condensates, which subsequently harden. It is my understanding that these claims were made on an incorrect understanding of the cellular organization of bacteria. Let me elaborate. The authors image nucleoids and ribosomes. In actively growing bacteria that have ongoing transcription, it is well documented that the nucleoid forms its own entity that excludes ribosomes. There are countless papers that have illuminated key details of this organizational principle. This is a great example: (Gray et al., 2019). So, if the nucleoid is its own entity, with a certain mesh size and rigidity, it will obviously act as a ribosome exclusion barrier. The same is true for protein-based (Bowman et al., 2010) and glycogen-based (Thappeta et al., 2025) bacterial condensates. Thus, the ribosomes occupy all empty space in a bacterial cell that is not occupied by other condensates or large assemblies. We also call this…the cytosol (i.e., the cytosolic space that is not occupied by large stuff). In this study, the authors label the entire cytosolic space of these bacteria first as “ribosome-rich regions”. Nothing wrong with saying that, but at the end of the day it remains simply the cytosol. However, the authors proceed to call this region “ribosomal liquid condensates”. Why is puzzling. It seems the authors make this conclusion based on three observations that by no

means can be considered proof of the existence of a large ribosomal enriched condensates that takes up the whole cell expect the nucleoid. In other words, I do not think any proof has been shown to justify that we from now on consider the cytosol of any cell as a condensate.

References:

1. Gray, William T., Sander K. Govers, Yingjie Xiang, Bradley R. Parry, Manuel Campos, Sangjin Kim, and Christine Jacobs-Wagner. 2019. "Nucleoid Size Scaling and Intracellular Organization of Translation across Bacteria." Cell 177 (6): 1632-1648.e20.
2. Bowman, Grant R., Luis R. Comolli, Guido M. Gaietta, Michael Fero, Sun-Hae Hong, Ying Jones, Julie H. Lee, et al. 2010. "Caulobacter PopZ Forms a Polar Subdomain Dictating Sequential Changes in Pole Composition and Function." Molecular Microbiology 76 (1): 173–89.
3. Thappeta, Yashna, Silvia J. Cañas-Duarte, Haozhen Wang, Till Kallem, Alessio Fragasso, Yingjie Xiang, William Gray, et al. 2025. "Glycogen Phase-Separation Drives Macromolecular Rearrangement and Asymmetric Division in E. Coli." The EMBO Journal 44 (24): 7434–76.

Response: We thank the reviewer for this important clarification. We agree that our original terminology was inappropriate and could be interpreted as implying that the ribosome-rich cytosolic space in untreated bacterial cells represents a pre-existing ribosomal liquid condensate. This conclusion was not sufficiently supported by our data. In the revised manuscript, we therefore no longer describe the ribosome-rich cytosolic space of untreated bacteria as a pre-existing "ribosomal liquid condensate" or as a product of classical LLPS. We also do not imply that the bacterial cytosol as a whole should be considered a condensate.

Instead, we now describe the basal intracellular organization more conservatively as nucleoid exclusion and spatial segregation of ribosome-rich cytosolic regions. As highlighted by the reviewer, actively growing bacteria exhibit spatial separation between the nucleoid and ribosome-rich cytosolic space, and this organization can be explained in large part by size-dependent volume exclusion and entropy-driven effects associated with the nucleoid meshwork and other large intracellular assemblies (Gray et al., 2019). We have also added discussion of protein- and glycogen-based bacterial condensates that act as exclusion barriers or reorganize intracellular macromolecular distributions, as highlighted by the reviewer (Bowman et al., 2010; Thappeta et al., 2025). We therefore distinguish this basal organization from interaction-driven classical LLPS and have replaced terms such as "ribosomal liquid condensates" and "pre-existing ribosomal condensates" with more conservative terminology, including "ribosome-rich regions", "ribosome-rich cytosolic regions", and "nucleoid-excluded cytosolic space" depending on the context.

Importantly, for Nur-treated cells, we retain a separate and more restricted conclusion. Specifically, our revised manuscript proposes that Nur-derived peptides induce peptide-dependent condensation and remodeling of ribosome-rich intracellular fractions after entering bacterial cells, rather than targeting pre-existing ribosomal condensates. This Nur-induced phenotype is distinct from the basal nucleoid-excluded distribution of ribosomes. In response to the reviewer's concern, we performed additional experiments to distinguish the behavior of ribosomes from that of soluble cytoplasmic components. These experiments showed that Nur peptide treatment selectively induced condensation and remodeling of ribosome-rich fractions and reduced ribosomal mobility, whereas soluble cytoplasmic markers were not similarly immobilized (**Fig. 5e, f and Supplementary Fig. 10f**). These

results argue against nonspecific gelation of the cytosol as a whole and instead support a ribosome-centered condensation and remodeling process. *In vitro*, Nur peptides directly induced ribosomal condensation and reduced ribosomal mobility under defined buffer conditions (**Fig. 5a, f**).

Thus, the revised manuscript separates two concepts that were insufficiently distinguished in the original submission: basal bacterial nucleoid/ribosome spatial organization and Nur-induced condensation and remodeling of ribosome-rich fractions into low-mobility states. Accordingly, in the revised manuscript, terms such as “condensation” and “phase transition” refer specifically to the Nur-induced phenotype, not to pre-existing ribosomal LLPS in untreated bacterial cells. We have revised the manuscript throughout to make this distinction explicit (**Page 11–12, Lines 428–453**).

Reviewer Comment 2.4: (1) Ribosomes recover via FRAP in cells indicating they are liquid part of liquid compartments. Yes, this is true. They are part of the cytosol and will therefore diffuse. I do understand how diffusion of a protein in the cytosol implies that the cytosol is a liquid. I agree with that statement. I just fail to understand why this means we need to describe the cytosol as a whole as a condensate.

Response: We agree with the reviewer. We do not intend to claim that the cytosol as a whole is an interaction-driven condensate. As discussed in our responses to **Comments 2.2 and 2.3**, we have revised the manuscript to describe the basal organization of untreated bacterial cells more conservatively as nucleoid exclusion and spatial segregation of ribosome-rich cytosolic regions, rather than as pre-existing ribosomal liquid condensates.

In addition, we also agree that FRAP should not be used by itself as proof of condensation or LLPS. In the revised manuscript, FRAP is used as a readout of molecular mobility and exchange. Specifically, FRAP analysis before peptide treatment is used to describe the basal mobility of ribosomes within ribosome-rich cytosolic regions. After C42 treatment, we observed a reduction in the mobility of ribosomal L1-msfGFP, whereas soluble cytoplasmic mGFP did not show the same degree of mobility reduction under the same conditions (**Fig. 5e and Supplementary Fig. 10f**). In cells co-expressing L1-msfGFP and cytoplasmic mCherry, C42 induced condensation and remodeling of ribosome-rich fractions, from which soluble mCherry was excluded (**Fig. 5d**). We interpret these results as evidence for Nur-induced ribosome-centered condensation/remodeling and reduced ribosomal mobility, not as evidence that the entire cytosol is a condensate.

Moreover, FRAP analysis of intracellular mGFP-C42 assemblies showed fluorescence recovery, indicating molecular exchange between these assemblies and the surrounding cytosol (**Fig. 4d**). We now interpret this result as evidence that C42-enriched assemblies are dynamic rather than irreversible aggregates, while avoiding the use of FRAP alone as proof of LLPS. We have clarified these interpretations in the revised manuscript.

Reviewer Comment 2.5: (2) The authors can phase separate DNA and RNA (or ribosomes) in vitro by combining it with poly-L-lysine, an extremely charged polymer that will drive their condensation via complex coacervation. These mixtures form multiphase solid-like gels (indicated by the arrested fusion events visible in the pictures). This is something that has been done by countless labs that have generated multiphase assemblies with countless proteins and nucleic acids in the test tube. How any of this can be interpreted as proof that ribosomes in cells can only exist in a condensed state is a

mystery. The fact that the peptides prefer to target the RNA phase of these in vitro assemblies is expected based on the favorable interactions of disordered peptides with ssRNA over DNA (this relates to backbone flexibility).

Response: We agree with the reviewer that the *in vitro* complex coacervation assay using poly-L-lysine does not provide direct evidence for ribosomal condensation in cells. In the revised manuscript, we have therefore stopped using this experiment as evidence that ribosomes exist in a constitutively condensed state in untreated bacterial cells. Instead, we have repositioned the poly-L-lysine-based assay as a supportive biochemical model used to examine how Nur peptides partition within a multiphase nucleic-acid environment. We now clearly distinguish this *in vitro* model from cellular observations and avoid presenting it as direct evidence for pre-existing ribosomal condensation *in vivo*.

To further examine these interactions without relying on highly charged artificial polymers such as poly-L-lysine, we performed additional *in vitro* experiments in ribosome-compatible Polymix buffer, as also suggested by Reviewer 4 (**Comment 4.6**). Under these conditions, C42 induced nucleic-acid assembly formation with an apparent efficiency order of RNA > DNA, as assessed by turbidity measurements and microscopy (**Supplementary Fig. 8d**). In mixed DNA/RNA solutions, C42 promoted the formation of multiphase condensates containing separated DNA- and RNA-rich regions and partitioned more strongly into the RNA-rich phase (**Fig. 5c**). Importantly, C42 and C62 directly induced ribosomal condensation in Polymix buffer in the absence of poly-L-lysine, and C42 reduced ribosomal mobility, whereas the 4A mutant showed an intermediate effect and the inactive 16A mutant failed to induce this phenotype (**Fig. 5a, f**). This sequence-dependent activity is consistent with the antimicrobial activity and RNA/ribosome-engagement properties of these peptides.

Together, these additional experiments support preferential association of Nur peptides with RNA-rich and ribosome-containing environments under defined *in vitro* conditions. Importantly, we now present these data as mechanistic support for Nur peptide–RNA/ribosome interactions and Nur-induced ribosome-centered condensation and remodeling, rather than as proof that ribosomes form pre-existing condensates in untreated bacterial cells. We have revised the corresponding sections of the manuscript accordingly.

Reviewer Comment 2.6: (3) The authors claim to see condensate hardening. I 100% agree that the data support a slowing and even arrest of ribosomal diffusion, as is expected based on the interaction with and crosslinking of ribosomes by these peptides (Schavemaker, Śmigiel, and Poolman 2017). But again, it is not because the ribosomes stop diffusion once a cell becomes overrun with cationic peptides that this meant that the ribosomes needed to exist in a condensed state prior. What the authors essentially observe is the complete gelation of the cytosol as a whole. This is exactly what other have observed and measured before for other cationic AMPs (Zhu, Mohapatra, and Weisshaar 2019). System-spanning gelation without condensation (Harmon et al. 2017) is the mechanism we observe here, not phase separation.

References:

1. Schavemaker, Paul E., Wojciech M. Śmigiel, and Bert Poolman. 2017. "Ribosome Surface Properties May Impose Limits on the Nature of the Cytoplasmic Proteome." ELife 6 (November). https://doi.org/10.7554/elife.30084.

2. Zhu, Yanyu, Sonisilpa Mohapatra, and James C. Weisshaar. 2019. “Rigidification of the Escherichia Coli Cytoplasm by the Human Antimicrobial Peptide LL-37 Revealed by Superresolution Fluorescence Microscopy.” Proceedings of the National Academy of Sciences of the United States of America 116 (3): 1017–26.
3. Harmon, Tyler S., Alex S. Holehouse, Michael K. Rosen, and Rohit V. Pappu. 2017. “Intrinsically Disordered Linkers Determine the Interplay between Phase Separation and Gelation in Multivalent Proteins.” ELife 6 (November). https://doi.org/10.7554/eLife.30294.

Response: We thank the reviewer for this important biophysical clarification. We agree that our phrase “condensate hardening” was inappropriate because it implied that Nur peptides act on pre-existing ribosomal condensates. We have therefore removed this terminology from the revised manuscript. We also agree that reduced ribosomal diffusion does not imply that ribosomes existed in a condensed state before peptide treatment. As discussed in our responses to **Comments 2.3 and 2.4**, we no longer describe untreated ribosome-rich cytosolic regions as pre-existing condensates. Instead, we distinguish basal ribosome-rich cytosolic organization from Nur-induced condensation and remodeling. However, as discussed above, our additional experiments comparing ribosomal and soluble cytoplasmic markers argue against complete, nonspecific gelation of the cytosol as a whole and instead support a ribosome-centered condensation and remodeling process (**Fig. 5d, e and Supplementary Fig. 10f**).

In addition, we appreciate the reviewer’s suggestion to consider the distinction between phase separation, gelation, and percolation as related but distinct physical processes, as highlighted by prior work (Harmon et al., 2017). We also now cite and discuss studies showing that cationic molecules can slow ribosomal diffusion through electrostatic or multivalent interactions with ribosomes (Schavemaker et al., 2017), and that cationic AMPs can induce large-scale cytoplasmic rigidification or gelation-like behavior (Zhu et al., 2019). Thus, we have revised the manuscript to reflect these distinctions and to avoid implying that Nur peptides harden pre-existing ribosomal condensates (**Page 11–12, Lines 428–468**).

Reviewer Comment 2.7: Third, the authors misconstrue critical concepts regarding the theory behind and study of biomolecular condensation. Here, are some examples:

“As intrinsically disordered proteins often undergo LLPS, we hypothesized that the Nur-enriched compartments are liquid condensates formed via LLPS. To test this, we performed fluorescence recovery after photobleaching (FRAP) analysis on intracellular C42-FITC in E. coli. Within C42-FITC enriched regions, fluorescence in the bleached regions recovered to the intensity of unbleached regions within 20 seconds, indicating that these structures are dynamic and fluid (Extended Data Fig. 8a).”
FRAP is NOT a proof of condensation, it only reports on the dynamics of molecules. It is not because a molecule has slowed dynamics that is in a condensate, especially for large particles like mRNA-ribosome complexes which inherently have slower dynamics (Schavemaker, Śmigiel, and Poolman 2017). Also, whether one component of a condensate can diffuse rapidly or not, does not mean that the condensate is liquid as a whole (Woodruff et al. 2017).

“Nur peptides target ribosomal condensates via LLPS”
Exogenously peptides seem to localize to parts of the cell where ribosomes are but do not cause their condensation, as evidenced by a lack of change in ribosomal localization (Fig. 2,4). They only thing

we see is a slowed diffusion of ribosomes, as is expected (Schavemaker, Śmigiel, and Poolman 2017). When peptides are endogenously expressed (Fig. 3), they do condense—likely with ribosomes—at the cell poles as was previously seen for other cationic proteins expressed in bacteria (Kidane et al. 2025). Whether this latter phenomenon is truly liquid-liquid phase separation is untested, and I would urge the authors to use less specific terms (e.g., condensation). Also, it does not seem that the exogenously applied peptides—which is the real life situation of how bacteria encounter AMPs—does induce condensation, but rather modulates ribosomal dynamics by clustering them.

There are many other instances where the author do not accurately interpret their experimental findings regarding condensation. I would urge the authors to dig deeply into some fantastic overviews on experimental standards in the field: (Alberti et al. 2025; Alberti, Gladfelter, and Mittag 2019)

References:

1. Woodruff, Jeffrey B., Beatriz Ferreira Gomes, Per O. Widlund, Julia Mahamid, Alf Honigmann, and Anthony A. Hyman. 2017. “The Centrosome Is a Selective Condensate That Nucleates Microtubules by Concentrating Tubulin.” Cell 169 (6): 1066-1077.e10.
2. Kidane, Aaron K., Jacob R. Rosenfeld, Jake D. Johnston, Charlie Dubbeldam, Mohammadreza Paraan, and Allie C. Obermeyer. 2025. “Cationic Amino Acid Identity and Net Charge Influence Condensate Properties in E. Coli.” Biomacromolecules 26 (10): 6475–85.
3. Alberti, Simon, Paolo Arosio, Robert B. Best, Steven Boeynaems, Danfeng Cai, Rosana Collepardo-Guevara, Gregory L. Dignon, et al. 2025. “Current Practices in the Study of Biomolecular Condensates: A Community Comment.” Nature Communications 16 (1): 7730.
4. Alberti, Simon, Amy Gladfelter, and Tanja Mittag. 2019. “Considerations and Challenges in Studying Liquid-Liquid Phase Separation and Biomolecular Condensates.” Cell 176 (3): 419–34.

Response: We thank the reviewer for this rigorous and constructive guidance regarding the interpretation of biomolecular condensation. We have carefully revised the manuscript in light of the conceptual and experimental standards outlined in the suggested reviews and community commentaries (Alberti et al., 2019; Alberti et al., 2025), as well as the points raised regarding FRAP interpretation (Woodruff et al., 2017). In the revised manuscript, we no longer use FRAP recovery by itself as evidence for LLPS or for the material state of an entire intracellular compartment.

FRAP is now used strictly as a readout of molecular mobility and exchange. For exogenously applied C42, which represents the physiological context in which bacteria encounter antimicrobial peptides, we now describe the cellular phenotype as peptide accumulation in RNA/ribosome-rich regions, Nur-induced condensation and remodeling of ribosome-rich fractions, and reduced ribosomal mobility. We avoid stating that exogenously applied C42 targets pre-existing ribosomal condensates through LLPS, and we distinguish this phenotype from global cytoplasmic immobilization using soluble cytoplasmic fluorescent reporters.

We have also revised the interpretation of intracellular peptide condensates observed during endogenous expression. FRAP analysis of mGFP-C42 condensates showed fluorescence recovery, indicating molecular exchange between these condensates and the surrounding cytosol (**Fig. 4c, d**). We therefore consider these condensates to be dynamic rather than irreversible aggregates. However, we avoid describing them as definitive LLPS solely on the basis of FRAP recovery, and now refer to

them as C42-enriched intracellular condensates or dynamic peptide condensates, depending on the context.

Accordingly, we have revised the terminology throughout the manuscript to avoid overinterpreting condensation-related observations. We now distinguish between basal spatial segregation of ribosome-rich cytosolic regions, Nur-induced condensation and remodeling of ribosome-rich fractions, reduced ribosomal mobility, and dynamic intracellular peptide condensates (**Fig. 5d, e**). We have also incorporated the suggested literature on biomolecular condensate standards, cationic protein condensation in bacteria, and FRAP interpretation (Woodruff et al., 2017; Kidane et al., 2025; Alberti et al., 2019; Alberti et al., 2025). We believe these revisions align our interpretation with current standards in the biomolecular condensate field while preserving the central conclusion that Nur-derived peptides preferentially engage RNA/ribosome-rich intracellular regions and reduce ribosomal mobility in association with translation arrest (**Page 3, Lines 65–79; Page 10, Lines 373–388; Page 11–12, Lines 428–468)**.

Reviewer 3

Reviewer comment 3.1: In this study by Nishinami et al, the authors dissected the bacteria killing mechanism of peptides derived from Nemuri, which was originally discovered in Drosophila as a sleeping-inducing protein with antimicrobial activities by one of the leading authors of this manuscript. They discovered that shorter versions of peptides derived from Nemuri have broad antimicrobial activities but with low toxicity towards mammalian cells, indicating that these peptides have good potential as antibiotics in clinical settings. The authors further found that these Nemuri-derived peptides bind to RNA and induce formation of RNA/peptide condensates via its RG-rich sequences. In bacteria, these peptides penetrate cell membranes and co-condensate with bacterial ribosomes via rather nonspecific (and so more generic) peptide/RNA interactions. Importantly, these peptides can decrease the dynamics of bacterial ribosome condensate and concomitantly inhibit protein translation. Thus, the Nemuri-derived peptides kill bacteria by inhibiting bacterial ribosomes via a distinct mechanism compared to known ribosome targeting antibacterial agents.

This is a very interesting study from angles of antibacterial peptides, phase separation in bacteria, and peptide-ribosome interactions. The experiments in the manuscripts are carefully designed and well executed. The results are clean and properly interpreted. The manuscript is a very strong candidate for NC. I only have two minor comments for the authors to consider.

Response: We sincerely thank the reviewer for the positive and encouraging assessment of our work. We are grateful for the reviewer's recognition of the potential significance of Nur-derived peptides as antimicrobial agents and of their mechanistic link to RNA/ribosome-rich intracellular states and translation inhibition. In the revised manuscript, we have refined the terminology and discussion of peptide-induced remodeling of ribosome-rich fractions to ensure precise and consistent biophysical interpretation throughout the manuscript.

Reviewer comment 3.2: 1. Fig. 2g: It appears that the bacteria cell pole localization of ribosomes upon C42 expression is weakened. Why?

Response: We thank the reviewer for this careful observation. We clarify that this apparent difference in ribosomal localization reflects the different experimental contexts of the two panels. **Fig. 3c**

(originally shown as **Fig. 2g**) shows exogenously applied C42 peptide, whereas **Fig. 3d** (originally shown as **Fig. 2h**) shows mGFP-C42 after intracellular expression and prolonged accumulation. In untreated log-phase cells, ribosomes often appear as patchy or partially polar-enriched signals within the nucleoid-excluded cytosolic space. After exogenous C42 treatment, the DNA/nucleoid region and the ribosome-rich cytosolic region appeared more clearly segregated, which may make the apparent polar enrichment of ribosomes look weaker. In contrast, the mGFP-C42-enriched assemblies shown in **Figs. 3d and 4c, d** arise after intracellular production and prolonged accumulation of mGFP-C42. We have revised the figure legend and corresponding text to more clearly distinguish exogenous C42 application from endogenous mGFP-C42 expression and to clarify that the apparent difference in ribosomal localization reflects differences in peptide delivery route, accumulation time, and extent of intracellular accumulation.

Reviewer comment 3.3: 2. Fig. 3: the authors claimed, based on NMR-based peak broadening, that the C42 peptide can self-oligomerize in HEPES buffer. The authors may consider using a light scattering experiment to better demonstrate that the peptide can aggregate in the HEPES buffer (by adding a dye tag to the peptide for scattering detection).

Response: We thank the reviewer for this helpful comment. Upon careful re-evaluation of the NMR data, we agree that our original interpretation was too strong. The NMR data do not demonstrate self-oligomerization or aggregation of the C42 peptide in HEPES buffer. Rather, the NMR data only suggest that C42 does not adopt a stable folded structure under these conditions. We have therefore revised the manuscript and figure legend to remove the claim that C42 self-oligomerizes or aggregates based on NMR peak broadening. We agree that an additional orthogonal assay, such as light scattering, would be required to support an aggregation claim. Since we do not make such a claim in the revised manuscript, we did not pursue this experiment further.

Reviewer 4

Reviewer comment 4.1: This manuscript presents exciting data showing that the antimicrobial peptide Nemuri is able to undergo biomolecular condensation, and this may be linked to bacteriocidal activity. Overall this is rather exciting, as this AMP is not directly creating pores in the membrane, but instead entering the cells and colocalizating with ribosomes. In addition, it has broad spectrum activity, and present a rather excited platform for novel antimicrobial development. Much of the work is very well done, but I feel a major gap exists: it is not well established that nucleoid/ribosomes are demixing through phase-separation in E coli. Therefore I feel the results are highly correlative in this regard, without enough evidence to support a mechanism by which the Nur peptides impact polysome phase-separation, which leads to cell death.

Response: We thank the reviewer for these insightful and constructive comments and for recognizing the potential significance of Nur-derived peptides.

We also appreciate the reviewer's important point that nucleoid/ribosome demixing in *E. coli* should not be overinterpreted as evidence for interaction-driven phase separation. In response to this concern, and related comments from Reviewer 2, we have substantially revised our terminology and conceptual framing throughout the manuscript. We no longer describe the native ribosome-rich cytosolic space as a pre-existing "ribosomal liquid condensate" formed through LLPS. Instead, we

now describe bacterial organization as nucleoid exclusion and spatial segregation of ribosome-rich cytosolic regions, which can arise in large part from size-dependent volume exclusion and entropy-driven effects (Gray et al., 2019). Correspondingly, we have revised our interpretation to state that Nur peptides accumulate in RNA/ribosome-rich regions, accompanied by reduced ribosomal mobility and condensation-like remodeling of ribosome-rich fractions. We have revised the corresponding text and mechanistic model to present this interpretation more rigorously (**Page 3, Lines 65–79; Page 11–12, Lines 428–453**).

Reviewer comment 4.2: Major issues: It has not been well established that polyribosomes and the nucleoid are able to demix into condensates in bacteria. The evidence for this is not compelling, and FRAP alone does not provide enough evidence to describe the material properties of such examples. It cannot be concluded from this alone that polyribosomes form via LLPS. I would suggest removing this language from the manuscript, as it distracts from the key story about the nur peptides.

Response: We thank the reviewer for this important suggestion. As described in our response to **Comment 4.1**, we have removed language implying that polyribosomes and the nucleoid demix into pre-existing condensates through LLPS. We now describe the baseline bacterial organization more conservatively as nucleoid exclusion and spatial segregation of ribosome-rich regions. We agree that FRAP is used as a readout of molecular mobility rather than as evidence for LLPS. Thus, we have revised the corresponding text throughout the manuscript to avoid distracting from the central finding that Nur-derived peptides enter bacterial cells, engage RNA/ribosome-rich intracellular regions, and reduce ribosomal mobility in association with translation arrest (**Page 10–11, Lines 391–414; Page 11–12, Lines 428–453**).

Reviewer comment 4.3: Does the potential loss in FRAP recovery of ribosomes occur due to a drop in translation, or an alteration of the resulting nur peptide causing the ribosomes directly to lose dynamics. FRAP of the in vitro reconstituted droplets would be useful in disentangling this.

Response: We thank the reviewer for raising this important point. We agree that distinguishing whether reduced ribosomal mobility is a secondary consequence of translation inhibition or reflects a more direct effect of Nur peptides on ribosomal organization and mobility is an important mechanistic issue. To address this issue, we have clarified existing data and added complementary experiments to distinguish a general consequence of translation inhibition from a more direct effect of Nur peptides on ribosomes.

First, treatment with conventional translation inhibitors, including chloramphenicol and kasugamycin, did not reproduce the strong reduction in ribosomal mobility observed after C42 treatment (**Fig. 5e and Supplementary Fig. 10c–e**), indicating that reduced ribosomal mobility is unlikely to be simply a general consequence of translation inhibition. Second, in reconstituted *in vitro* assays performed in ribosome-compatible Polymix buffer, C42 and C62 promoted ribosome condensation in the absence of ongoing translation, whereas the 4A mutant showed an intermediate effect and the inactive 16A mutant failed to promote this effect (**Fig. 5a**). FRAP analysis of these reconstituted ribosome assemblies further showed strongly reduced fluorescence recovery upon C42 addition (**Fig. 5f**), supporting the idea that Nur peptides can directly restrict ribosomal mobility through multivalent engagement of ribosomes under translation-independent conditions.

We recognize that *in vitro* reconstituted ribosome assemblies do not fully recapitulate the intracellular environment. We therefore present these FRAP results as supportive evidence, together with the cellular FRAP data, antibiotic control experiments, microscopy assays showing peptide-induced condensation-like remodeling of ribosome-rich intracellular fractions, and translation assays. Collectively, these results support a model in which reduced ribosomal mobility is not merely a secondary consequence of translation arrest, but is closely linked to peptide-induced ribosomal remodeling. We have revised the corresponding Results (**Page 10–11, Lines 391–414**) and Discussion (**Page 11–12, Lines 428–453**) sections accordingly.

Reviewer comment 4.4: Translation assay of measuring GFP fluorescence is indirect, and only measures GFP, not whole cell translation. It would be ideal to examine something more direct, like S35 methionine incorporation as a proxy for global translation.

Response: We thank the reviewer for this important suggestion. We agree that GFP fluorescence provides an indirect reporter of translation and does not by itself measure global protein synthesis. To address this point, we performed metabolic labeling of newly synthesized proteins using L-azidohomoalanine (AHA), a non-radioactive methionine analogue, followed by click chemistry. This assay monitors global nascent protein synthesis at the whole-cell level and therefore provides a more direct and global measure of translational activity than the GFP reporter assay. The new results showed that C42 caused a global reduction in nascent protein synthesis, as assessed by AHA metabolic labeling (**Supplementary Fig. 6d**). This reduction was partial during the early treatment window tested, consistent with the time required for intracellular Nur peptide accumulation and ribosome-dynamics remodeling. These data support our conclusion that Nur-derived peptides inhibit bacterial translation beyond the GFP reporter system. We have added these data to the revised manuscript and updated the corresponding text to clarify that the GFP reporter assay and the AHA-based metabolic labeling assay provide complementary evidence for translation inhibition.

Reviewer comment 4.5: Interestingly, the Nur peptide has charge patterning that is reminiscent of RNase E's C-terminal IDR (large patches of positive and negative amino acids). This C-terminal IDR is known to drive phase separation of the RNA degradosomes into BR-bodies, which are observed in E coli on the inner membrane. The nur peptide appears to accumulate there at low concentrations (extended Fig 3). It could remain possible that this peptide might also be inhibiting RNase E, as some proteomics reveals RNA degradosome proteins (such as PNPase). Was Rnase E lost in the pelleting of membranes during fractionation of the proteomics experiment? Would a strain lacking the CTD of Rnase E (such as BL21(DE3)* be more or less susceptible to the nur peptides?

Response: Thank you for raising this interesting possibility. We agree that the charge patterning of Nur peptides and their localization near RNA-rich intracellular regions raise the possibility of a relationship with the RNA degradosome or RNase E-associated BR-bodies. To examine this point, we performed antimicrobial activity assays using E. coli BL21(DE3)*, a strain expressing a C-terminally truncated RNase E. Nur peptides nevertheless showed strong antimicrobial activity in this strain (**Supplementary Fig. 5c**). Specifically, the MIC values of C42 and C62 were 3.5 μM and 4.5 μM, respectively, under our assay conditions, comparable to those observed against other *E. coli* strains tested. As a control, amikacin showed an MIC of 3.5 μM in the same strain. These results

indicate that the RNase E C-terminal region is not absolutely required for susceptibility to Nur peptides under our assay conditions.

Regarding the proteomic analysis, we acknowledge that membrane-associated RNA degradosome components could be underrepresented depending on the fractionation procedure. Therefore, we do not use the proteomic data alone to exclude a possible relationship between Nur peptides and RNA degradosome-related components. Nevertheless, Nur peptides remain active in BL21(DE3)*, and qPCR data do not support accelerated degradation of the GFP reporter mRNA under our assay conditions (**Fig. 3h**). Our data argue against a mechanism that primarily depends on RNase E CTD-mediated BR-body formation. We have clarified this point and added these results to the revised manuscript to discuss the potential relationship between Nur peptides and RNA degradosome-related components more appropriately.

Reviewer comment 4.6: Minor comments: The in vitro droplet assays are interesting, but the addition of poly-lysine is problematic, as that does not exist in E coli. It would be ideal to repeat these assays without poly-lys present. In addition, it would be useful to use a more physiological buffer like polymix used for in vitro translation experiments.

Response: We thank the reviewer for this helpful suggestion. As also discussed in our response to Reviewer 2 (**Comment 2.5**), we performed additional *in vitro* experiments without poly-L-lysine using Polymix buffer as you suggested. Under these conditions, Nur peptides induced nucleic-acid assembly formation in the apparent order RNA > DNA, as assessed by turbidity measurements and microscopy (**Supplementary Fig. 8d**). In mixed DNA/RNA solutions, Nur peptides promoted the formation of multiphase assemblies containing separated DNA- and RNA-rich phases and partitioned preferentially into the RNA-rich phase (**Fig. 5c**). We also found that C42 and C62 promoted ribosome condensation in Polymix buffer in the absence of poly-L-lysine, whereas the 4A mutant showed an intermediate effect and the inactive 16A mutant failed to promote this effect (**Fig. 5a, f**). We have added these data and revised the corresponding text to clarify that the poly-L-lysine-based assay is a supportive model, while the Polymix experiments provide additional evidence under more physiologically relevant buffer conditions.

Reviewer comment 4.7: "Harden" seems to improperly describe the FRAP results of ribosomes. I would suggest changing to reduce ribosome dynamics.

Response: We agree with the reviewer. We have replaced the term “harden” with “reduced ribosomal mobility” throughout the revised manuscript to more accurately reflect our FRAP results.

Reviewer comment 4.8: The GUV experiments, please justify the lipid composition selected.

Response: We thank the reviewer for this helpful comment. We have revised the manuscript to clarify the rationale for the lipid composition used in the GUV experiments. A 60 mol% DOPC/40 mol% DOPG lipid mixture was used. The lipid mixture was selected to approximate key physicochemical features of bacterial-like anionic membranes relevant to peptide–membrane interactions, including the presence of anionic lipids. We have added this justification to the revised manuscript (**Page 6, Lines 202–203**).

Reviewer comment 4.9: Fig 1b - X axis should read concentration, what happened to #2?

Response: We thank the reviewer for pointing this out. We have fixed the x-axis label of **Fig. 1b** to "Concentration (μM)" to more accurately reflect both the Nur peptides and the ampicillin control. Regarding fragment #2, we have revised the figure legend to clarify that its chemical synthesis was unsuccessful.

Reviewer comment 4.10: Fig 2i - an untreated polysome control for comparison would be very useful to show inhibition of translation.

Response: We thank the reviewer for this helpful suggestion. We have added the sucrose density gradient profile of the control lysate without C42-FITC treatment in **Supplementary Fig. 5h**. In this experiment, C42-FITC was added to the lysate after cell disruption, and the primary purpose was to determine which ribosomal or polysome-associated fractions the fluorescently labeled peptide co-fractionates with, rather than to measure changes in polysome profiles caused by peptide treatment in living cells. The added control profile provides a reference for the ribosomal and polysome fractions and clarifies the co-fractionation of C42-FITC with these fractions. We have revised the figure legend and corresponding text to clarify the purpose and interpretation of this experiment.

Reviewer comment 4.11: I feel the discussion of nucleoid/polysome separtion has omitted enough discussion of this paper (10.1016/j.cell.2019.05.017).

Response: We thank the reviewer for this helpful suggestion. We have incorporated the suggested reference (Gray et al., 2019) into the revised manuscript, particularly in the section discussing nucleoid/ribosome spatial organization in bacteria. We now cite this study when describing nucleoid exclusion and the spatial segregation of ribosome-rich cytosolic regions, and have revised the corresponding text to avoid overinterpreting this organization as polysome phase separation or LLPS (**Page 3, Lines 68–73**).

Responses to Reviewers

For clarity and ease of review, the reviewers' comments are reproduced in black, and our responses are provided in blue.

Reviewer 2

Reviewer comment 2.1: The authors addressed all concerns raised by this reviewer.

Response: We sincerely thank the reviewer for confirming that our revisions addressed all of your concerns and for your thoughtful and constructive comments throughout the review process, which greatly improved the clarity and quality of the manuscript.

Reviewer 4

Reviewer comment 4.1: The manuscript is significantly improved, however I still noticed one remaining issue relating to the mechanism of inhibition of translation in the cell.

Response: We thank the reviewer for recognizing the substantial improvement in the manuscript and for raising this remaining mechanistic concern. We have addressed this concern by revisiting the *in vivo* nascent-protein-labeling analysis, independently performing a puromycin incorporation assay, moderating the wording of our conclusions, and expanding the discussion of possible effects on ribosome assembly, as detailed below.

Reviewer comment 4.2: Fig 6a - The boncat AHA pulse labeling shows only a very mild inhibition of cellular translation (~20%), in opposition to the in vitro translation and GFP translation assays. This is in opposition of the title of the manuscript, "Nemuri-derived antimicrobial peptides that induce ribosomal condensation to arrest translation" and suggests differences in translation of some mRNAs, but likely not translation as a whole.

Response: We thank the reviewer for this careful observation. We agree that the original AHA-labeling data, as presented, showed only a modest reduction in nascent protein synthesis and were not sufficient by themselves to support the stronger claim implied by "arrest translation". We therefore re-examined and repeated the assay under optimized click-labeling conditions and found appreciable nonspecific TAMRA fluorescence in the no-AHA control under the original click-labeling conditions. Reducing the TAMRA-alkyne concentration to one-tenth of the concentration recommended by the manufacturer markedly decreased this labeling background. Normalization of the AHA signal to total protein and subtraction of the residual no-AHA control signal revealed a substantial C42-dependent reduction in AHA incorporation, indicating that the background fluorescence under the original assay conditions had partially masked this reduction.

To validate this finding using an independent approach, we performed a puromycin incorporation assay adapted from the SUnSET method (Schmidt et al., *Nat. Methods*, 2009). We found that C42 substantially reduced nascent protein labeling, while the no-puromycin control showed negligible signal (Supplementary Fig. 6d and e). The corresponding Methods and figure legends have been updated accordingly. Thus, two independent *in vivo* labeling approaches support substantial

suppression of global nascent protein synthesis in *E. coli*. These findings are also consistent with the GFP-reporter assay in *E. coli* and the reconstituted cell-free translation assay. Although we cannot exclude transcript-specific differences in sensitivity, the combined results are more consistent with broad suppression of nascent protein synthesis than with an effect narrowly restricted to a small subset of mRNAs. That being said, to avoid overstatement, we changed the title from "arrest translation" to "inhibit translation" and revised the corresponding wording throughout the manuscript.

Reviewer comment 4.3: The turboID found proximity to RhlE and SrmB, and RhlE and SrmB were found to form condensates from Hondele et al. Nature 2019. It would be useful to discuss this in the discussion, as it appears the nemuri peptide may be impacting these condensates, which are well-established ribosome assembly factors. The sucrose gradients shown in 4e appear to show higher C42 intensity in 30S/50S fractions than the A260, while lower C42 intensity in the 70S fraction compared to the A260. Perhaps ribosome assembly is being impacted in the mutant, not global translation. Or perhaps the misregulation of translation of some essential messages?

Response: We thank the reviewer for this important suggestion. We agree that the enrichment of RhlE and SrmB in the TurboID-C42 dataset and the detection of C42 in the 30S and 50S fractions raise the possibility of an additional effect on ribosome assembly. We have now explicitly mentioned RhlE and SrmB in the Results and cited the study by Hondele et al. (Nature, 2019; Ref. 85) in the Discussion. We now state that these ribosome-assembly helicases have been reported to form condensate-like assemblies and that C42 may influence ribosome assembly or related RNA-rich condensates. However, whether C42 directly affects assemblies containing these helicases remains to be determined.

We have also revised the description of the sucrose-gradient experiment and stated that C42-FITC was detected in the 30S, 50S, and 70S ribosomal fractions. However, C42 was added to *E. coli* lysates after cell disruption, and the A260 and FITC signals are displayed on independent axes. Therefore, the relative peak heights cannot be used to infer peptide-to-ribosome stoichiometry or preferential binding to the 30S/50S subunits over the 70S ribosome, and this experiment cannot establish an *in vivo* ribosome-assembly defect.

However, we agree that effects on ribosome assembly or transcript-selective translation remain possible and may contribute to the phenotype. These possibilities are not mutually exclusive with overall translation inhibition, because impaired ribosome assembly could itself contribute to reduced protein synthesis in cells. Importantly, however, the reconstituted cell-free system uses mature 70S ribosomes and does not involve ribosome biogenesis. Therefore, the translation inhibition observed in this system cannot be explained solely by a defect in ribosome assembly. Moreover, C42 directly condenses purified 70S ribosomes and inhibits translation in a reconstituted cell-free system, and the revised AHA data and the new puromycin data support substantial suppression of nascent protein synthesis in cells. In the Discussion, we therefore interpret potential effects on ribosome assembly as an additional, non-mutually exclusive consequence of Nur peptide engagement with ribosomes and RNA-rich assemblies, rather than as the sole mechanism.
